# Turn-engineering tunes the conformational rigidity of β-hairpin AMPs in achieving membrane selectivity and killing drug-resistant ESKAPE pathogens

**DOI:** 10.1039/d5sc06810j

**Published:** 2025-10-07

**Authors:** Priyanka Lahiri, Swati Priyadarshini, Mahak Saini, Muskan Agrawal, Sk Abdul Mohid, Raju S. Rajmani, Vishnu S. M. Ammineni, Pritam Biswas, Aparna Asok, Amit K. Baidya, Anirban Bhunia, Govardhan Reddy, Ranjana Pathania, Jayanta Chatterjee

**Affiliations:** a Molecular Biophysics Unit, Indian Institute of Science Bangalore India jayanta@iisc.ac.in; b Solid State and Structural Chemistry Unit, Indian Institute of Science Bangalore India; c Department of Biosciences and Bioengineering, Indian Institute of Technology (Roorkee) Uttarakhand India; d Department of Chemical Sciences, Bose Institute Unified Academic Campus Kolkata India

## Abstract

Naturally occurring β-hairpin antimicrobial peptides (AMPs) exhibit potent membranolytic activity against bacterial and mammalian cells, limiting their therapeutic development due to the lack of selectivity. This study demonstrates that the reverse turn in these AMPs can be used to dictate their molecular rigidity, which drives their membranolytic action. By fine-tuning the rigidity at the reverse turn by incorporating a moderately rigid β-II′ turn-inducing motif through *N*-methylation of the amide bond, we achieved selectivity in targeting the bacterial membrane over human red blood cells. The selectivity results from the hairpin-nucleation efficiency of the engineered β-turn within these linear AMPs devoid of disulfide bridges and their interaction with the neutral mammalian and negatively charged bacterial membrane. Such fine-tuning of the structure at the β-turn allowed us to develop molecules derived from naturally occurring toxic AMPs, which displayed selective killing of drug-resistant bacterial pathogens over mammalian cells with *in vivo* efficacy.

## Abstract 2

Introducing structural flexibility into naturally occurring β-hairpin antimicrobial peptides through reverse-turn engineering allows selective binding onto bacterial membranes over mammalian membranes and subsequent membranolysis.

## Graphical abstract

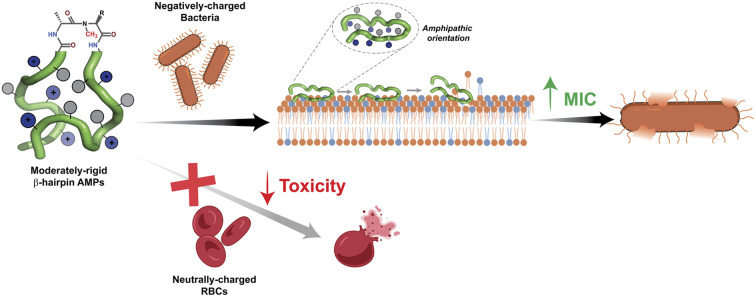

## Introduction

Over the past several decades, imprudent and incessant usage of antibiotics has catalyzed a refractory class of pathogens, colloquially termed “superbugs”. These superbugs demonstrate resistance to numerous last-resort antibiotics like carbapenems, cephalosporins, fluoroquinolones, vancomycin, colistin, *etc.*, and are responsible for a staggering ∼5 million deaths worldwide.^1,2^ In the pursuit of alternate innovative options, a seminal study by Lázár *et al.* identified the ‘Achilles heel’ of an antibiotic-resistant bacterial population.^3^ This vulnerability, termed “collateral sensitivity”, highlighted that the resistant populations exhibit increased susceptibility to antimicrobial peptides (AMPs), thereby positioning AMPs as a promising substitute to the waning antibiotic pipeline.^4^

Among the diverse structural classes of AMPs, β-hairpin peptides are the most potent,^5–7^ and their functions largely depend on their conformational stability.^8–11^ The conformational stability allows these AMPs to effectively interact with and embed within the bacterial membrane and form pores, subsequently disintegrating the membrane.^12^ Naturally occurring β-hairpin AMPs harbor, on average, two disulfide bridges, which have strategically evolved – one adjacent to the reverse turn and another near the termini, for inducing hairpin conformation and providing conformational rigidity, respectively. However, despite being central to the antimicrobial activity, such conformational rigidity is demonstrated to be one of the drivers of cytotoxicity by retaining the hydrophobicity and amphipathicity of the AMP.^13–15^

To reduce the toxicity of naturally occurring β-hairpin AMPs and increase their therapeutic index (*i.e.* the ratio of minimum hemolytic concentration to minimum inhibitory concentration), removal of disulfide bridges has been used frequently. However, the loss of the β-hairpin structure compromises the antimicrobial (membranolytic) potency of these AMPs.^16–19^ Additionally, in reducing environments such as blood plasma or in the presence of suitable nucleophiles, these disulfide bridges are susceptible to scrambling or reduction that can compromise their biological activity, limiting their *in vivo* usage.^20–22^ Therefore, we sought to regain the antimicrobial potency of such linearized peptides by incorporating a constrained β-turn motif, previously developed in our laboratory.^23,24^ Our results suggest that introduction of a moderately rigid β-turn motif as opposed to a strong one is effective in regaining the lost antimicrobial potency of the linearized naturally occurring β-hairpin AMPs. Furthermore, we could extend this strategy to derive a metabolically stable peptide that showed *in vivo* efficacy in killing drug-resistant Gram-negative bacteria.

## Results and discussion

### Re-engineering β-turns in β-hairpin AMPs

**Fig. 1:**
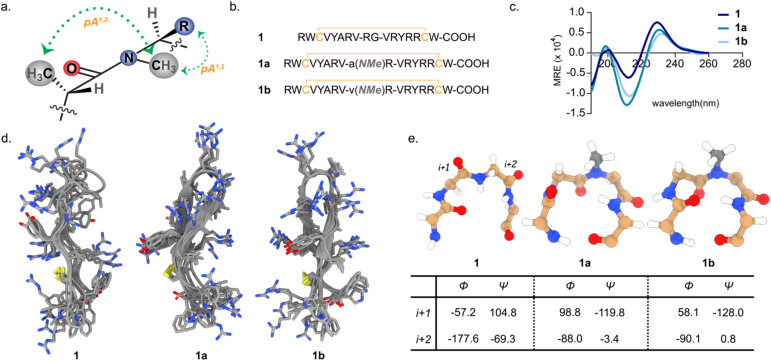
(a) Representation of the pseudoallylic strain (pA^1,2^ and pA^1,3^) induced by *N*-methylation. (b) Sequences of 1, 1a and 1b. (c) CD spectra of 1, 1a and1b in water acquired at 100 μM concentration. (d) Overlay of the 5 lowest energy conformations of 1, 1a and 1b derived by deducing the interproton distances through 2D NMR in 9 : 1 H_2_O : D_2_O, followed by a restrained molecular dynamics simulation for 200 ns, highlighting the backbone flexibility and the right-handed twist. (e) The β-turns in the average structures of 1, 1a, and 1b with the values of the dihedral angles for *i* + 1 and *i* + 2 residues. The ideal torsion angles for a β-II′ turn are *ϕ*: 60°, *ψ*: −120°; *ϕ*: −80°, *ψ*: 0° for *i* + 1 and *i* + 2, respectively.

β-Turns aid in the reversal of polypeptide chains and thus are crucial in protein folding.^25,26^ Previous work from our laboratory has demonstrated the rigidifying role of amide bond *N*-methylation at the β-turn that engages in pseudoallylic strain (Fig. 1a) and results in the formation of stable β-hairpin.^24^ Such engineering enhanced the stability of a three stranded β-sheet protein, Pin 1 WW domain. The stability of the supersecondary structures resulted from conformational preorganization at the β-turn that subsequently culminated in strong intramolecular hydrogen bonds within the β-strands. Therefore, we initially evaluated if chemically engineered β-turns as opposed to a native β-turn will introduce global conformational rigidity into a β-hairpin AMP and impact its antimicrobial potency and toxicity against human red blood cells.

To test this hypothesis, we chose a rationally designed 17-residue single disulfide-bridged β-hairpin AMP (1) (Fig. 1b) that shows broad spectrum antimicrobial activity with no measurable hemolysis (toxicity).^27^ We substituted the native turn residues -Arg–Gly- in 1 with -d-Ala-NMeArg– (1a) and -d-Val-NMeArg- (1b) that were shown to adopt a β-II′ turn. These motifs were chosen based on their ability to induce 81% (-d-Ala-NMeArg–) and 92% (-d-Val-NMeArg-) β-hairpin folded fractions within a 12-mer linear peptide in aqueous solution.^24^

First, we assessed the secondary structure of the analogs by CD spectroscopy in aqueous solution. All the disulfide bridged analogs (1, 1a, and 1b) showed a broad minimum at 213 nm (Fig. 1c), indicative of an antiparallel β-sheet structure.^23^ However, the intensity of the CD signal was stronger for the engineered turn analogs 1a and 1b. To obtain atomistic details of the impact of different turn motifs on the β-hairpin, we determined the NMR chemical shift indices of the amino acid residues (Fig. S2), and subsequently the solution structures of 1, 1a, and 1b by deducing the interproton distances through 2D NMR followed by a restrained molecular dynamics simulation for 200 ns. Superimposing the five minimum energy conformations (Fig. 1d) revealed substantial flexibility at the native turn in 1, that decreased on introducing the -d-Ala-NMeArg- in 1a. A further reduction in flexibility was noted for -d-Val-NMeArg- in 1b. The adoption of near ideal dihedral angles^28^ at the β-II′ turn (Fig. 1e) resulted in improved registry of the antiparallel β-strands (Fig. S3) and conformational rigidity in 1b compared to that in 1a and 1.

### Impact of turn rigidity on peptide-membrane interaction and toxicity

**Fig. 2:**
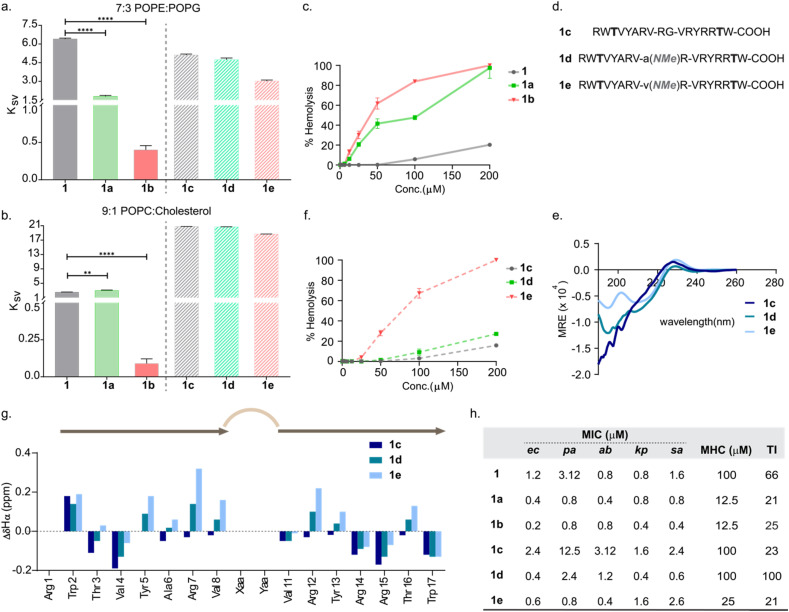
(a) *K*_sv_ (Stern–Volmer constant) plots of liposomes mimicking the bacterial membrane (7 : 3 POPE : POPG). The solid bars represent cyclic 1, 1a and 1b, and the hashed line bars represent linear 1c, 1d and 1e. (b) *K*_sv_ (Stern–Volmer constant) plots of liposomes mimicking the mammalian membrane (9 : 1 POPC : cholesterol). The solid bars represent cyclic 1, 1a and 1b, and the hashed line bars represent linear 1c, 1d and 1e. The one-tailed unpaired *t*-test was applied for statistical significance (*p* = 0.0045(**); *p* < 0.0001(****)) derived from three independent experiments. (c) Hemolysis data of the cyclic 1, 1a and 1b against human red blood cells. (d) Sequences of 1c, 1d, and 1e. (e) CD spectra of 1c, 1d and 1e in water at 100 μM concentration. (f) Hemolysis data of the linear variants 1c, 1d and 1e against human red blood cells. (g) Chemical shift index plot of 1c, 1d and 1e derived through 2D NMR in 9 : 1 H_2_O : D_2_O. (h) Table shows minimum inhibitory concentration (*n* = 5), minimum hemolytic concentration (MHC) (*n* = 3) *i.e.* ≥10% hemolysis and therapeutic index (TI) = MHC/median MIC; the microorganisms used for the MIC study are *Escherichia coli* (ec),*Pseudomonas aeruginosa* (pa), *Acinetobacter baumannii* (ab), *Klebsiella pneumoniae* (kp), and *Staphylococcus aureus* (sa).

To understand how the structural rigidity impacts their association with models of bacterial and mammalian membranes, we evaluated the interaction of these peptides with lipid vesicles (LUVs) mimicking the bacterial (7 : 3 POPE : POPG) and mammalian (9 : 1 POPC : cholesterol) cell-membranes by determining the Stern–Volmer coefficients (*K*_sv_) through acrylamide quenching of tryptophan fluorescence.^29^ The fluorescence of a solvent accessible tryptophan residue can be quenched when in contact with acrylamide. Efficient embedding of a peptide into the vesicle leading to insertion of terminal tryptophan in the lipids prevents the fluorescence quenching of the tryptophan residue, resulting in low *K*_sv_ values, while poor embedding results in high *K*_sv_ values. The lower *K*_sv_ values of the peptides in the presence of LUVs indicate efficient membrane interaction (Fig. S4). With increasing structural rigidity in the order 1 < 1a < 1b, we note better binding to bacterial liposomes (Fig. 2a). Curiously, 1 and 1a show comparable interaction with mammalian liposomes, whereas 1b with the most rigid conformation embeds very efficiently into the mammalian liposomes (Fig. 2b).

We were intrigued to find that 1b with the most rigid conformation, showed very high hemolytic activity against human red blood cells, followed by the moderately flexible 1a; and 1 with a flexible conformation which demonstrated the least toxicity (Fig. 2c). Thus, we note that structural rigidity in the turn increases the global rigidity and positively impacts the membrane interaction of these AMPs, leading to higher toxicity. Therefore, we linearized the peptides to obtain a flexible conformation, by substituting the cysteines with threonines to derive 1c, 1d, and 1e (Fig. 2d). Threonine was chosen to retain a local extended conformation of the β-strands.^30^ While 1c failed to demonstrate a β-hairpin CD signature, the engineered β-turn motifs (1d and 1e) showed a tendency to regain the β-hairpin structure in aqueous solution (Fig. 2e), which was amplified in the presence of SDS-micelles (commonly used as a mimic of the bacterial membrane) (Fig. S5). This was also confirmed by determining the NMR chemical shift indices of the amino acid residues in 1c–1e, where most residues in 1e show a positive (albeit weak) Δ*δHα*, indicating an extended conformation found in β-strands (Fig. 2g).

Subsequently, we assessed the interaction of these peptides with lipid vesicles. All the linear variants showed significantly reduced interaction with the mammalian liposomes in comparison to their cyclic counterparts (Fig. 2b). However, the peptides embedded within the bacterial liposomes rather efficiently compared to the mammalian liposomes, as evident from their respective *K*_sv_ values, which follow the order 1c < 1d < 1e. This suggests that both the conformational rigidity and net charge of the peptide influence their insertion into the bacterial membrane. Thus, 1d with a flexible conformation showed substantially reduced toxicity than 1a (Fig. 2f); however, despite poor interaction of 1e with mammalian lipids, it retained substantial toxicity against human RBCs. This presumably arises from the strong tendency of 1e to adopt a β-hairpin conformation in aqueous solution (Fig. 2e and g) and on interacting with the mammalian membrane.

To verify this conjecture, we resorted to 1 μs all atom molecular dynamics simulation to investigate the interaction of 1a–1e with bacterial (7 : 3 POPE : POPG) and mammalian (9 : 1 POPC : cholesterol) membrane models (see Fig. S6 and the supporting text for detailed analysis). Overall, we note a gradual increase in the β-sheet content of 1 (0.05%), 1a (19.48%), and 1b (38.46%) in the bacterial membrane, whereas in the mammalian membrane, a sharp increase in the β-sheet content of 1b (66.94%) is observed over 1a (9.44%) and 1 (1.51%). This observation correlates with the change in *K*_sv_ values obtained from the quenching studies (Fig. 2a and b). Remarkably, 1c and 1d show very low β-sheet content in sharp contrast to 1e, which shows a β-sheet content of ∼25% in either membrane (Fig. S7). These results are suggestive of a strong turn-nucleating property of -d-Val-NMeArg-, which ultimately results in toxicity in 1b and 1e (Fig. 2c and f).

Next, we evaluated the antimicrobial potency of the peptides against a non-resistant panel of ESKAPE pathogens (Fig. 2h), which indicated that 1a and 1b with a rigid conformation showed improved killing compared to the conformationally flexible 1.^31,32^ Likewise, 1c lacking the conformationally stabilizing disulfide bridge showed the least killing, which showed clear improvement on introducing the turn inducing -d-Ala-NMeArg- (1d) and -d-Val-NMeArg- (1e). The potent antimicrobial activity and toxicity of 1e is in support of the simulation data, which indicates a substantially higher β-sheet content of 1e over 1c and 1d in both bacterial and mammalian membranes. Along with the evidence from CD (Fig. 2e) and NMR (Fig. 2g), we think that the conformationally rigid (strong) β-II′ turn induced by -d-Val-NMeArg-, perhaps allows for the nucleation of a β-hairpin conformation in 1e prior to its interaction with the bacterial and mammalian membrane, leading to its antimicrobial potency and toxicity, respectively. In contrast, 1d with a moderately rigid β-II′ turn (-d-Ala-NMeArg-) interacts poorly with the mammalian membrane and shows low toxicity against the human RBCs, resulting in a therapeutic index of 100 where the therapeutic index is the ratio that reflects the extent to which the inhibitory dose of an AMP is non-toxic to the host red blood cells.

### Versatility of the design strategy and generation of proteolytically stable variants

**Fig. 3:**
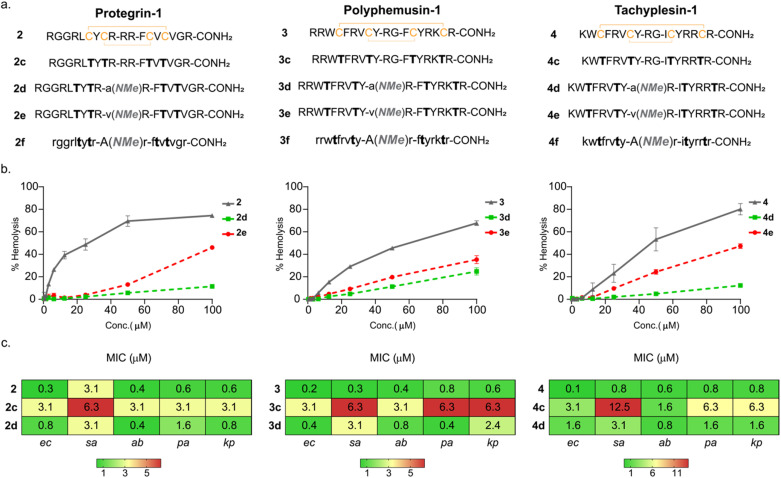
(a) Sequences of protegrin-1(2), polyphemusin-1(3) and tachyplesin-1(4) and their linear variants. The lower-case alphabets denote d-amino acids and (NMe) denotes *N*-methylation. (b) Hemolysis data (*n* = 3) of protegrin-1, polyphemusin-1 and tachyplesin-1 variants against human red blood cells. (c) Table below shows the minimum inhibitory concentration (*n* = 5) of protegrin-1, polyphemusin-1 and tachyplesin-1 variants. The microorganisms used for the MIC study are *Escherichia coli* (ec), *Staphylococcus aureus* (sa), *Acinetobacter baumannii* (ab), *Pseudomonas aeruginosa* (pa), and *Klebsiella pneumoniae* (kp).

Next, we wondered if removal of the conformation stabilizing disulfide bonds and introduction of the β-II′ turn would lead to lower toxicity, yet retain the antimicrobial potency of naturally occurring β-hairpin AMPs, protegrin-1 (2), polyphemusin-1 (3), and tachyplesin-1 (4) (Fig. 3a).^16,33,34^ To test the correlation between turn rigidification and toxicity onto the double-disulfide bridged backbone of 2, 3, and 4, we introduced -d-Ala-NMeArg- (2a, 3a, and 4a) and -d-Val-NMeArg- (2b, 3b, and 4b) (Table 1). Gratifyingly, both the turns increased the toxicity of the parent AMP, with the strong turn -d-Val-NMeArg- having a greater impact on the hemolysis (Fig. S10b). Subsequently, we synthesized the linear variant of these AMPs (2c, 3c, and 4c) (Fig. 3a), which showed significantly reduced toxicity (Fig. S10c). Notably, the moderately rigid β-II′ turn (-d-Ala-NMeArg-) (2d, 3d, and 4d) as opposed to the strong β-II’ turn (-d-Val-NMeArg-) (2e, 3e, and 4e) (Fig. 3a) was successful in retaining the low toxicity of these linear AMPs (Fig. 3b). Gratifyingly, the antimicrobial potency of 2d, 3d, and 4d was substantially better than that of the linear variants (2c, 3c, and 4c) containing the native but weak β-turn motifs (Fig. 3c).

**Table 1:** List of designed peptides with their sequences. The minimum inhibitory concentrations (*n* = 5) are determined against *E. coli*, and the minimum hemolytic concentrations (MHC) (*n* = 3) *i.e.* ≥10% hemolysis are determined against human RBCs. Lower case denotes d-amino acid; NMe denotes *N*-methylation

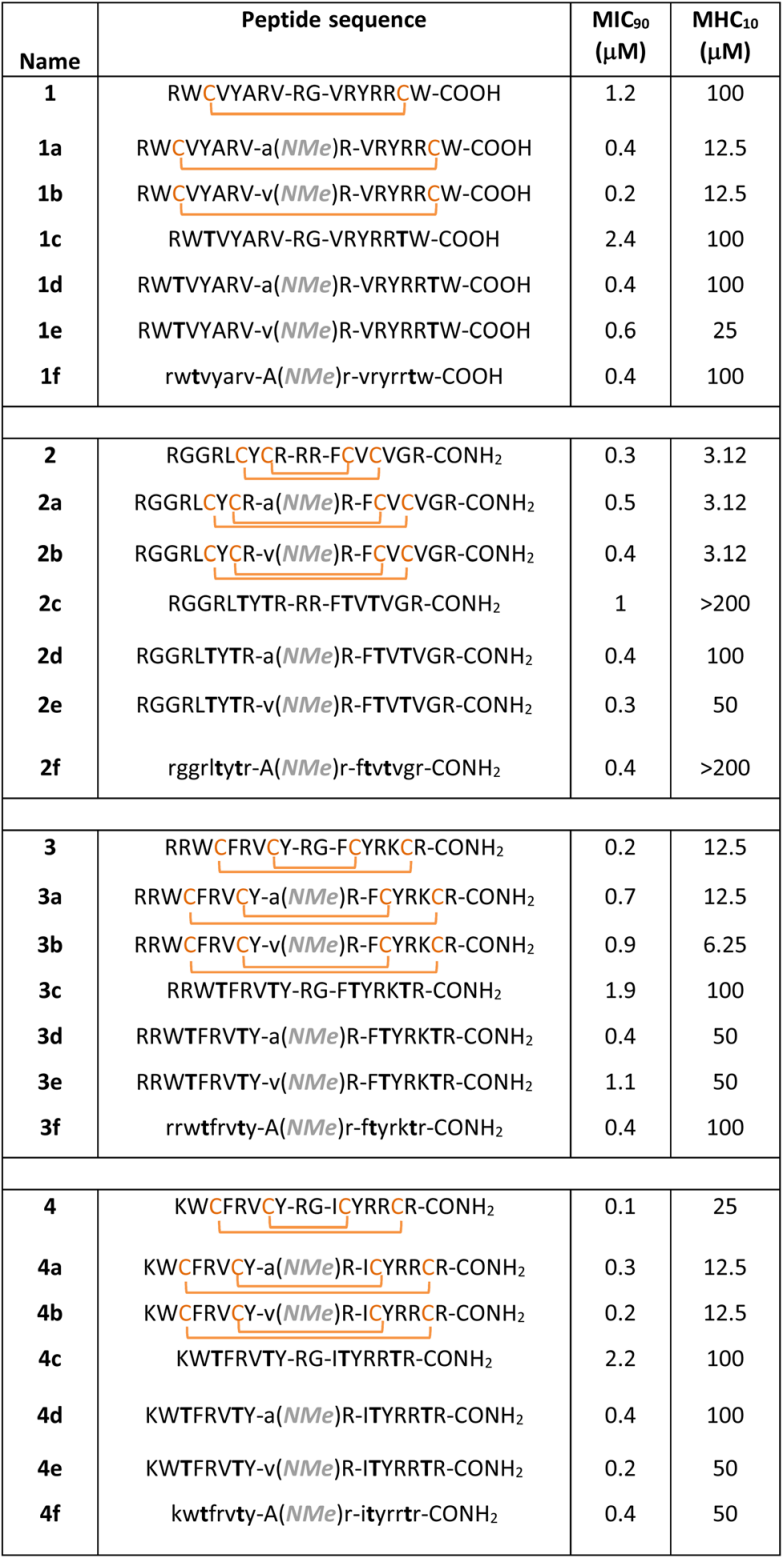

**Fig. 4:**
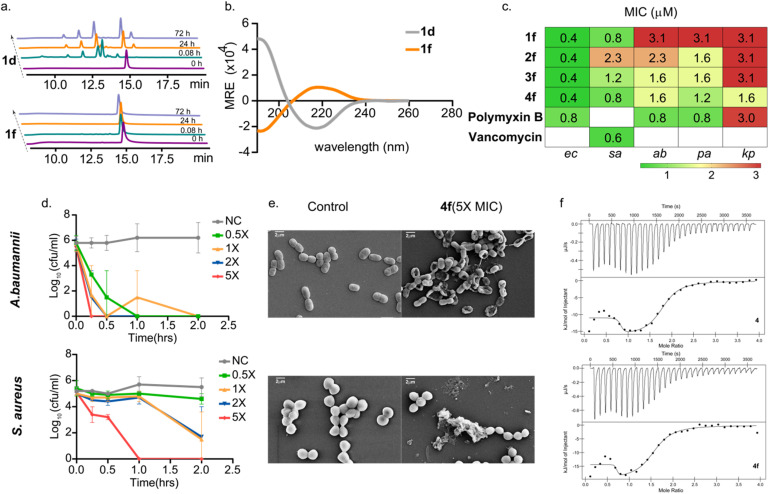
(a) HPLC chromatogram of 1d and 1f subjected to trypsin digestion from 0 h (bottom) to 72 h (up). (b) CD data of 1d and 1f at 25 μM concentration in 10 mM SDS micelles. (c) Table showing MIC values in μM against *Escherichia coli* (ec), *Pseudomonas aeruginosa* (pa), *Acinetobacter baummannii* (ab), *Klebsiella pneumoniae* (kp) and*Staphylococcus aureus* (sa). (d) Time kill-kinetics of bacterial cells exposed to 0.5-, 1-, 2- and 5-times the MIC of 4f for 2.5 h. (e) SEM images after exposure to 5-times the MIC of 4f against *Acinetobacter baummannii* (Gram-negative) and *Staphylococcus aureus* (Gram-positive) for 30 min. (f) ITC thermogram and its fitted titration curve of 4 and 4f with *E. coli* 0111: B4 LPS micelles.

As we were keen to demonstrate the efficacy on the designed AMPs in an animal model of infection, we assessed the proteolytic stability of 1d and 3d against trypsin and chymotrypsin that show differing substrate specificity. Unfortunately, the disulfide-bridged parent peptides (1 and 3) and the linear engineered variants (1d and 3d) show proteolytic degradation within 5 minutes of incubation with the protease (Fig. 4a and S11). Therefore, to obtain protease-resistant peptides, we obtained the enantiomers of 1d–4d by inverting the chirality of the amino acid residues (Fig. S1). The enantiomeric peptides (1f–4f) showed an inversion of the CD signal, indicating the adoption of a mirror image structure (Fig. 4b and S12).^35,36^ Consequently, 1f and 3f showed complete resistance against degradation by the proteases (Fig. 4a and S13). Subsequently, we assessed if the chirality inversion negatively impacted the antimicrobial potency of the mirror image variants against the ESKAPE pathogens (Fig. 4c). Gratifyingly, 1f–4f showed MIC values within a close range of 0.4 to 3 μM, where the inhibitory potency of 4f was comparable to the latest standard of care antibiotics polymyxin B and vancomycin against the Gram-negative and Gram-positive bacteria, respectively.^37,38^ Thus, with a therapeutic index of 45, for adoption of a hairpin structure in *E. coli* LPS micelles and in SDS micelles (Fig. S14), and total protection against proteolysis (Fig. S15), we chose 4f for further development as a therapeutic lead.

### Mode of action of 4f

To evaluate the mechanism of action of 4f on the microbes, we performed the time-kill kinetics assay that reports the onset of action and the bactericidal activity of the AMP (Fig. 4d).^39^ We used 0.5× to 5× MIC values to estimate the killing of *A. baumannii* and *S. aureus*, representing the Gram-negative and Gram-positive pathogens, respectively. *A. baumannii* showed higher susceptibility to 4f as 5× MIC led to rapid killing within 15 minutes, whereas 5× MIC led to a slower killing of *S. aureus*. Such instantaneous killing by 4f indicates a membranolytic action, as observed for most AMPs.^40,41^ Thus, we examined the uptake of propidium iodide (PI), which can only pass through a damaged membrane following the treatment with varying concentrations of 4f. A dose-dependent enhancement in PI uptake within the entire bacterial population was observed by FACS in *A. baumannii* and *S. aureus* within 10 minutes of treatment with 4f (Fig. S16a), directly demonstrating membrane damage. Subsequently, we examined the consequence of membrane damage to the morphology of the bacteria through SEM following treatment with 5× MIC for 30 minutes (Fig. 4e and S16b). The micrographs showed that within 30 minutes, almost all *A. baumannii* cells showed complete membrane rupture, while in *S. aureus*, cellular debris was noted within a significant proportion of the population, corroborating the data obtained from the time-kill kinetics, thus suggesting membrane damage as the major mode of bacterial killing by 4f.

Next, the membrane binding property of 4f was analyzed using ITC (Fig. 4f and S17). For this, *E.coli* LPS micelles were taken as a bacterial membrane surrogate, and we titrated both 4 and 4f against the LPS. The thermograms show exothermic interaction of both 4 and 4f to the LPS. Even though saturation was reached after 20 injections, a minor heat fluctuation after the 4th injection suggests both primary and secondary interaction with the LPS micelles.^42^ The primary interaction shows a stronger affinity (*K*_D_ in nM) than the secondary interaction (*K*_D_ in μM), wherein the stoichiometry for both interactions is 1 : 1 peptide:LPS. Interestingly, despite the absence of disulfide bonds and inversed chirality, 4f shows comparable binding affinity to its structurally rigid β-hairpin parent congener, tachyplesin-1 (4). This further emphasizes the adoption of a comparable bioactive conformation in 4 and 4f, resulting in their equipotent activity against the ESKAPE pathogens (Fig. S18).

**Fig. 5:**
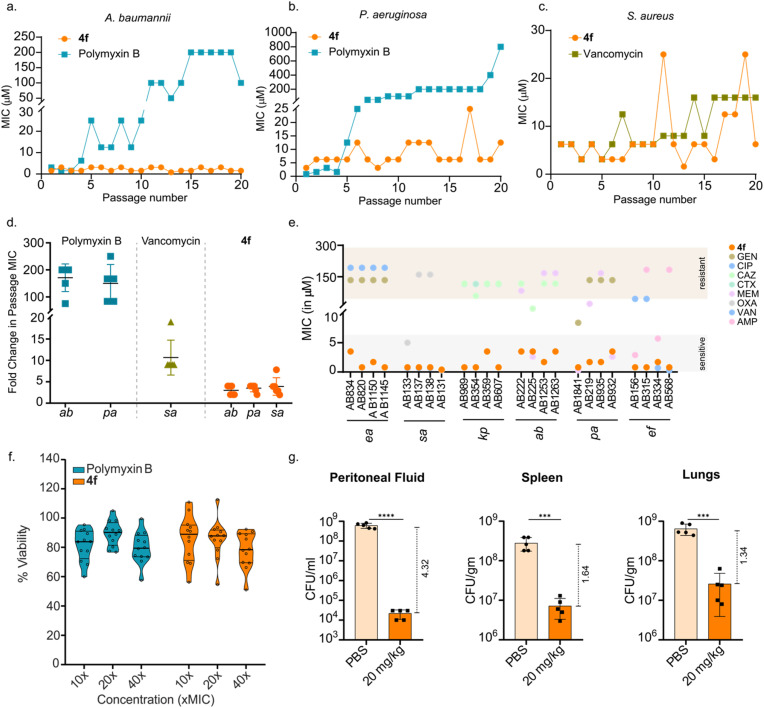
Laboratory-evolved resistance data of (a) *Acinetobacter baummannii* (ab) and (b) *Pseudomonas aeruginosa* (pa) against polymyxin b and 4f, and (c) *Staphylococcus aureus* (sa) against vancomycin and 4f propagated for 20 generations. (d) Fold change in MIC of six independent populations of laboratory-evolved resistant strains of *Acinetobacter baumannii* (ab), *Pseudomonas aeruginosa* (pa) and *Staphylococcus aureus* (sa) over 20 passages. MICs of the 0^th^ and 20th passages were measured and presented as fold-change from the 0^th^ passage. (e) MIC data of 4f and antibiotics (GEN-gentamicin, CIP-ciprofloxacin, CAZ-ceftazidime, CTX-ceftriaxone, MEM-meropenem, OXA-oxacillin, VAN-vancomycin, and AMP-ampicillin) against multidrug resistant strains of *Enterobacter aerogenes* (ea), *Staphylococcus aureus* (sa), *Klebsiella pneumoniae* (kp), *Acinetobacter baummannii* (ab), *Pseudomonas aeruginosa* (pa) and *Enterococcus faecium* (ef). (f) PBMC viability experiment (*n* = 12), where the PBMCs were treated with increasing concentrations of the peptide. (g) Data showing bacterial load in terms of colony-forming units (CFU) per mL for peritoneal fluid and CFU per gm for spleen and lungs. Statistical significance was determined using an unpaired *t*-test with *p* < 0.0001 for peritoneal fluid and *p* < 0.001 for both spleen and lungs (*n* = 5). Log reduction in CFU is indicated by dashed vertical lines next to each plot.

Drug-resistance of 4f and its efficacy against MDR clinical isolates: bacterial pathogens are known to develop rapid resistance against conventional antibiotics.^43^ Thus, we assessed the risk of resistance development under repeated stress of 4f by propagating six independent populations of clinically relevant ESKAPE pathogens: *A. baumannii*, *P. aeruginosa*, and *S. aureus* with increasing concentrations of 4f for 20 generations (Fig. 5a–c).^44^ Polymyxin B and vancomycin were used as positive controls. Remarkably, *A. baumannii* and *P. aeruginosa* failed to develop resistance against 4f in all 6 populations. In contrast, resistance to polymyxin B increased significantly, with median MIC increasing >150-folds for *A. baumannii* and *P. aeruginosa* (Fig. 5d). Despite the challenges associated with the resistance evolution in *S. aureus* against vancomycin in the laboratory,^37^ we could evolve vancomycin-intermediate *S. aureus* (VISA) strains (Fig. 5c), whereas the bacteria was slow to develop resistance against 4f (Fig. 5d). Surprisingly, the induced polymyxin B-resistant *A. baumannii* and *P. aeruginosa* showed no cross-resistance to 4f (MIC 1.6 μM against *A. baumannii* and 2.4 μM against *P. aeruginosa*).

The bacterial population is known to develop AMP resistance by altering their LPS structure, resulting in reduced net negative surface charge.^45,46^ Therefore, we verified that repeated exposure to 4f did not alter the net negative charge of the lab-evolved resistant strains, leading to its retained potency after 20 generations (Fig. S19). Furthermore, we employed the laboratory-evolved polymyxin B and vancomycin-resistant strains in a checkerboard assay to determine potential synergies between these drugs and 4f, which revealed no synergistic or additive effects (Fig. S20).

Encouraged by these findings, we extended our investigation to the Multi Drug Resistant (MDR) clinical isolates of ESKAPE pathogens. We evaluated 4f against 24 such strains alongside two reference antibiotic drugs per strain. To our delight, 4f outperformed the reference antibiotics in most cases, exhibiting MICs ranging from 0.8 to 3.5 μM (Fig. 5e). Since the WHO recognizes *A. baumannii* and *S. aureus* amongst the high-priority critical pathogens, we further identified additional 19 *A. baumannii* and 12 *S. aureus* clinical strains with a drug resistance profile. 4f showed high potency against all the MDR clinical isolates tested, with MICs ranging from 0.5 to 4 μM and 0.25 to 2 μM against *A. baumannii* and *S. aureus*, respectively (Fig. S21). Thus, we note a greater damage to drug-resistant Gram-positive organisms than to Gram-negative ones, emphasizing the challenge associated with developing therapy against Gram-negative organisms.

### *In vivo* efficacy of 4f

To evaluate the *in vivo* efficacy of 4f, we first determined its toxicity against human peripheral blood mononuclear cells (PBMCs), since peripheral blood is where exposure to drugs occur.^47^ PBMCs extracted from the blood of 12 healthy donors were treated with 10×, 20×, and 40× MIC of 4f. We observed a comparable toxicity profile of 4f (median viability ∼85%) and polymyxin B (Fig. 5f). To select an optimal dose for the efficacy studies, we initially performed the acute toxicity assessment at three different doses of 4f, administered intraperitoneally (Fig. S22).^48^ There were no signs of toxicity of 4f at 10 mg kg^−1^; however, at 20 mg kg^−1^, mild symptoms such as wrinkled skin and loss in body weight were observed at 48 h. Nevertheless, weight gain at 60 h through 72 h suggests a transient toxicity of this dose. Visible signs of toxicity such as wrinkled skin, poor motility, and loss in body weight were noted at 40 mg kg^−1^. Nonetheless, none of the doses led to mortality in any of the treatment groups. These results encouraged us to choose 20 mg kg^−1^ for *in vivo* efficacy study.

We then established a peritoneally induced mice infection model using (1 × 10^9^ CFU mL^−1^) MDR *A. baumannii*.^49^ After 2 h of infection, 20 mg kg^−1^ of 4f was administered intraperitoneally to the treatment group. Animals were monitored closely and sacrificed at 8 h post-treatment.^50^ To obtain the bacterial load, lungs, spleen, and peritoneal fluid were harvested from each group (Fig. 5g and S23). Remarkably, peritoneal fluid from the 4f treated group showed a median bacterial load of 3 × 10^4^ CFU mL^−1^, which is 4.3 log reduction compared to that of the untreated control (6.3 × 10^8^ CFU mL^−1^), clearly demonstrating the efficacy of 4f in killing the pathogen *in vivo*.^51^ However, the spleen and lungs from the treatment group showed a 1.64 and 1.34 log reduction in bacterial load compared to the untreated control, respectively. Subsequently, we monitored the survivability of the animals for 24 h post-4f treatment (Fig. S24). It was observed that all 8 animals in the treatment group were healthy and motile, whereas only 1 out of 8 mice survived in the untreated group. Hence, these *in vivo* experiments show that 20 mg kg^−1^ of 4f can significantly reduce bacterial load within 8 h of treatment and successfully alleviate the infection.

## Conclusion

Our investigation highlights the crucial role of β-turns in introducing structural rigidity into disulfide bridged and linear AMPs. Sulfur atoms in proteins are substantially more buried (86%) as opposed to neutral oxygen atoms (40%), suggesting higher atomic lipophilicity of disulfide bonds,^52^ which can potentially promote better interaction with bacterial and mammalian lipids. Thus, we substituted the cysteines with threonine residues to bring about increased microenvironment polarity as well as compatibility with the extended conformation of the β-hairpin AMPs. We note that AMPs devoid of disulfide bonds but harboring a strong-turn -d-Val-NMeArg-, display potent toxicity against human red blood cells by inducing a β-hairpin conformation of the peptide on interacting with the mammalian membrane. In contrast, the native turns in naturally occurring β-hairpin AMPs fail to induce a rigid conformation in the absence of disulfide bonds and thus are devoid of toxicity. Therefore, introduction of a moderately rigid β-turn -d-Ala-NMeArg-, due to its inefficiency in nucleating a β-hairpin conformation on the mammalian membrane achieves greater selectivity in binding to the bacterial membrane. The selectivity stems from the strong binding of the AMP to the POPE and POPG on the bacterial membrane. Thus, we note that greater structural rigidity leads to lower selectivity of AMPs in targeting bacterial over mammalian membranes. Furthermore, the retained membrane binding, potency, and low toxicity of the metabolically stable mirror-image (enantiomer) analogs highlight the critical role of the global shape of these AMPs as opposed to individual amino acid residues in achieving theselectivity in killing the pathogens.

Our lead molecule, 4f shows *in vivo* efficacy in killing drug-resistant *A. baumannii*; nonetheless, we note a substantial difference in the bacterial load reduction at the site of administration (peritoneal fluid) v/s the internal organs: spleen and lungs. This suggests the inefficient transport of 4f from the peritoneal cavity into the systemic circulation. Furthermore, we observed very high plasma protein binding of 4f through the rapid equilibrium dialysis assay (Fig. S25) that precluded the accurate estimation of plasma concentration of 4f to determine the pharmacokinetic parameters. Both these factors presumably arise from the strong binding of the guanidinium group of arginine in 4f to the negatively charged biomolecules like proteoglycan and phospholipids on the mesothelial cell surface lining the peritoneal cavity and the negatively charged serum albumin, which is the most abundant protein in the blood.^53,54^ Therefore, further work is needed, with special emphasis on the arginine residues in 4f, to improve its *in vivo* efficacy. In conclusion, we present a viable approach to develop potent antimicrobial peptides with low toxicity through minimal modifications of the constituent amino acid residues of naturally occurring β-hairpin AMPs that have been evolutionarily selected.

## Author contributions

P. L., S. P., and J. C. collaborated on all aspects of the project design and conception. P. L. and S. P. synthesized, characterized the peptides and performed NMR studies, acrylamide quenching studies, CD spectroscopy, MIC, hemolysis, and animal infection experiments along with assistance from R. S. R. PBMC assay, MIC assays with MDR strains, and animal toxicity experiments were performed by M. S. under guidance from R. P. Simulation studies were performed by M. A. under guidance of G. R. V. S. M. A. and P. B. assisted with the MIC experiments and preparation of liposomes for quenching studies. A. A. assisted with the FACS experiment. A. K. B. assisted with the SEM experiments. S. A. M. performed the ITC experiment under the guidance of A. B. The manuscript was written by P. L., S. P., and J. C. with contribution from all authors.

## Conflicts of interest

The authors declare no conflict of interest.

## Supplementary Material

SC-OLF-D5SC06810J-s001

## Data Availability

Data for this article, including synthetic and experimental procedures, HPLC traces, mass spectra, and CD and NMR spectroscopic data for characterization of the conformation and structure, simulation methods and results, NOE lists and other supporting experiments have been included in the supplementary information (SI) and are available free of charge on the publisher's website for this article. Supplementary information is available. See DOI: https://doi.org/10.1039/d5sc06810j.
