# Supplementary material for: Turn-engineering tunes the conformational rigidity of β-hairpin AMPs in achieving membrane selectivity and killing drug-resistant ESKAPE pathogens
Source: Chem Sci. 2025 Oct 7;16(44):20884–94. doi: 10.1039/d5sc06810j (PMC12520178; doi:10.1039/d5sc06810j)
Supplement: SC-016-D5SC06810J-s001 [file SC-016-D5SC06810J-s001.pdf]

Supplementary Information (SI) for Chemical Science.
This journal is © The Royal Society of Chemistry 2025

Turn-engineering tunes the conformational rigidity of β-hairpin AMPs in selectively killing drug-resistant ESKAPE pathogens

Priyanka Lahiri†[a], Swati Priyadarshini†[a], Mahak Saini[c], Muskan Agrawal[b], Sk Abdul Mohid[d], Raju S Rajmani[a], Vishnu SM Ammineni[a], Pritam Biswas[a], Aparna Asok[a], Amit K Baidya[a], Anirban Bhunia[d], Govardhan Reddy[b], Ranjana Pathania[c], and Jayanta Chatterjee*[a]

a Molecular Biophysics Unit, Indian Institute of Science, Bangalore, India

b Solid State and Structural Chemistry Unit, Indian Institute of Science, Bangalore, India

c Department of Biosciences and Bioengineering, Indian Institute of Technology (Roorkee), Uttarakhand, India

d Department of Chemical Sciences, Bose Institute, Unified Academic Campus, Kolkata, India

† These authors contributed equally to this work

* Corresponding author E-mail: jayanta@iisc.ac.in

Contents :

Figure S1...S3
Figure S2...S4
Figure S3...S4
Figure S4...S5
Figure S5...S5
Figure S6...S6
Figure S7...S7
Figure S8...S7
Figure S9...S8
Figure S10...S9
Figure S11...S9
Figure S12...S10
Figure S13...S11
Figure S14...S11
Figure S15...S12
Figure S16...S12
Figure S17...S13
Figure S18...S13
Figure S19...S14
Figure S20...S14
Figure S21...S15
Figure S22...S15
Figure S23...S16
Figure S24...S17
Figure S25...S17
Table S1...S18
Table S2...S18
Table S3...S18
Table S4...S18
Table S5...S19
HPLC Chromatogram...S20-S28
MALDI-MS profiles...S29-S56
^{1}H NMR spectra...S57-S58
NOE lists...S58-S63
Experimental Section...S63-S76
References...S77-S78

Name	Peptide Sequence	Charge	Calculated / Observed mass	Therapeutic Index (TI)
**1**	RWCVYARV-RG-VRYRRCW-COOH	+6	2283.17 / 2284.47	83
**1a**	RWCVYARV-a***(NMe)***R-VRYRRCW-COOH	+6	2311.20 / 2312.37	31
**1b**	RWCVYARV-v***(NMe)***R-VRYRRCW-COOH	+6	2339.24 / 2340.38	63
**1c**	RWTVYARV-RG-VRYRRTW-COOH	+6	2281.27 / 2282.63	42
**1d**	RWTVYARV-a***(NMe)***R-VRYRRTW-COOH	+6	2309.30 / 2310.5	250
**1e**	RWTVYARV-v***(NMe)***R-VRYRRTW-COOH	+6	2337.33 / 2338.77	42
**1f**	rwtvyarv-A***(NMe)***r-vryrrtw-COOH	+6	2309.30 / 2309.6	250
**2**	RGGRLCYCR-RR-FCVCVGR-$CONH_2$	+6	2155.06 / 2156.47	10
**2a**	RGGRLCYCR-a***(NMe)***R-FCVCVGR-$CONH_2$	+5	2088.04 / 2085.92	6
**2b**	RGGRLCYCR-v***(NMe)***R-FCVCVGR-$CONH_2$	+5	2112.04 / 2114.33	8
**2c**	RGGRLTYTR-RR-FTVTVGR-$CONH_2$	+6	2151.24 / 2152.62	>200
**2d**	RGGRLYTR-a***(NMe)***R-FTVTVGR-$CONH_2$	+5	2079.19 / 2081.37	250
**2e**	RGGRLTYTR-v***(NMe)***R-FTVTVGR-$CONH_2$	+5	2108.23 / 2109.8	167
**2f**	rggrltytr-A***(NMe)***r-ftvtvgr-$CONH_2$	+5	2079.19 / 2081.26	>500
**3**	RRWCFRVCY-RG-FCYRKCR-$CONH_2$	+7	2453.17 / 2455.52	63
**3a**	RRWCFRVCY-a***(NMe)***R-FCYRKCR-$CONH_2$	+7	2481.20 / 2482.53	18
**3b**	RRWCFRVCY-v***(NMe)***R-FCYRKCR-$CONH_2$	+7	2509.23 / 2510.33	7
**3c**	RRWTFRVTY-RG-FTYRKTR-$CONH_2$	+7	2449.35 / 2450.25	53
**3d**	RRWTFRVTY-a***(NMe)***R-FTYRKTR-$CONH_2$	+7	2477.39 / 2477.52	125
**3e**	RRWTFRVTY-v***(NMe)***R-FTYRKTR-$CONH_2$	+7	2505.42 / 2506.74	45
**3f**	rrwtfrvty-A***(NMe)***r-ftyrktr-$CONH_2$	+7	2477.39 / 2478.49	250
**4**	KWCFRVCY-RG-ICYRRCR-$CONH_2$	+6	2263.08 / 2265.09	250
**4a**	KWCFRVCY-a***(NMe)***R-ICYRRCR-$CONH_2$	+6	2291.12 / 2292.38	42
**4b**	KWCFRVCY-v***(NMe)***R-ICYRRCR-$CONH_2$	+6	2319.15 / 2320.17	63
**4c**	KWTFRVTY-RG-ITYRRTR-$CONH_2$	+6	2259.27 / 2260.39	45
**4d**	KWTFRVTY-a***(NMe)***R-ITYRRTR-$CONH_2$	+6	2287.30 / 2288.79	250
**4e**	KWTFRVTY-v***(NMe)***R-ITYRRTR-$CONH_2$	+6	2315.33 / 2316.75	250
**4f**	kwtfrvty-A***(NMe)***r-ityrrtr-$CONH_2$	+6	2287.30 / 2288.54	125

Figure S1: List of designed peptides with their sequence, net charge and molecular weight in Da (both calculated MW and observed MW in MALDI-TOF are represented). Lower case denotes D-amino acid; *NMe* denotes N-methylation. Therapeutic Index (TI) = MHC/(MIC against *e.coli*) is listed for each peptide.

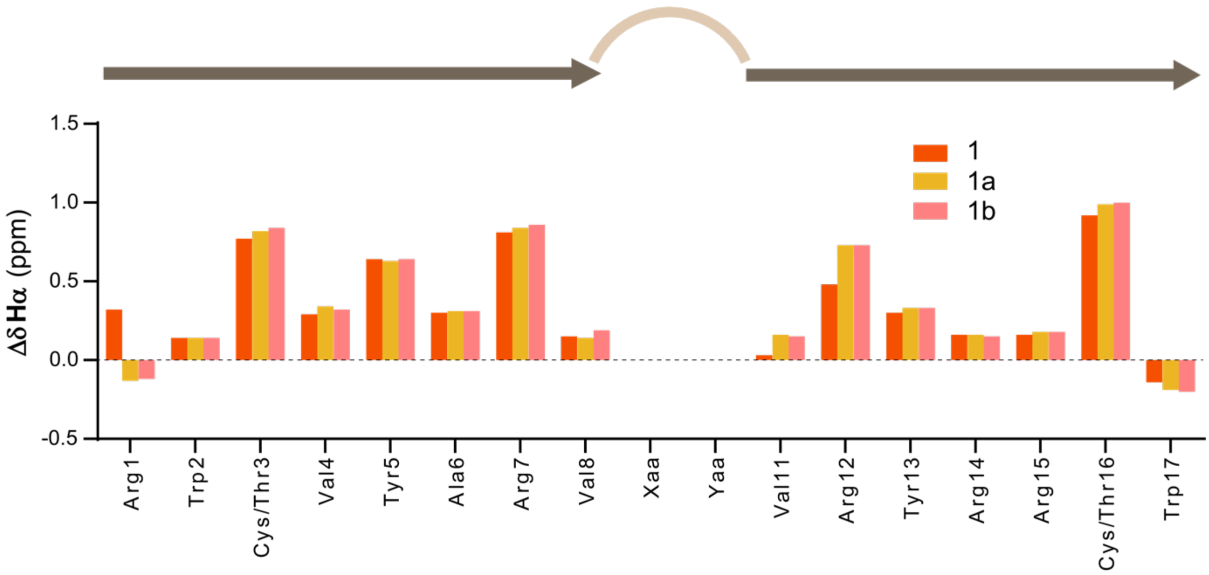

Figure S2: Chemical shift index plot of **1**,**1a** and **1b** from NMR acquired in 9:1 H_2O:D_2O.

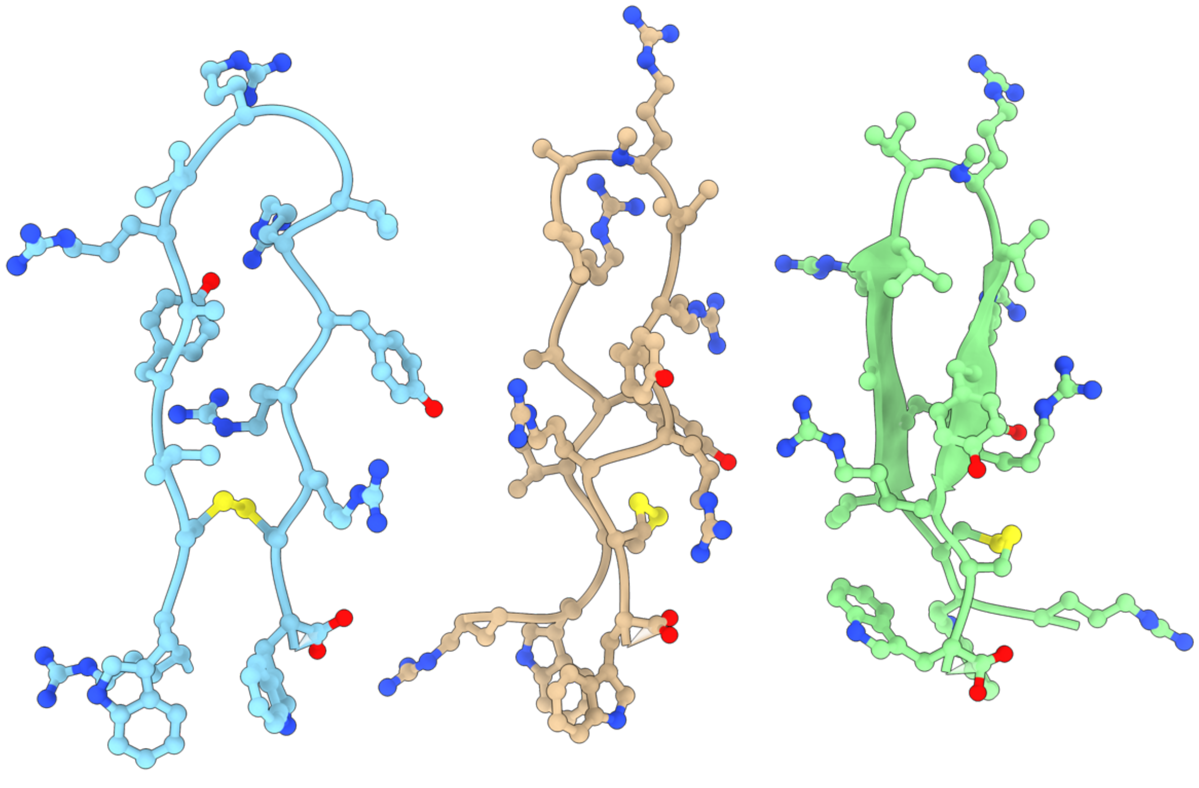

1 **1a** **1b**

Figure S3: Average of NMR solution conformation of **1**, **1a**, and **1b**.

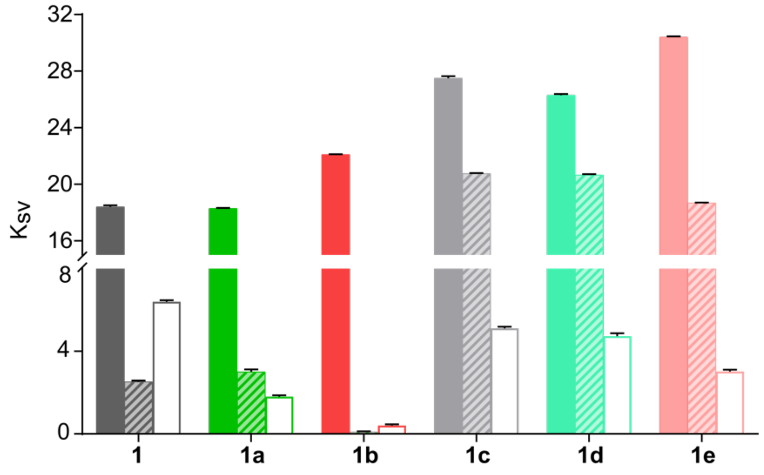

Figure S4: K_{sv} (Stern-Volmer constant) plot of peptides **1**, **1a**, **1b**, **1c**, **1d** and **1e** where 7:3 POPE:POPG liposomes data represented as empty bars, 9:1 POPC:Cholesterol liposomes data represented as hashed lines bar and without liposomes data represented as solid bars.

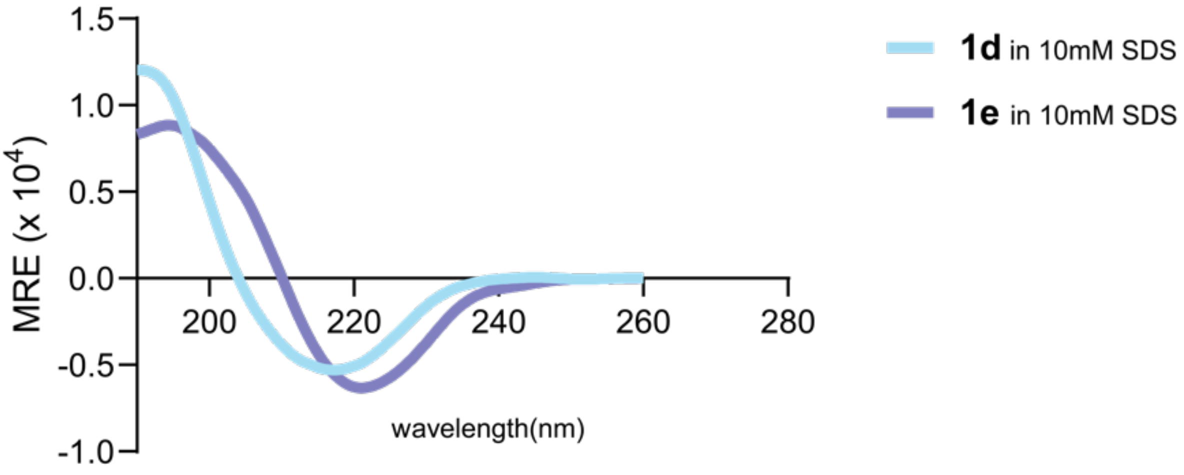

Figure S5: CD spectra of **1d** and **1e** in 10 mM SDS.

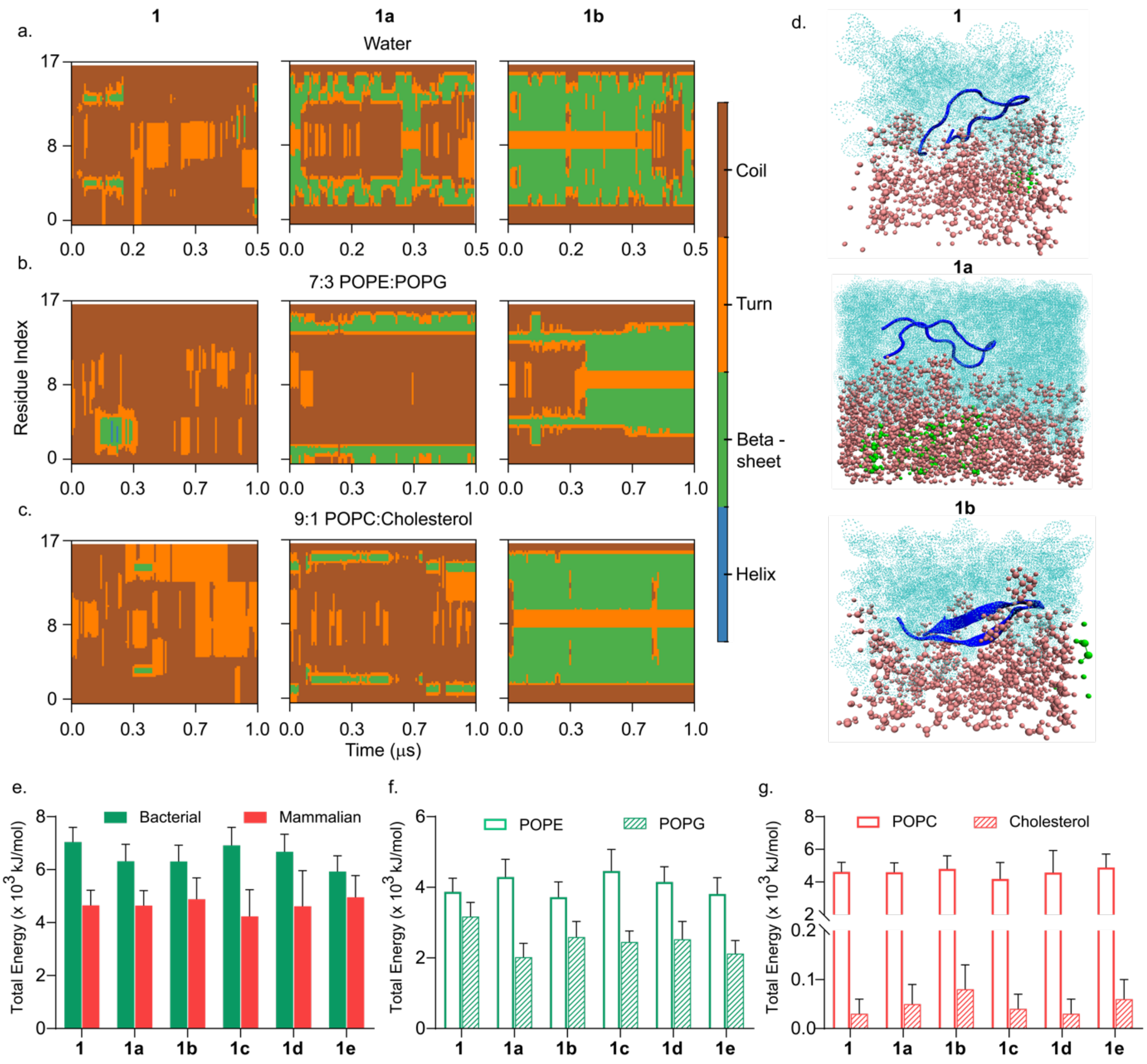

Figure S6. (a) Secondary structure heatmaps for disulfide bridged peptides **1, 1a,** and **1b,** a) in water returned a β-sheet content of 4.11% in **1**, 29.46% in **1a**, and 57.64% in **1b**; b) in liposome mimicking the bacterial membrane (7:3 POPE:POPG) shows a progressive increase from **1** (0.05%) to **1a** (19.48%) and to **1b** (38.46%). This supports the experimental observation that as rigidity increases in the order **1** < **1a** < **1b**, binding to bacterial liposomes becomes more efficient. c) In liposome mimicking the mammalian membrane (9:1 POPC:Cholesterol) the β-sheet content of **1** (1.51%) and **1a** (9.44%) and SASA values in the human membrane (Table S4) aligns with the experimental finding that they have similar interactions with mammalian liposomes. In contrast, **1b** shows a much higher β-sheet content (66.94%) and significantly lower SASA in the human membrane (10.62 nm^2). d) Simulation snapshots showing the interaction of the peptides with the mammalian membrane. The peptide is shown in blue, water in cyan, POPC lipids in pink, and cholesterol in green. (e) Total peptide-lipid interaction energies for all peptide variants against bacterial and mammalian membrane models. The plot illustrates the preferential binding of all peptides to the bacterial membrane. (f) Total peptide-lipid interaction energies with individual components of the bacterial membrane, highlighting the strong binding to both POPE and POPG lipids. (g) Total protein-lipid interaction energies with individual components of the mammalian membrane, showing the dominant contribution of POPC over Cholesterol.

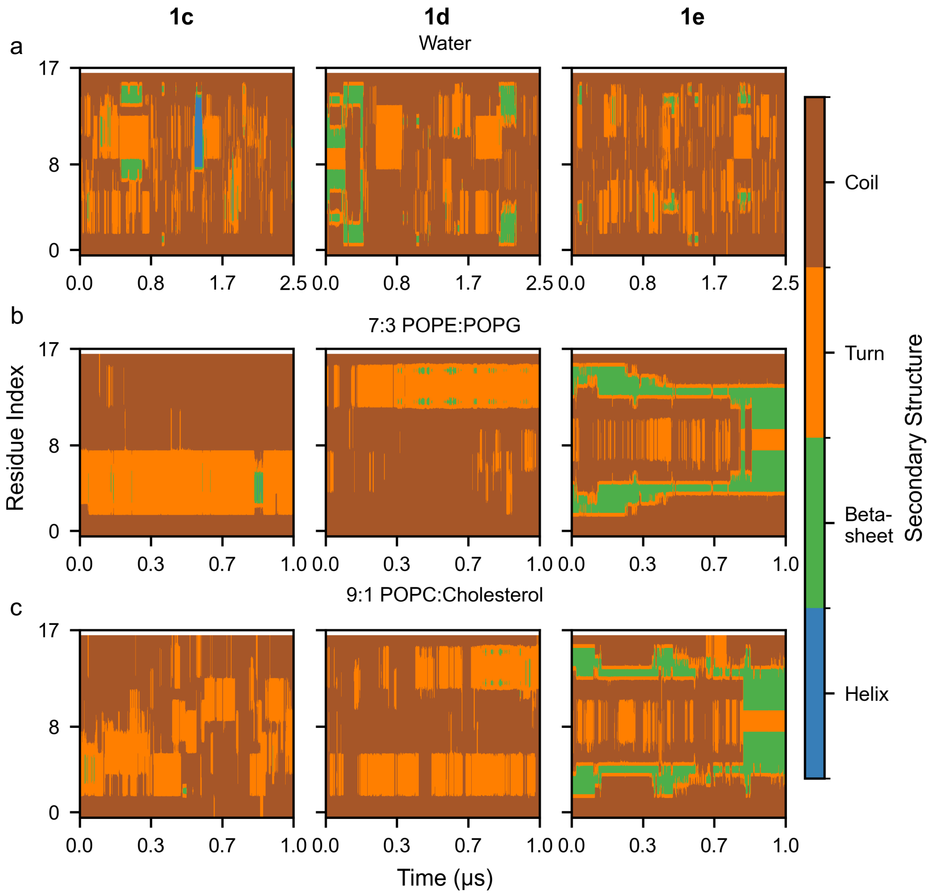

Figure S7. (a) Secondary structure heatmaps for threonine-substituted peptides **1c, 1d,** and **1e,** a) in water, b) in liposome mimicking the bacterial membrane (7:3 POPE:POPG), c) in liposome mimicking the mammalian membrane (9:1 POPC:Cholesterol).

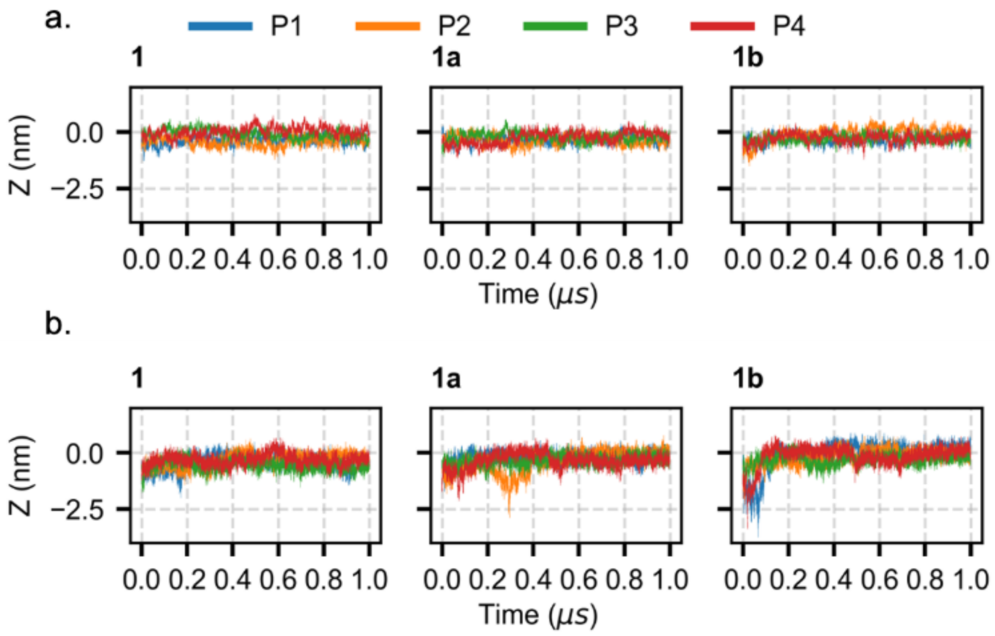

Figure S8. Average Z-Coordinate (nm) of peptide center of mass relative to membrane center, showing interaction with a) bacterial and b) mammalian membranes. This suggests that the peptides are on the membrane surface till 1μs, as confirmed by the consistent center of mass (COM) distances.

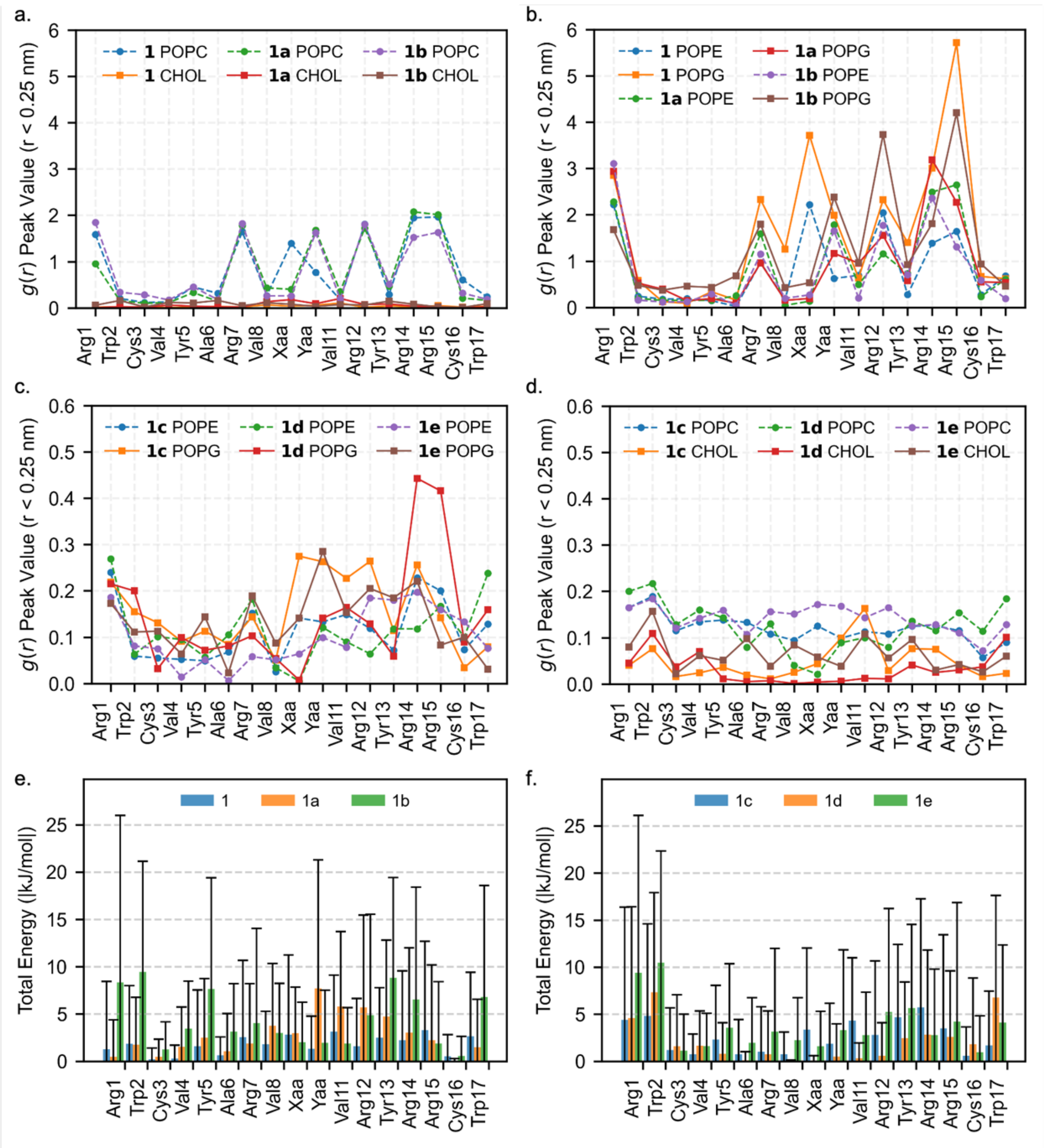

Figure S9. Residue-level interactions with membrane lipids. (a) Radial distribution function *g(r)* peak values for disulphide-containing peptides in the bacterial membrane, showing strong electrostatic interactions. (b) *g(r)* peak values for disulphide-containing peptides in the mammalian membrane. (c) *g(r)* peak values for threonine-substituted peptides in the bacterial membrane. (d) *g(r)* peak values for threonine-substituted peptides in the mammalian membrane. Total interaction energy magnitude between individual peptide residues and cholesterol (CHOL). (e) Residue-cholesterol interaction energies for disulphide-containing peptides **1**, **1a**, and **1b**. (f) Residue-cholesterol interaction energies for threonine-substituted peptides **1c**, **1d**, and **1e**. The plots show contributions from Lennard-Jones and Coulomb interactions.

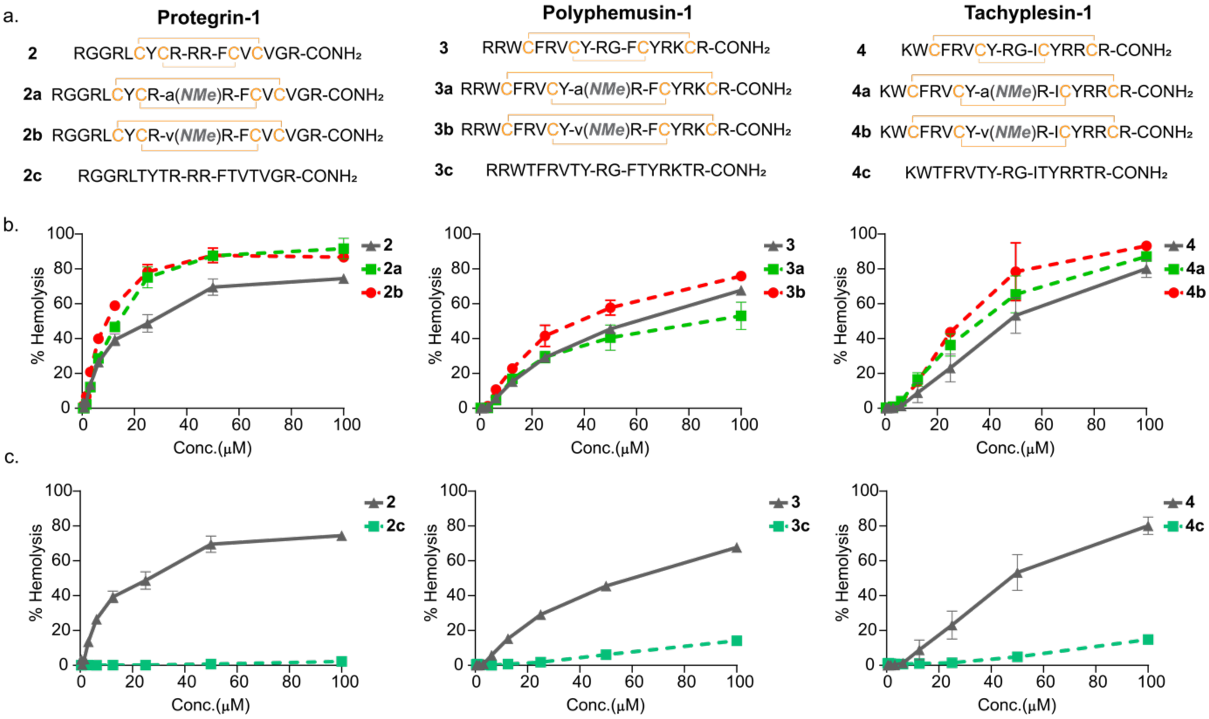

Figure S10: (a) Sequences of Protegrin-1(**2**), Polyphemusin-1(**3**) and Tachyplesin-1(**4**) wild-type and their cyclic variants**.** (b) Hemolysis data (n=3) comparison of wild-type Protegrin-1, Polyphemusin-1 and Tachyplesin-1 and their respective turn engineered cyclic variants. (c) Hemolysis data (n=3) comparison of wild-type Protegrin-1, Polyphemusin-1 and Tachyplesin-1 and their respective linearized variants.

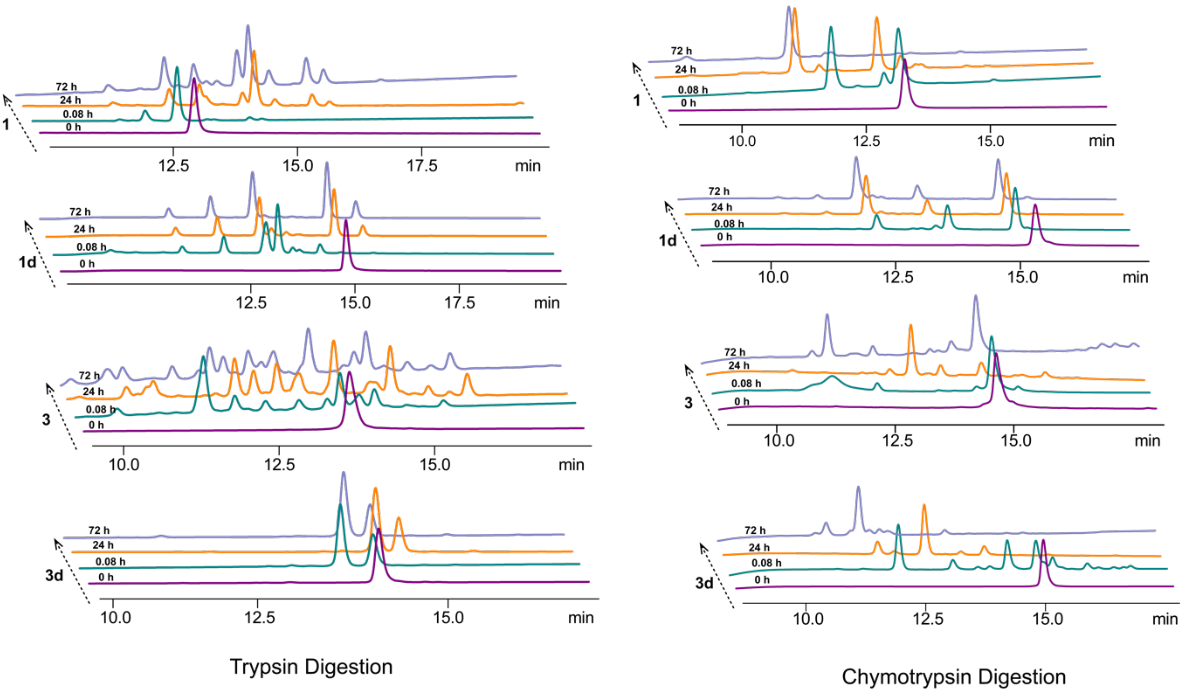

Figure S11: Proteolytic degradation profiles of **1**, **1d**, **3** and **3d.** The four overlaid chromatograms represent four different time-points: 0 h, 5 min, 24 h and 72 h. The presence or absence of the native peptide was monitored using MALDI-TOF.

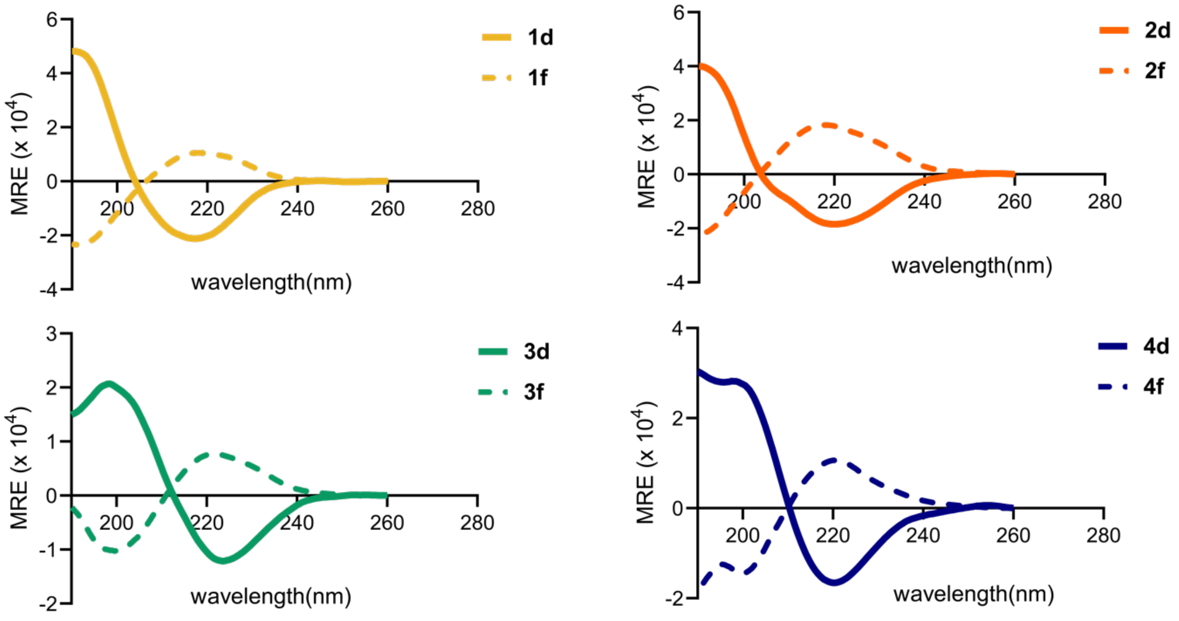

Figure S12: CD spectra of **1d**, **2d**, **3d**, **4d** (in solid line) and their enantiomeric variants, **1f**, **2f**, **3f**, **4f** (dotted lines) in 10 mM SDS.

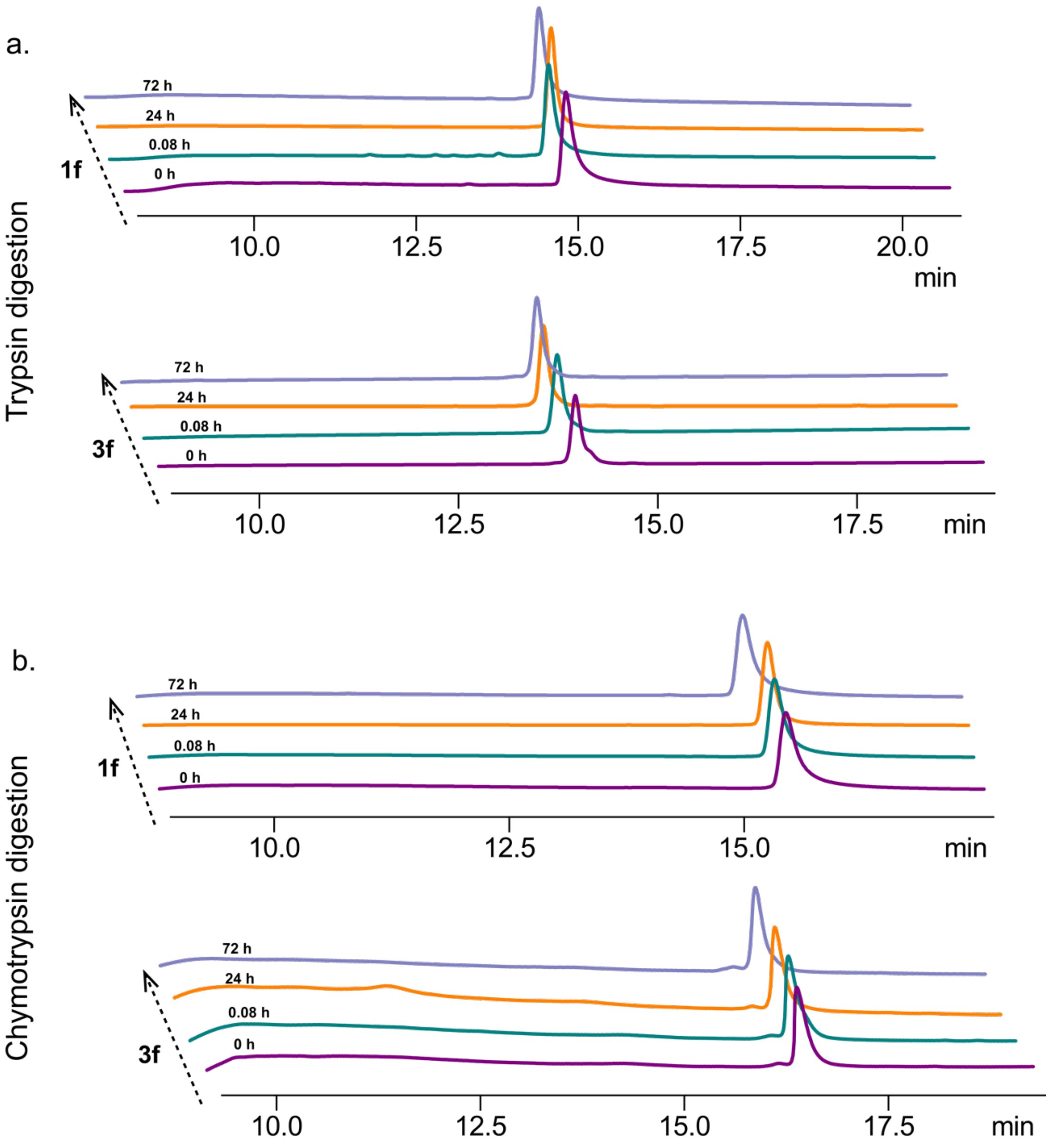

Figure S13: Proteolytic degradation profiles of **1f** and **3f.** The four overlaid chromatograms represent four different time-points: 0 h, 5 min, 24 h and 72 h. The presence or absence of the native peptide was monitored using MALDI-TOF.

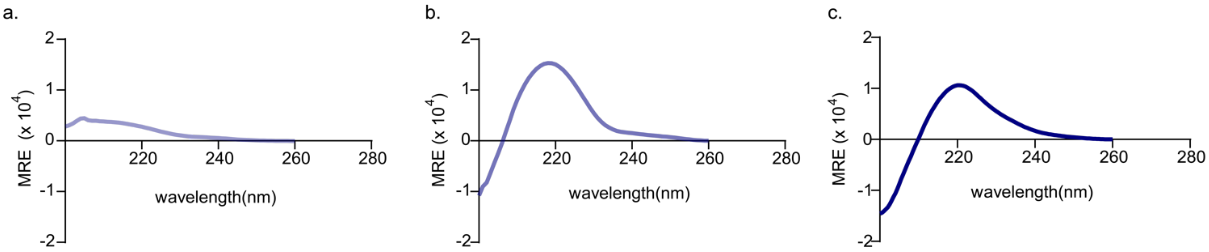

Figure S14: CD signature of **4f** in: a. 1X PBS, b. 50 µM *E. coli* LPS micelles, c. 10 mM SDS micelles.

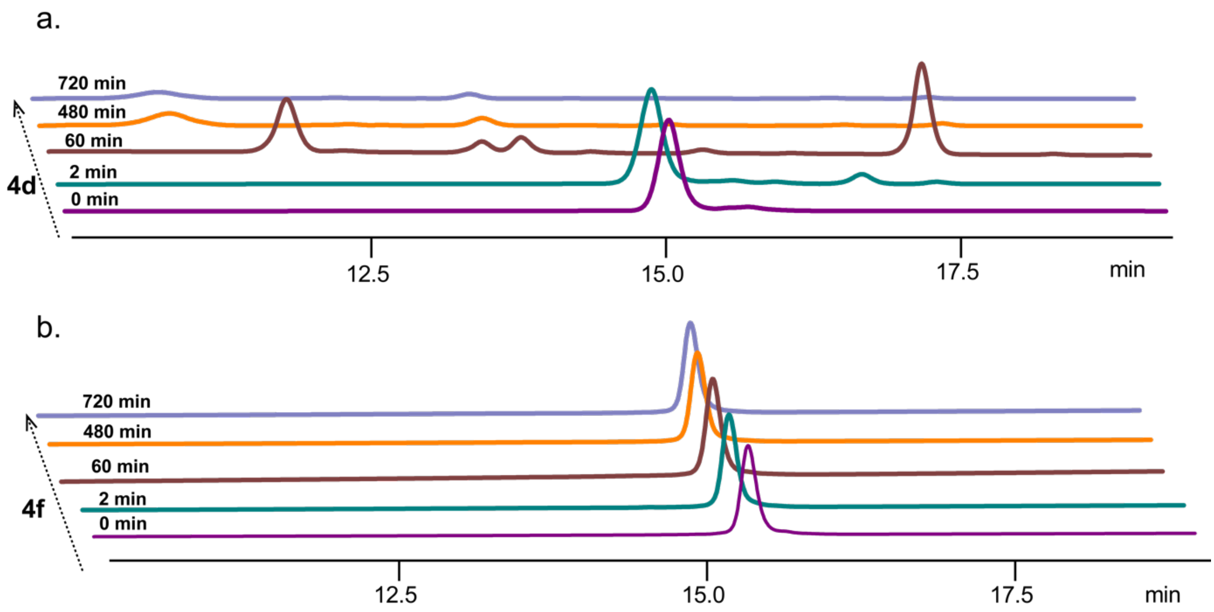

Figure S15: Proteolytic degradation profiles of a. **4d** and b. **4f** after incubation with Proteinase K. The five overlaid chromatograms represent four different time-points: 0 min, 2 min, 60 min, 480 min and 720 min. The identity of the peptide at different time points were monitored using MALDI-TOF.

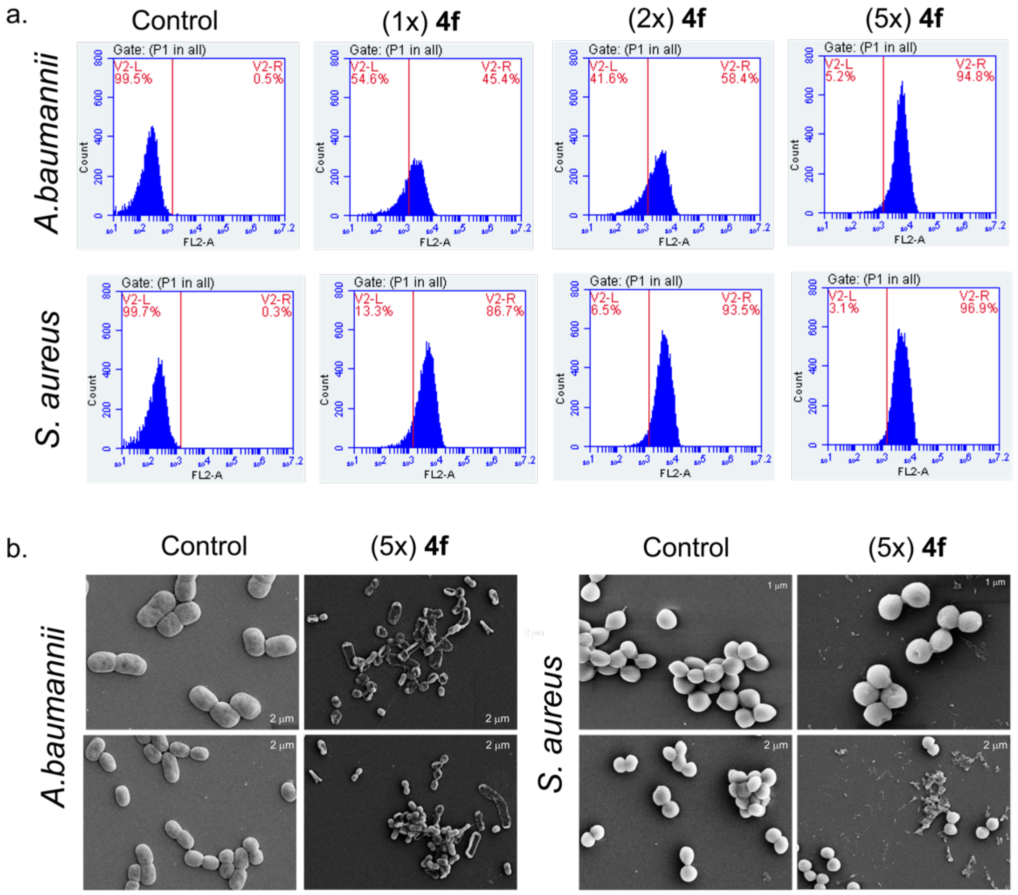

Figure S16: a. PI uptake by *A. baumannii* and *S. aureus*, when treated with different concentrations of **4f** for 10 min, monitored using flow-cytometry. b. SEM images of untreated control cells and 5 times of MIC of **4f** treated cells of *A. baumannii* and *S. aureus* after 30 min of incubation.

**4**	**4f**	Peptide name
2.7 x 10^8 ± 7.2 x 10^7	1 x 10^9 ± 4.1 x 10^8	K_{A1}(M^{-1})
5.1 x 10^5 ± 2.1 x 10^5	3.8 x 10^5 ± 1 x 10^5	K_{A2}(M^{-1})
0.78 ± 0.052	0.7 ± 0.039	n1
0.99 ± 0.064	0.9 ± 0.052	n2
-10.97 ± 0.52	-14.32 ± 0.395	ΔH1 (kJ/mol)
-16.26 ± 0.99	-19.60 ± 0.807	ΔH2 (kJ/mol)
3.7 x 10^9	1 x 10^9	K_{D1}(M)
1.9 x 10^6	2.6 x 10^6	K_{D2}(M)
1.25 x 10^2	1.24 x 10^2	ΔS1 (J/mol.K)
55	41	ΔS2 (J/mol.K)
-49.92	-51.27	ΔG1 (kJ/mol)
-32.65	-31.82	ΔG2 (kJ/mol)

Figure S17: Thermodynamic parameters of **4** and **4f**, obtained from ITC.

	ec	***pa***	***ab***	***kp***	***sa***
**4**	0.1	0.8	0.6	0.8	0.8
**4f**	0.4	1.2	1.6	1.6	0.8

Figure S18: MIC of **4** and **4f**, against non-resistant class of ESKAPE pathogens. (*ec* - *Escherichia coli, pa* - *Pseudomonas aeruginosa, ab - Acinetobacter baummannii, kp* - *Klebsiella pneumoniae and sa - Staphylococcus aureus)*.

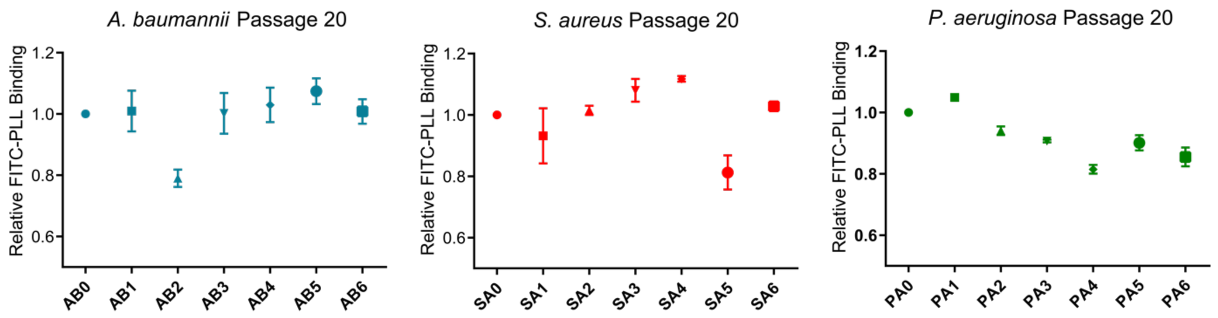

Figure S19: Surface charge measurement of the six-independent laboratory evolved resistant strains of *A. baumannii*, *S. aureus* and *P. aeruginosa* against **4f**. The measurement was performed on strains, generated after 20 passages.

a.

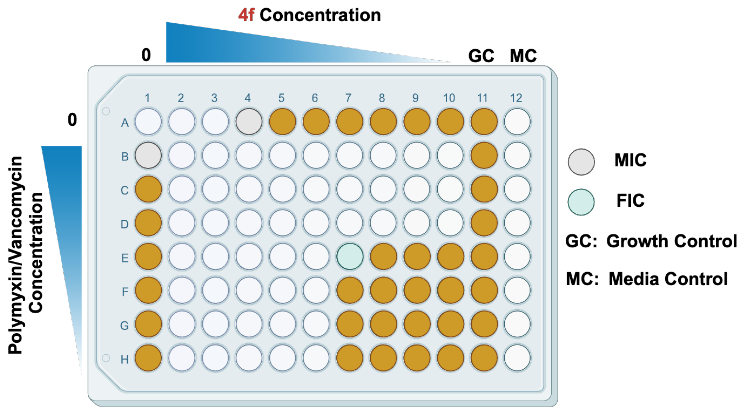

b.

	Polymyxin-B resistant *A. baumannii*	Polymyxin-B resistant *P. aeruginosa*	Vancomycin-resistant *S. aureus*
**Median FIC.** (n = 12)	1.25	1.25	1.03

Figure S20: a. Checkerboard assay set-up with sample result output. Culture growth is shown by yellow circles. b. Table highlighting the median FIC, calculated from the assay. Peptide concentrations used for the assay are, **4f**: 12.5 μM-0.2 μM; polymyxin B: 3100 μg/ml-48.5 μg/ml and vancomycin: 22.1 μg/ml-0.34 μg/ml.

Acinetobacter baumannii

Strain	Antibiotic Resistance	**4f** MIC(μM)
RPTC 1	NA,PEN,CIP,NOR,AMP,K,TE,MTZ,GEN,TMP	2
RPTC 2	NA,PEN,CIP,NOR,AMP,K,TE,MTZ,GEN,TMP	4
RPTC 3	NA,PEN,CIP,NOR,AMP,K,TE,MTZ,GEN,TMP	2
RPTC 4	NA,PEN,CIP,NOR,AMP,K,TE,MTZ,GEN	4
RPTC 5	NA,PEN,CIP,NOR,AMP,K,TE,MTZ,GEN,TMP	4
RPTC 6	MEM,AMK,STR	4
RPTC 7	NA,PEN,CIP,NOR,AMP,K,TE,MTZ,GEN,TMP	4
RPTC 8	NA,PEN,CIP,NOR,AMP,K,TE,MTZ,GEN,TMP	4
RPTC 11	NA,PEN,CIP,NOR,AMP,K,MTZ,GEN,TMP	4
RPTC 14	MEM,AK,STR	4
RPTC 15	AMK,STR	4
RPTC 21	NA,PEN,CIP,NOR,AMP,K,MTZ,GEN,TMP	1
RPTC 22	NA,PEN,CIP,NOR,AMP,K,MTZ,GEN,TMP	1
RPTC 23	MEM,AMK,STR	1
RPTC 24	MEM,AMK,STR,RIF	1
RPTC 25	NA,PEN,CIP,NOR,AMP,K,MTZ,GEN,TMP	0.5
RPTC 59	AK,A/S,CIP,AT,CFM,IPM,CEC,NA,COT,K,TOB	0.5
RPTC 61	AK,A/S,CIP,AT,CFM,IPM,CEC,NA	0.5
RPTC 64	AK,A/S,CIP,AT,CFM,IPM,CEC,NA,K,TOB	1

Staphylococcus aureus

Strain	Antibiotic Resistance	**4f** MIC(μM)
RPTC 6	PEN,AMC,AMP,NA,RIF,PB,FA,OXA	2
RPTC 7	PEN,AMC,K,AMP,NA,CIP,ER,PB	1
RPTC 8	PEN,AMC,K,AMP,NA,CIP,ER,PB	1
RPTC 9	PEN,AMC,K,AMP,NA,CIP,ER,PB,AMI,FA,OXA	0.25
RPTC 10	PEN,AMC,K,AMP,NA,CIP,ER,PB	0.25
RPTC 11	PEN,COT,AMC,K,AMP,NA,CIP,ER,PB,FA	1
RPTC 12	PEN,COT,AMC,K,AMP,NA,RIF,CIP,ER,PB,OXA	0.25
RPTC 13	PEN,COT,AMC,K,AMP,NA,RIF,CIP,ER,PB,OXA	0.25
RPTC 14	PEN,AMC,K,AMP,NA,CIP,ER,PB	1
RPTC 15	PEN,AMC,K,AMP,NA,CIP,ER,PB	1
RPTC 16	PEN,AMC,K,AMP,NA,CIP,ER,PB,FA	1
RPTC 60	AT,TOB,A/S,K,MET,VAN	0.5

NA:Nalidixic acid; PEN:Penicillin; CIP:Ciprofloxacin; NOR/NX:Norfloxacin; AMP:Ampicillin; K:Kanamycin; TE:Tetracycline; MTZ:Metronidazole; GEN:Gentamycin; TMP:Trimethoprim; MEM:Meropenem; AK/AMK:Amikacin; STR:Streptomycin; RIF:Rifampicin; A/S:Amoxycillin/Sulbactam; AT:Aztreonam; CFM:Cefixime; IPM:Imipenem; CEC:Cefotaxime/Clavulanic acid; COT:Co-trimoxazole; TOB: Tobramycin; ER:Erythromycin; PB:Polymyxin B; OXA:Oxacillin; FA:Fusidic acid; MET:Methicillin; VAN:Vancomycin

Figure S21: List of MDR clinical isolates of *A. baumannii* and *S. aureus*.

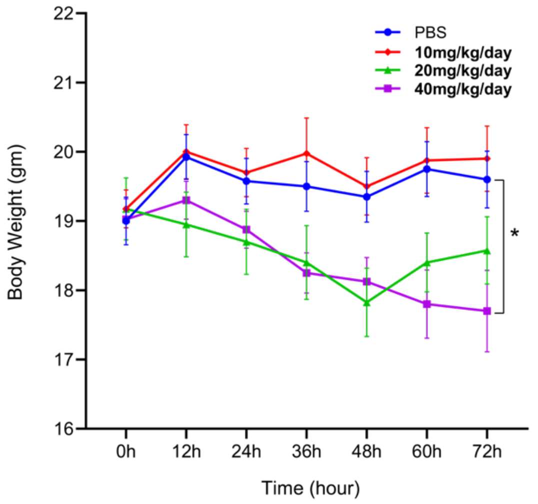

Figure S22: Toxicity studies using different doses **4f**, monitored in terms of change in body weight.

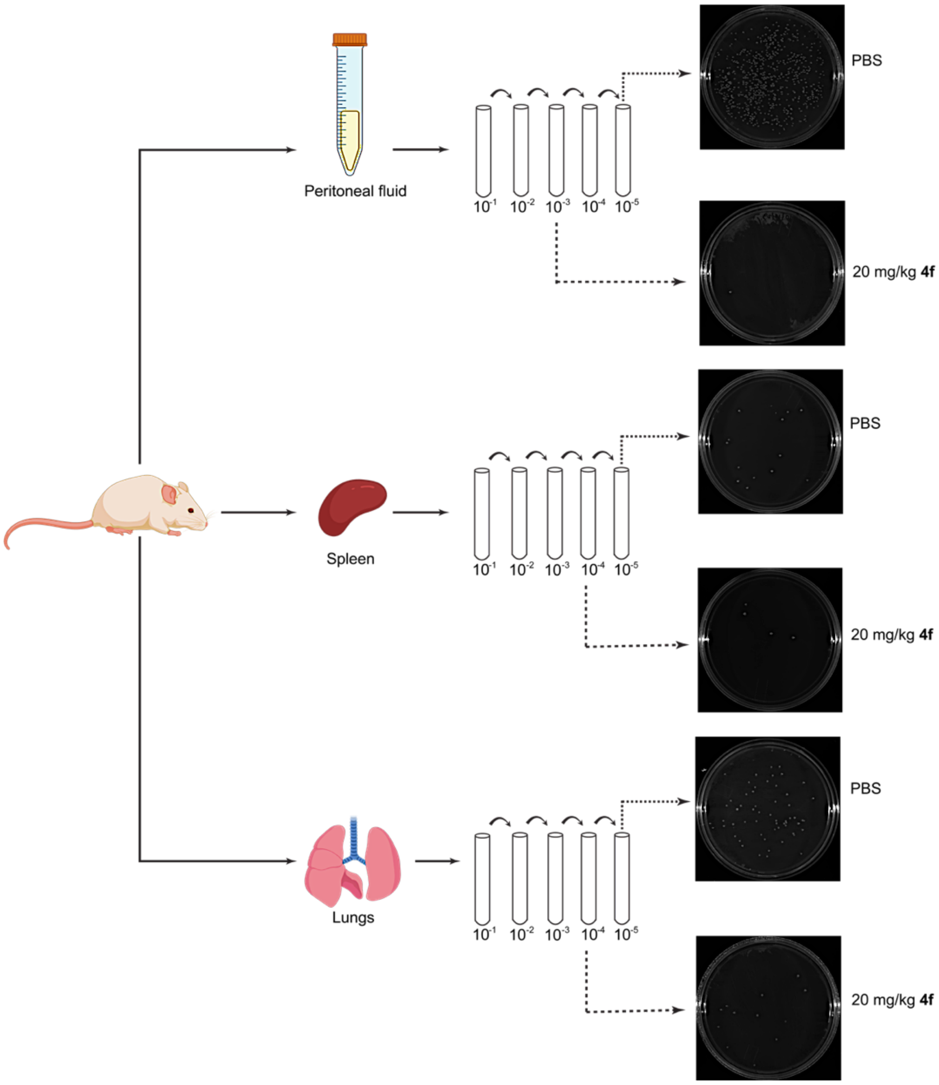

Figure S23: Bacterial load determination in mice peritoneal fluid, spleen and lungs, with or without treatment of **4f**.

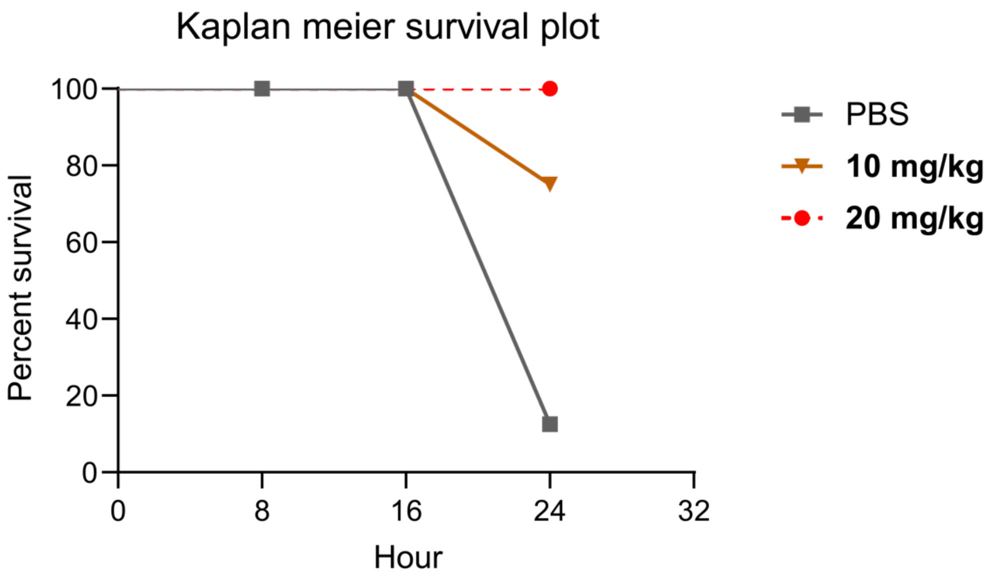

Figure S24: Survival analysis for n= 8 mice per group 24 hours post treatment with different doses of **4f**.

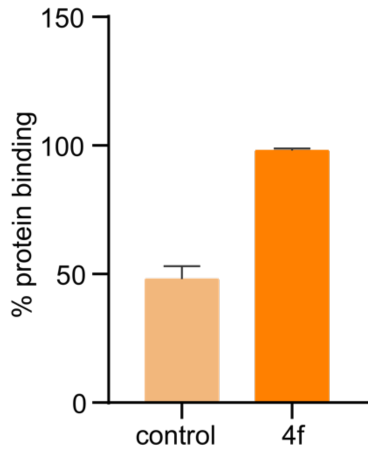

Figure S25: Rapid Equilibrium dialysis data of a cyclic peptide control (*cyclo*(l-L-L-p-Y-L)[1] and **4f**.

Table S1. Average secondary structure content from molecular dynamics simulations of cysteine-based and linear threonine-substituted peptides in water.

System	Helix (%)	β-sheet (%)	Turn (%)	Coil (%)
**1**	0.25 ± 2.10	4.11 ± 8.17	17.81 ± 15.84	77.82 ± 16.35
**1a**	0.00 ± 0.00	29.46 ± 16.14	11.57 ± 12.36	58.97 ± 20.89
**1b**	0.00 ± 0.00	57.64 ± 17.54	12.14 ± 6.03	30.22 ± 18.47
**1c**	2.37 ± 8.58	2.74 ± 7.08	25.62 ± 19.46	69.28 ± 20.96
**1d**	0.08 ± 1.19	9.95 ± 17.34	20.73 ± 17.70	69.24 ± 20.87
**1e**	0.24 ± 2.07	2.78 ± 6.63	22.94 ± 18.01	74.03 ± 18.91

Table S2. Average secondary structure content from molecular dynamics simulations of cysteine-based and linear threonine-substituted peptides on bacterial membrane.

System	Helix (%)	β-sheet (%)	Turn (%)	Coil (%)
**1**	1.67 ± 5.34	0.05 ± 0.90	17.83 ± 16.87	80.45 ± 16.74
**1a**	0.00 ± 0.00	19.48 ± 6.02	3.32 ± 8.28	77.21 ± 10.11
**1b**	0.00 ± 0.00	38.46 ± 18.80	11.39 ± 8.19	50.15 ± 20.77
**1c**	0.64 ± 3.41	0.00 ± 0.24	36.27 ± 10.08	63.09 ± 8.28
**1d**	0.00 ± 0.00	2.54 ± 4.84	24.09 ± 13.26	73.37 ± 12.68
**1e**	0.00 ± 0.00	25.56 ± 13.90	10.97 ± 11.19	63.47 ± 17.35

Table S3. Average secondary structure content from molecular dynamics simulations of cysteine-based and linear threonine-substituted peptides on human membrane.

System	Helix (%)	β-sheet (%)	Turn (%)	Coil (%)
**1**	0.03 ± 0.66	1.51 ± 4.52	35.71 ± 22.14	62.76 ± 22.18
**1a**	0.00 ± 0.00	9.44 ± 5.99	15.21 ± 16.06	75.34 ± 18.30
**1b**	0.00 ± 0.00	66.94 ± 9.87	12.25 ± 3.59	20.81 ± 9.39
**1c**	0.22 ± 1.95	0.28 ± 1.36	31.96 ± 17.83	67.54 ± 17.54
**1d**	0.11 ± 1.37	0.91 ± 3.15	31.49 ± 17.08	67.49 ± 17.34
**1e**	0.00 ± 0.00	24.56 ± 16.36	14.30 ± 12.50	61.13 ± 19.75

Table S4. Average SASA of peptide systems in different environments in nm^2.

System	Aqueous medium (nm^2)	Bacterial Membrane (nm^2)	Human Membrane (nm^2)
**1**	24.04 ± 1.34	10.55 ± 2.12	12.19 ± 2.69
**1a**	22.95 ± 0.99	11.87 ± 2.12	12.20 ± 3.16
**1b**	22.38 ± 0.90	11.35 ± 2.26	10.62 ± 3.59
**1c**	27.35 ± 0.96	12.63 ± 1.96	15.64 ± 3.44
**1d**	25.12 ± 0.99	12.86 ± 2.10	15.04 ± 4.48
**1e**	25.99 ± 0.22	12.40 ± 1.66	12.37 ± 3.37

Table S5. Summary of total protein-lipid interaction energies (kJ/mol) for all peptide systems.

System	Bacterial Interaction (kJ/mol)	Human Interaction (kJ/mol)	Difference (kJ/mol)
**1**	7040.83 ± 558.79	4649.49 ± 575.68	2391.34
**1a**	6318.40 ± 635.32	4642.31 ± 567.58	1676.10
**1b**	6312.04 ± 609.98	4882.05 ± 805.42	1429.99
**1c**	6913.99 ± 683.09	4237.98 ± 1002.95	2676.01
**1d**	6676.51 ± 660.10	4615.77 ± 1349.18	2060.74
**1e**	5926.70 ± 594.24	4957.92 ± 818.66	968.78

HPLC Chromatograms of all peptides

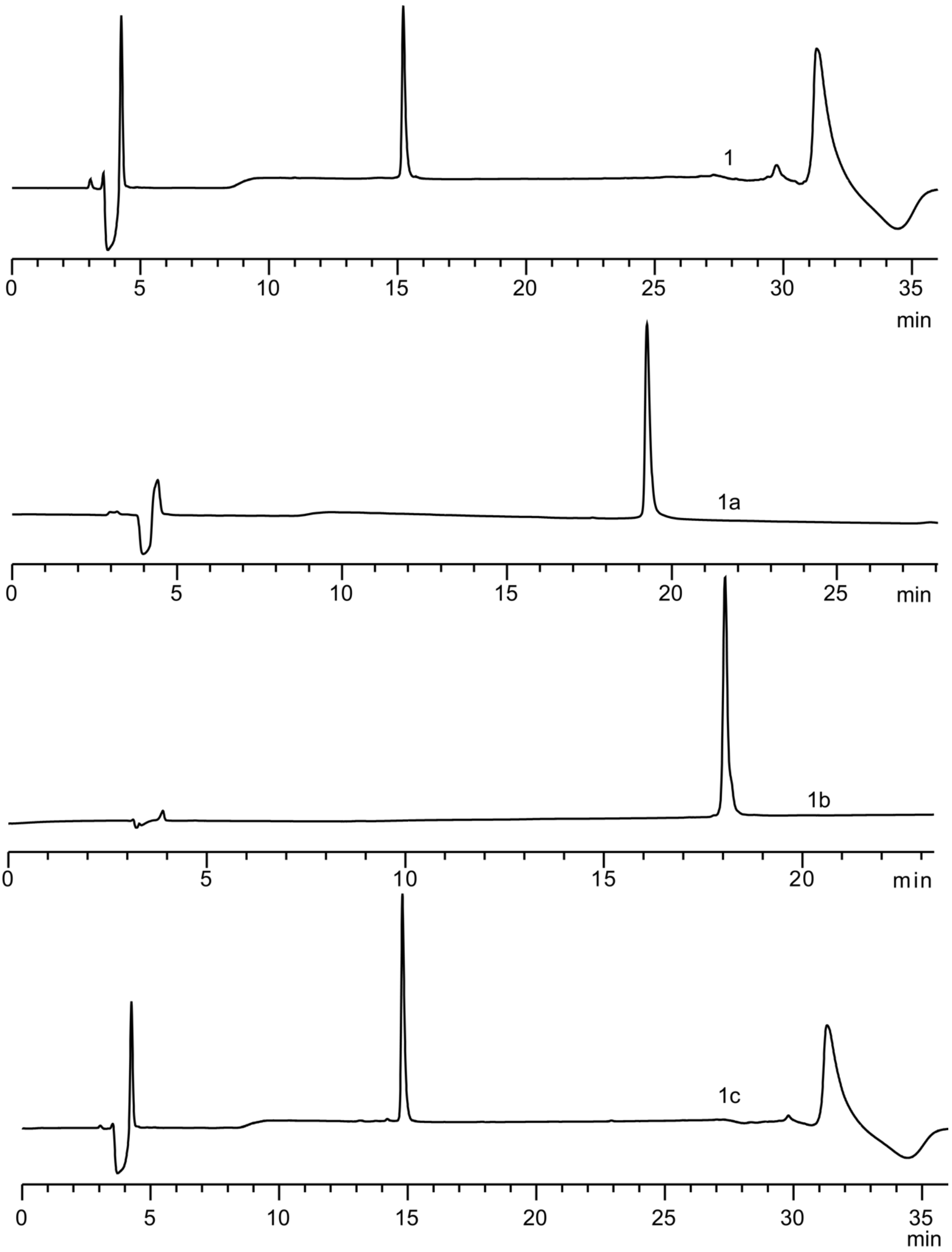

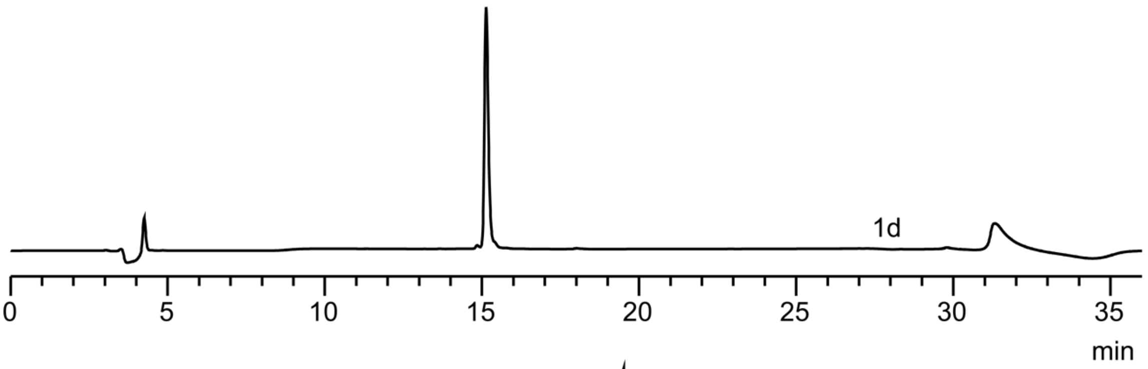
1d
0
5
10
15
20
25
30
35
min

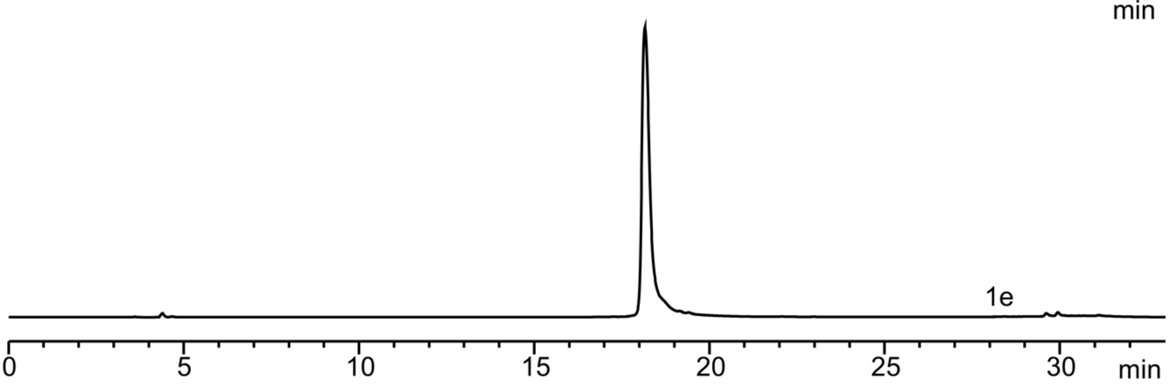
1e
0
5
10
15
20
25
30
min

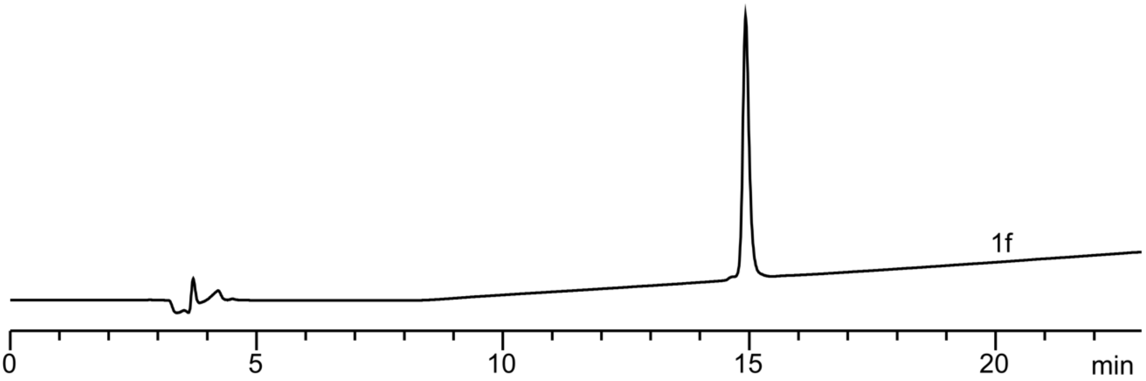
1f
0
5
10
15
20
min

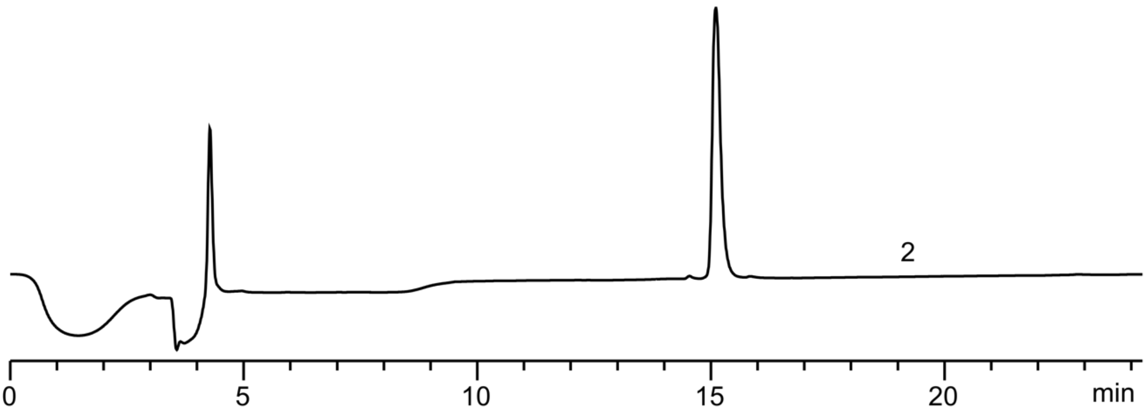
2
0
5
10
15
20
min

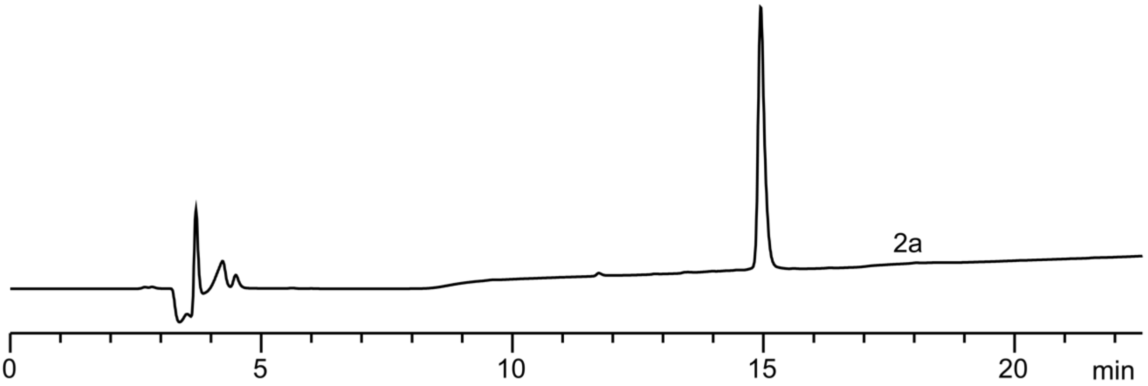
2a
0
5
10
15
20
min

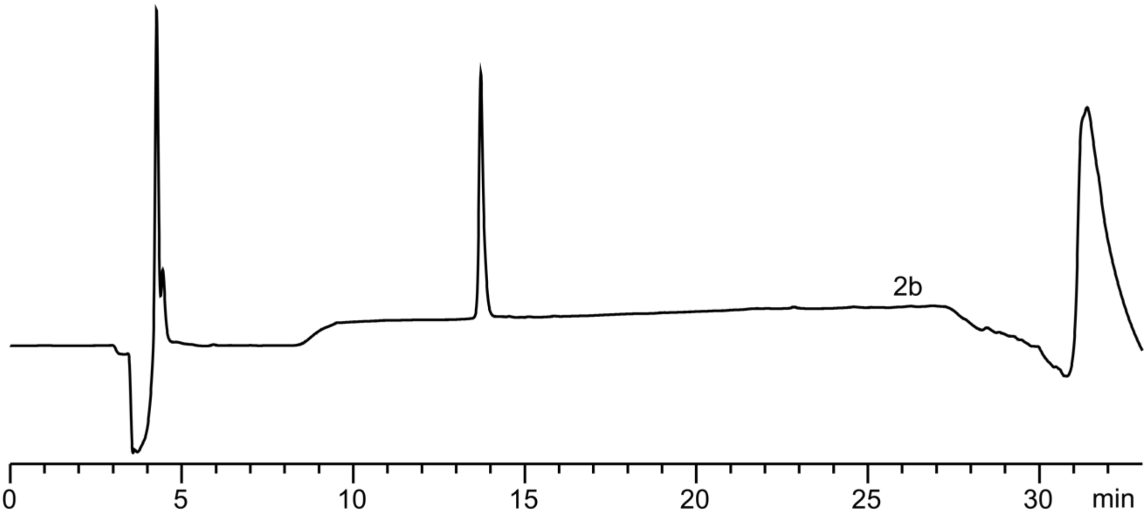
2b
0
5
10
15
20
25
30
min

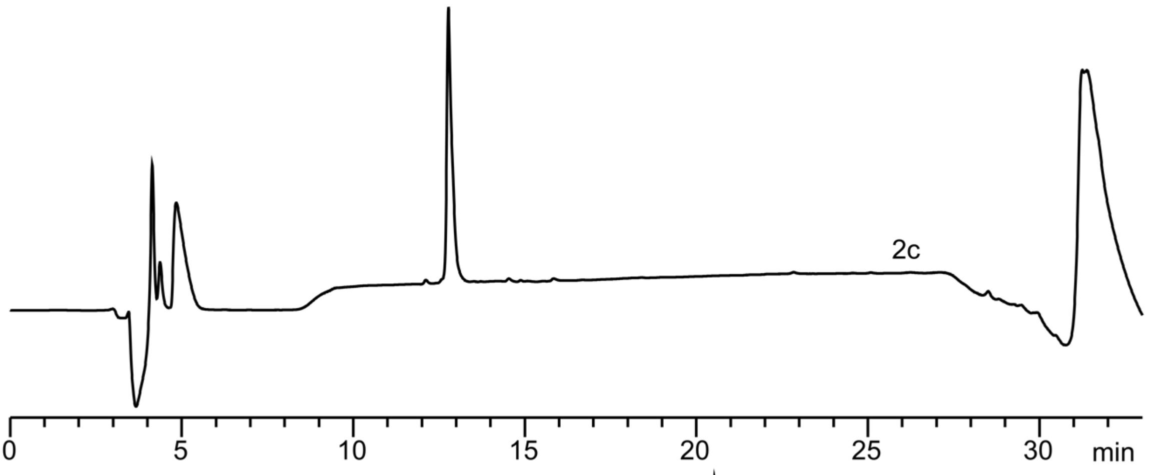
2c
0
5
10
15
20
25
30
min

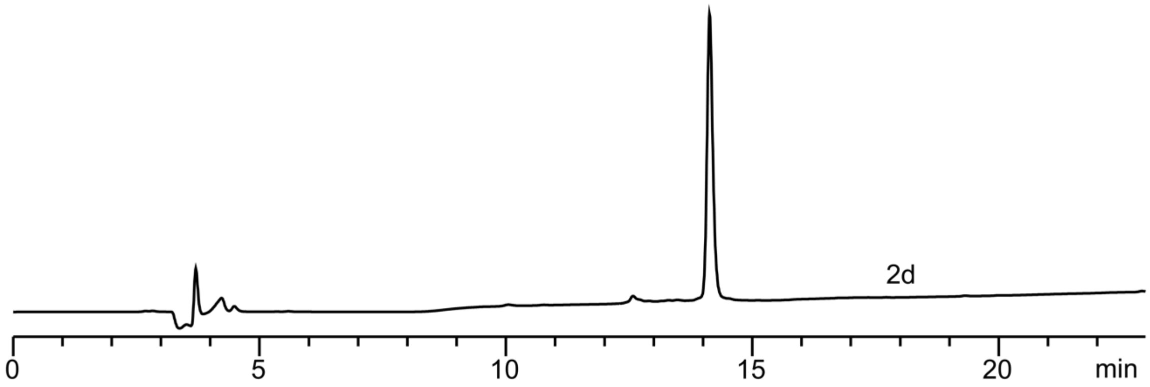
2d
0
5
10
15
20
min

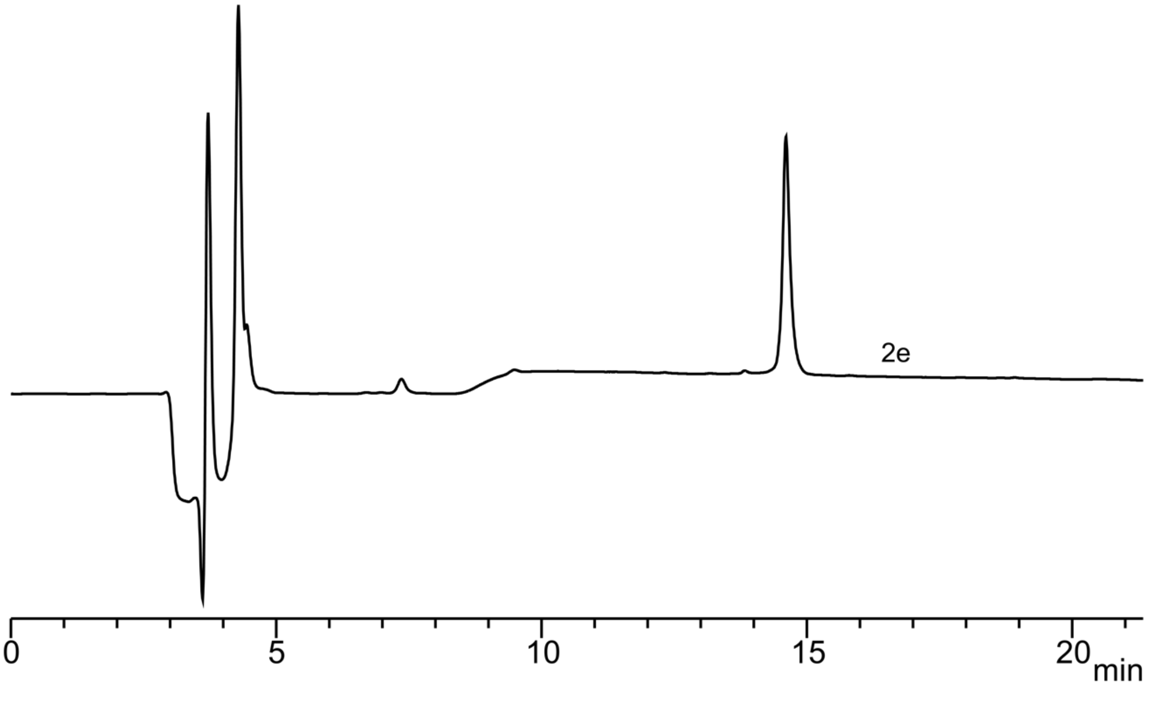
2e
0
5
10
15
20
min

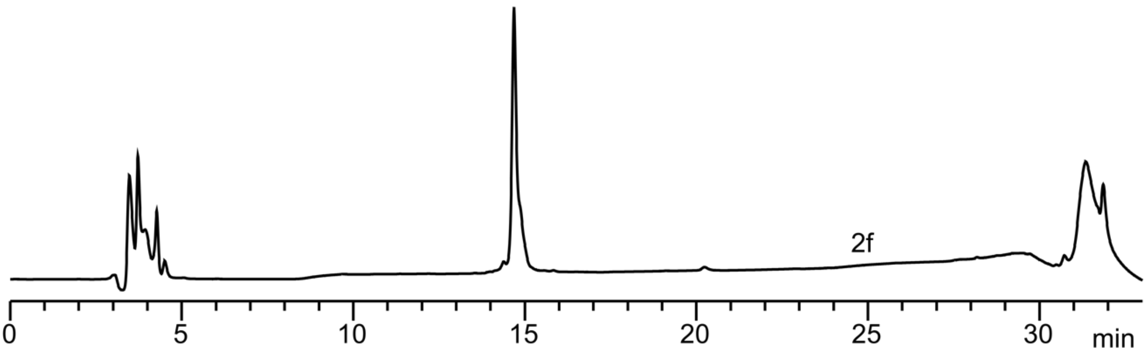
2f
0
5
10
15
20
25
30
min

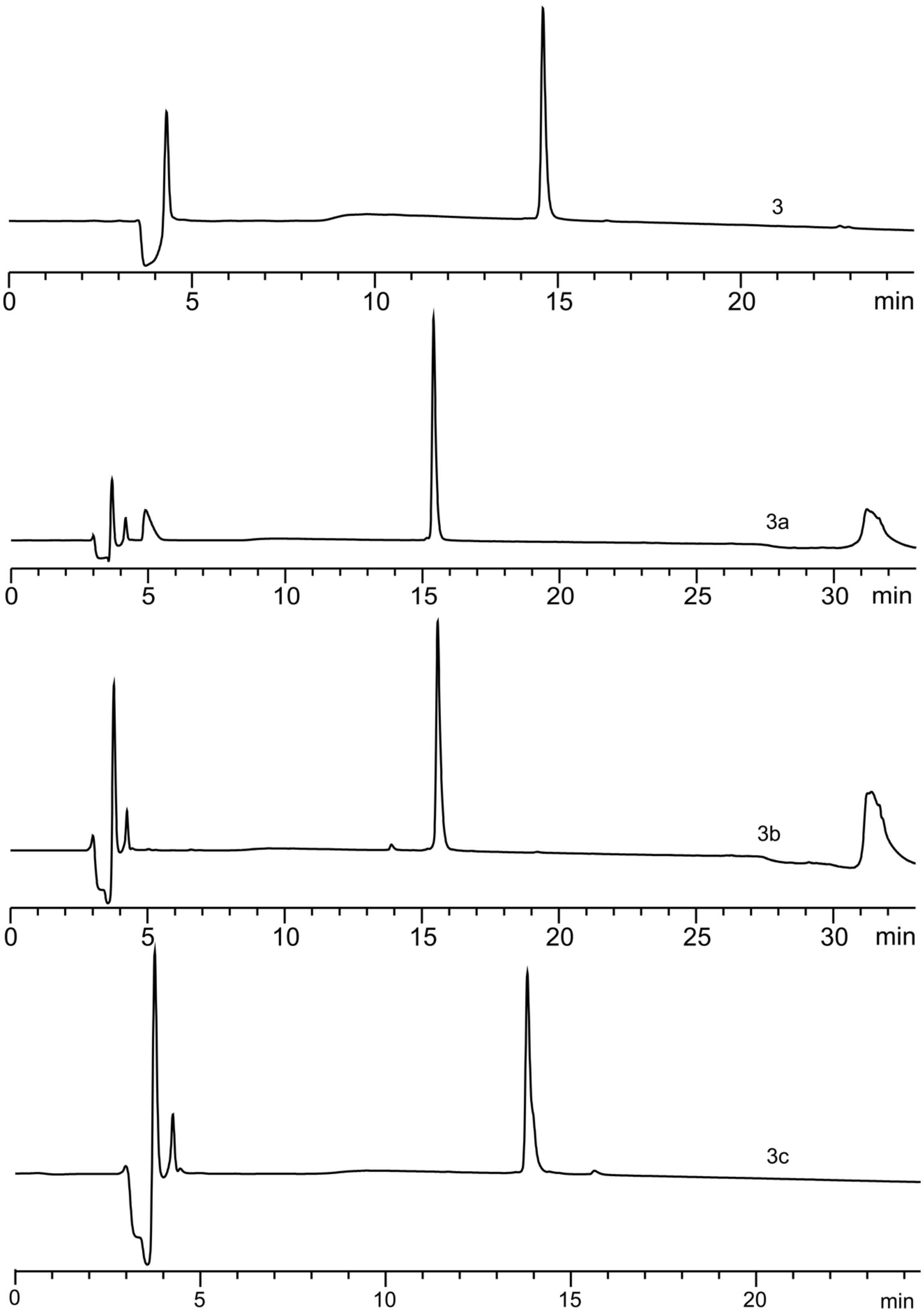
3
0 5 10 15 20 min
3a
0 5 10 15 20 25 30 min
3b
0 5 10 15 20 25 30 min
3c
0 5 10 15 20 min

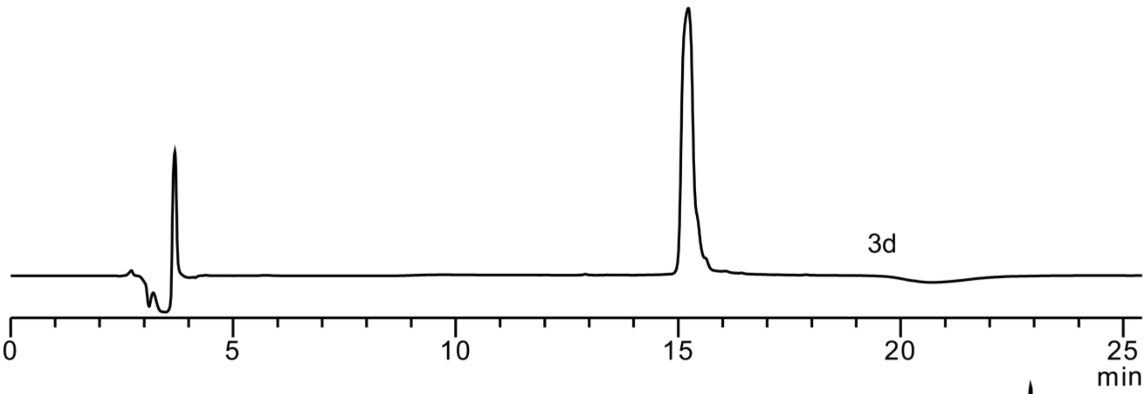
3d
0
5
10
15
20
25
min

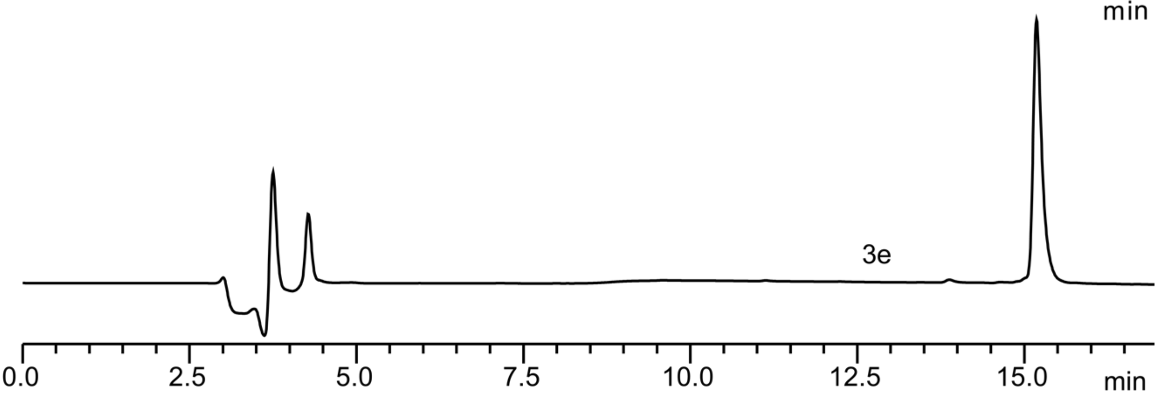
3e
0.0
2.5
5.0
7.5
10.0
12.5
15.0
min

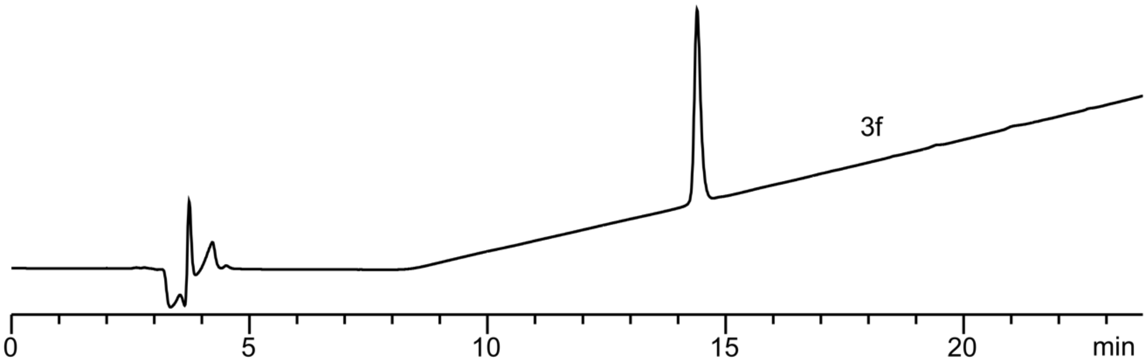
3f
0
5
10
15
20
min

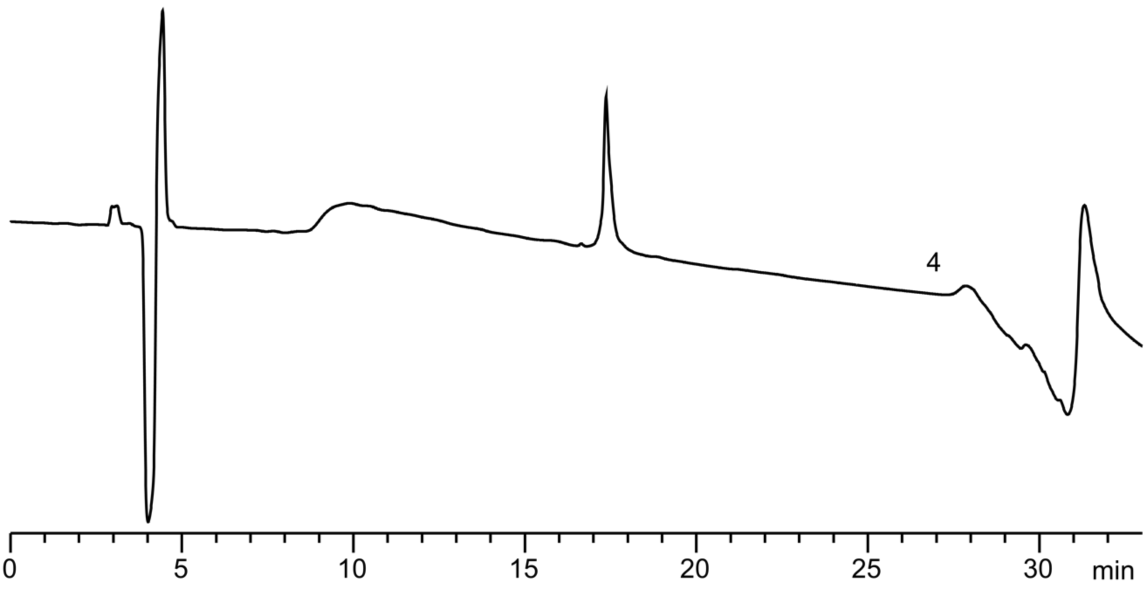
4
0
5
10
15
20
25
30
min

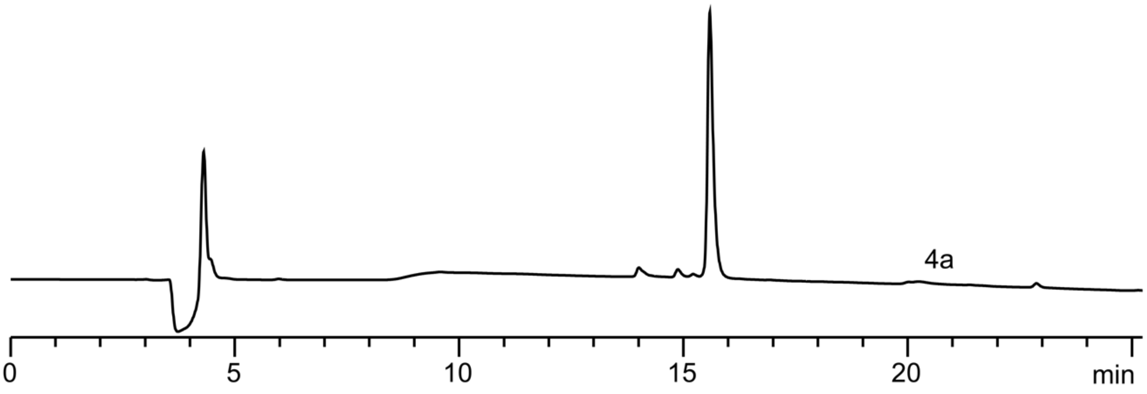
4a
0
5
10
15
20
min

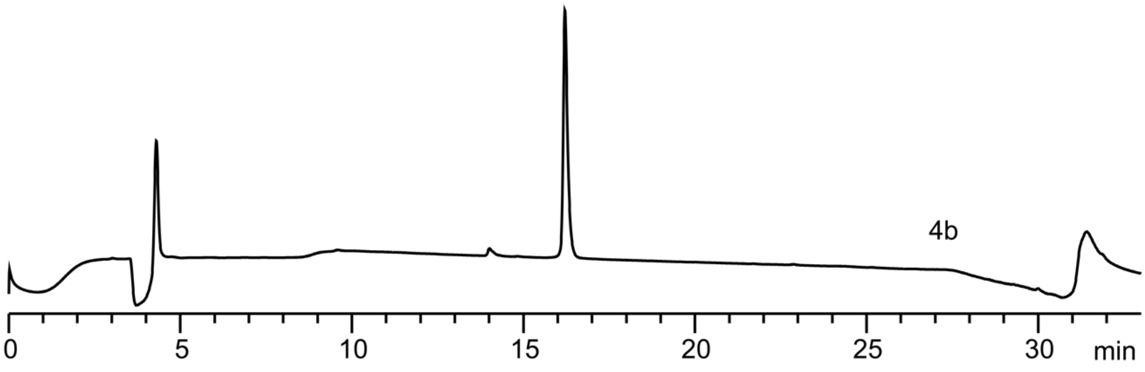
4b
0
5
10
15
20
25
30
min

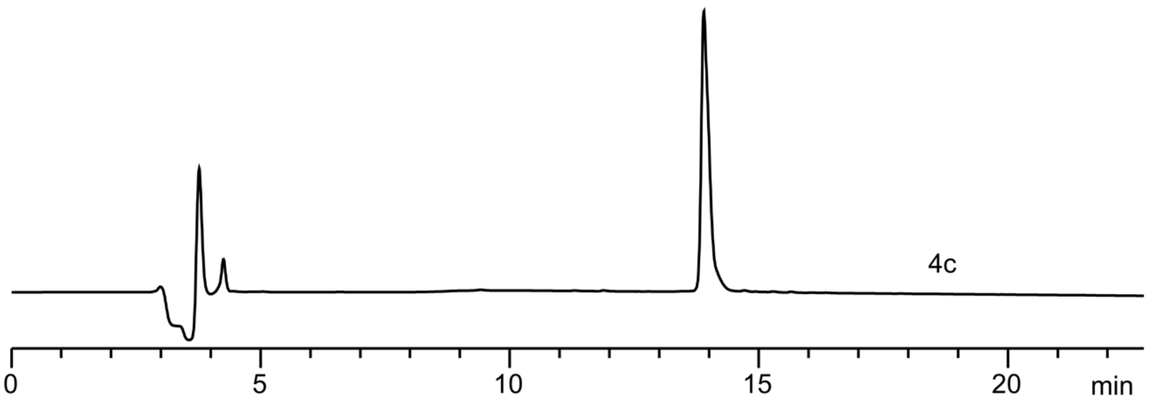
4c
0
5
10
15
20
min

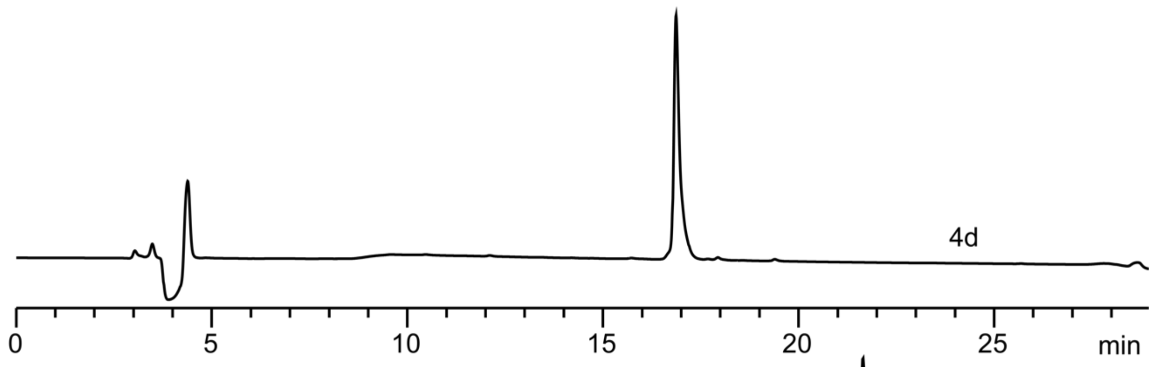
4d
0
5
10
15
20
25
min

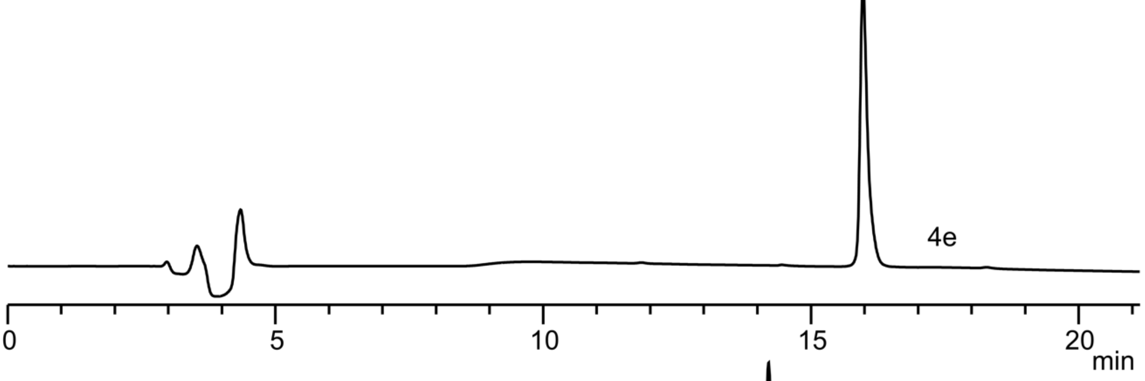
4e
0
5
10
15
20
min

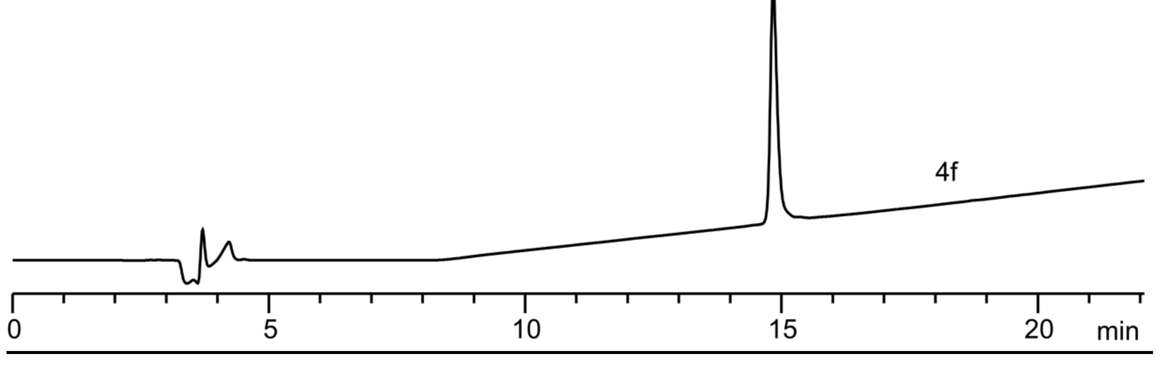
4f
0
5
10
15
20
min

MALDI spectra of all peptides

Peptide **1**

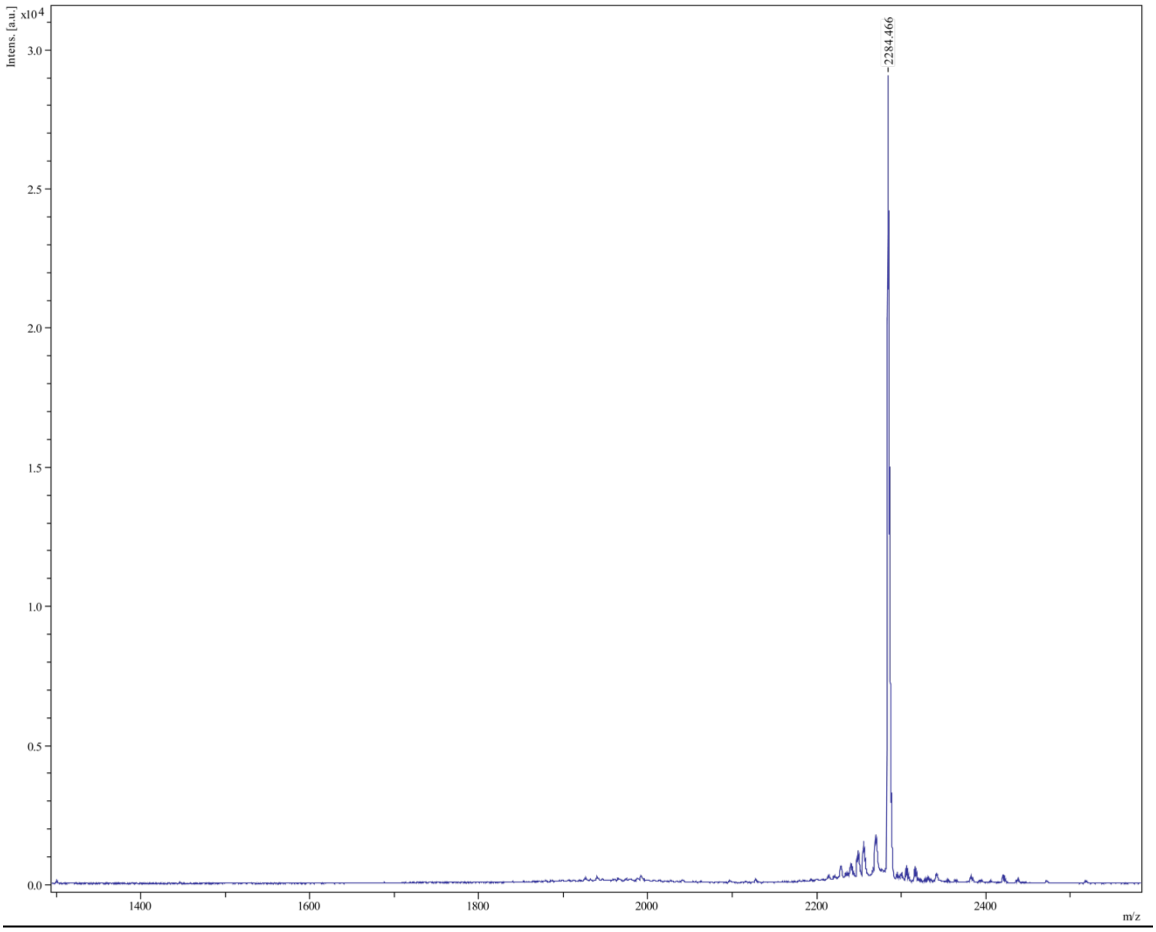

Peptide **1a**

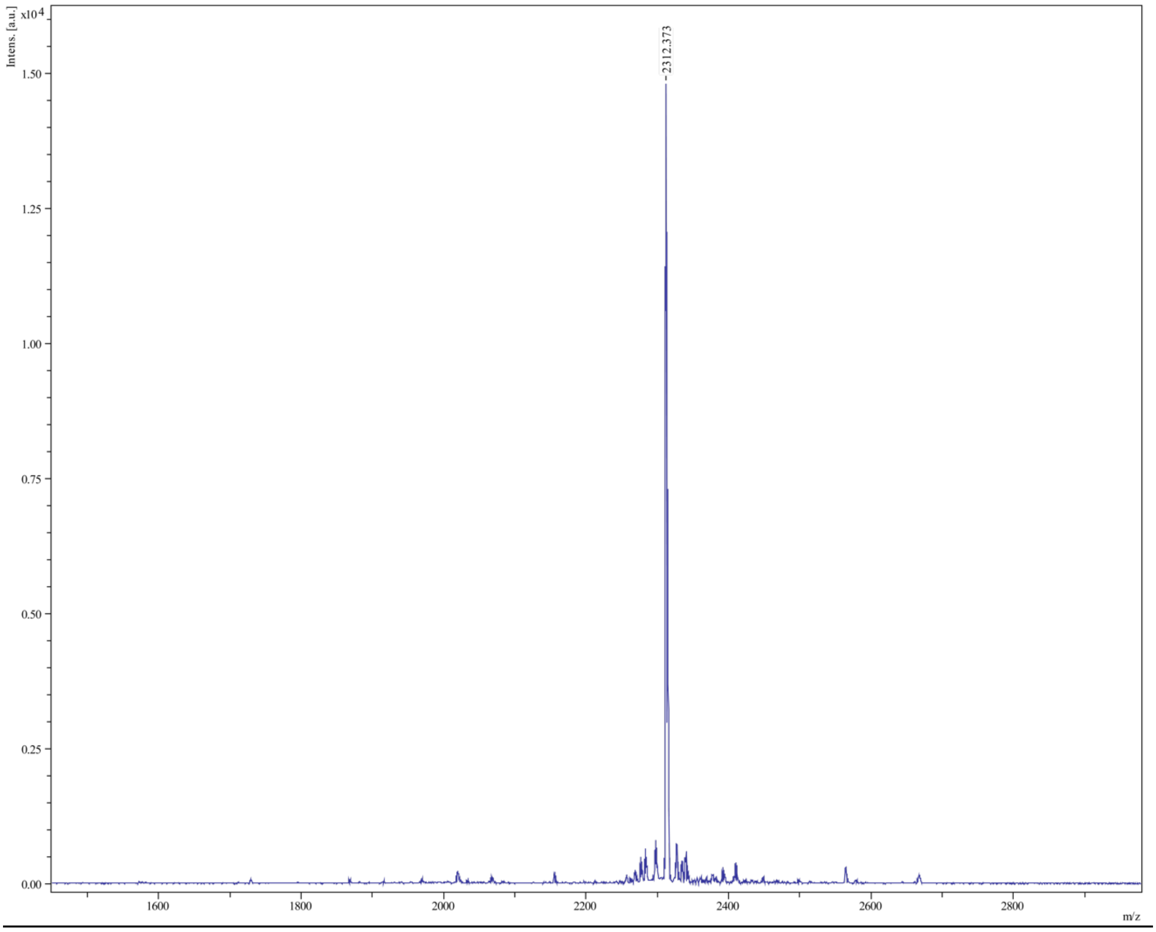

Intens. [a.u.]
x10^4
1.50
1.25
1.00
0.75
0.50
0.25
0.00
2312.373
1600
1800
2000
2200
2400
2600
2800
m/z

Peptide **1b**

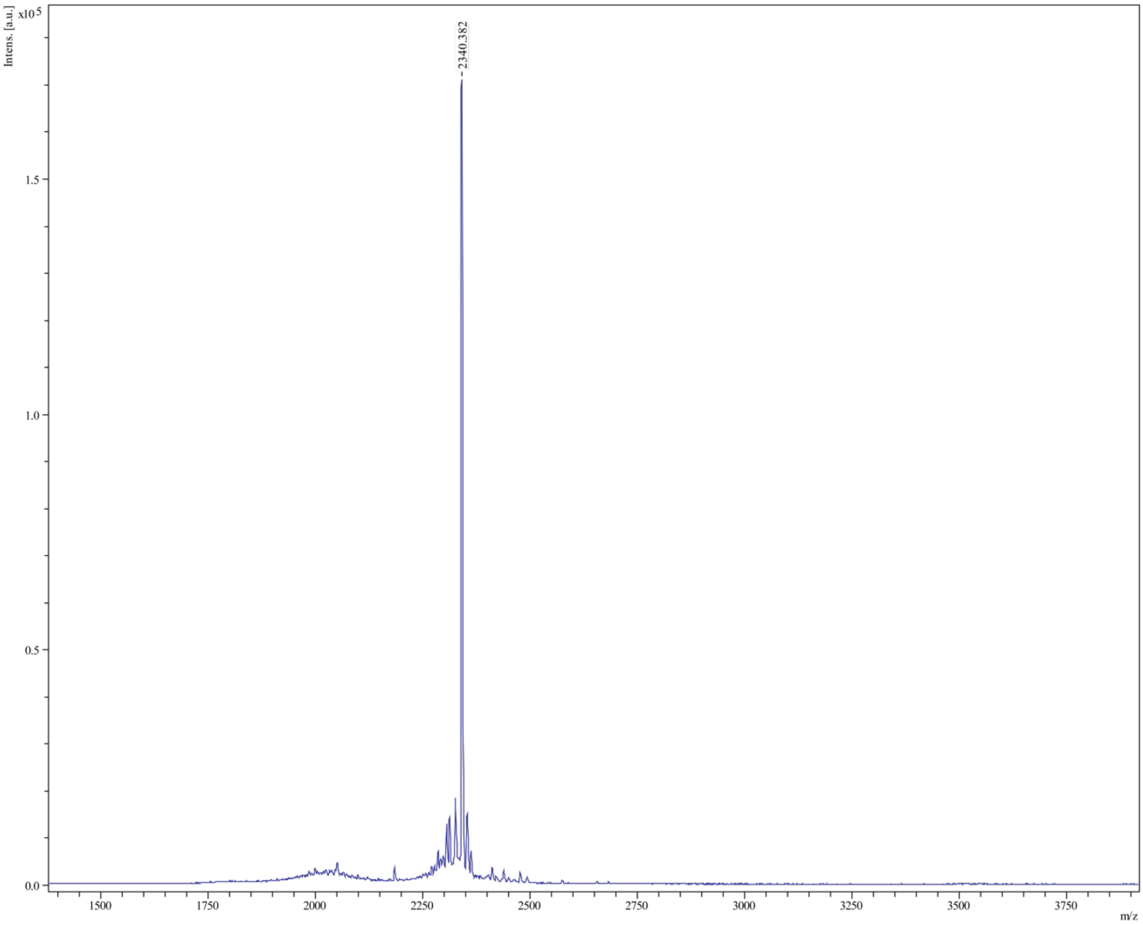
Intens. [a.u.]
x10^5
2340.382
1.5
1.0
0.5
0.0
1500
1750
2000
2250
2500
2750
3000
3250
3500
3750
m/z

Peptide **1c**

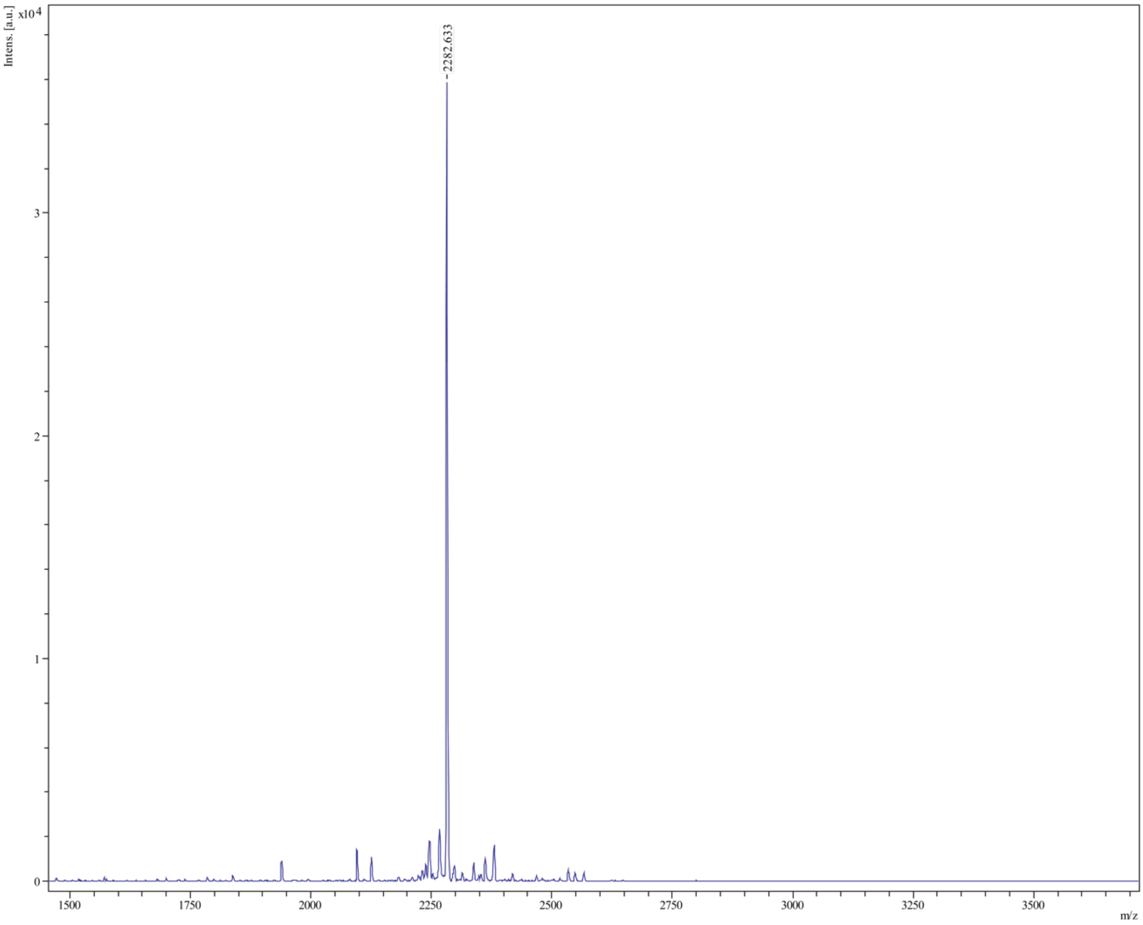

Intens. [a.u.]
x10^4
2282.633
3
2
1
0
1500
1750
2000
2250
2500
2750
3000
3250
3500
m/z

Peptide **1d**

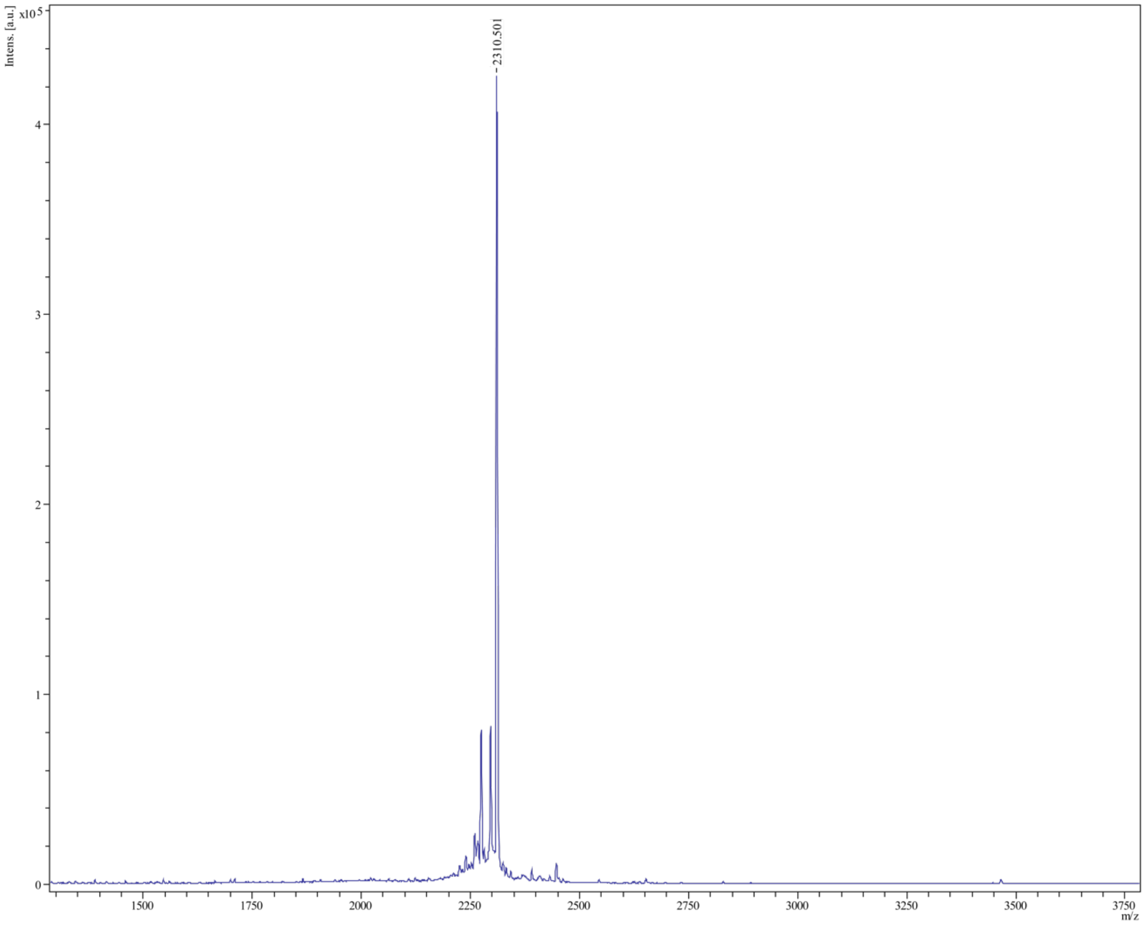

Peptide **1e**

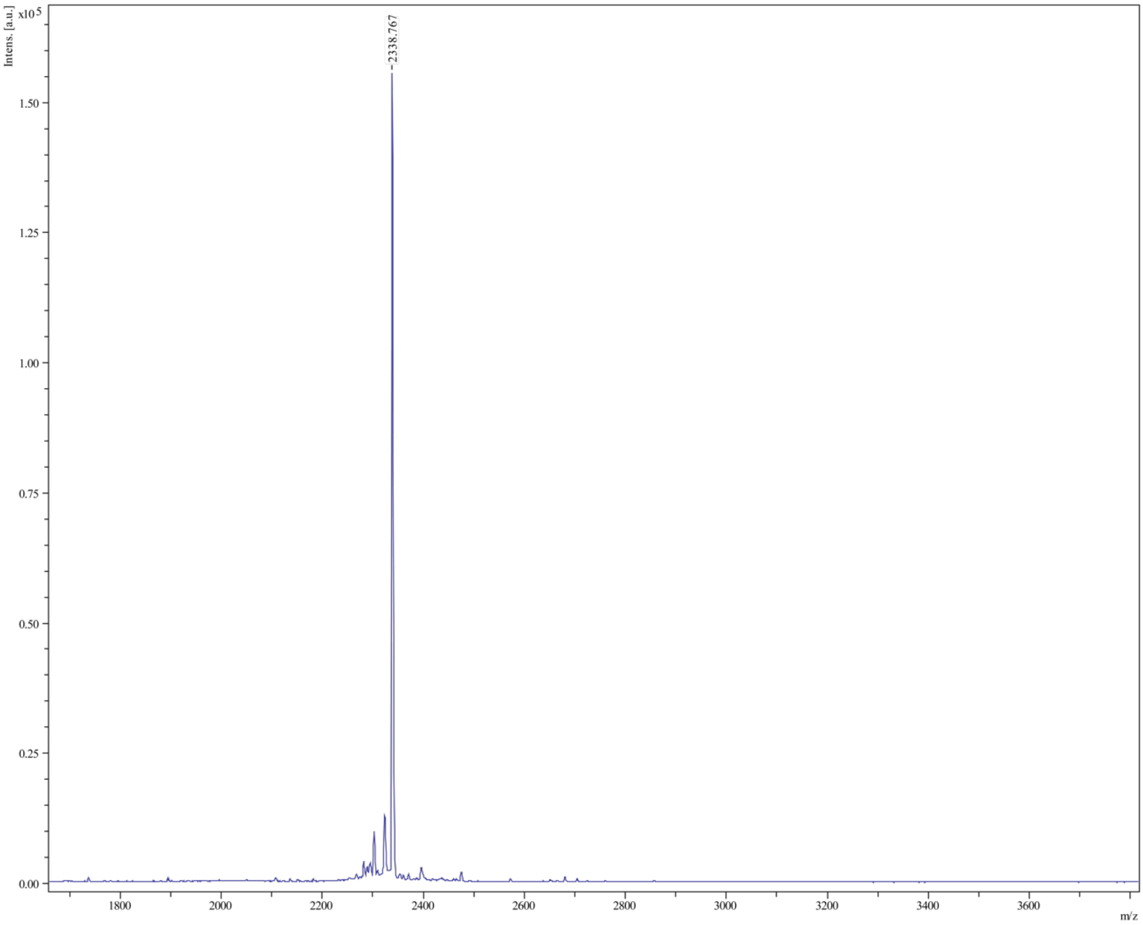

x10^5
Intens. [a.u.]
2338.767
1.50
1.25
1.00
0.75
0.50
0.25
0.00
1800
2000
2200
2400
2600
2800
3000
3200
3400
3600
m/z

Peptide **1f**

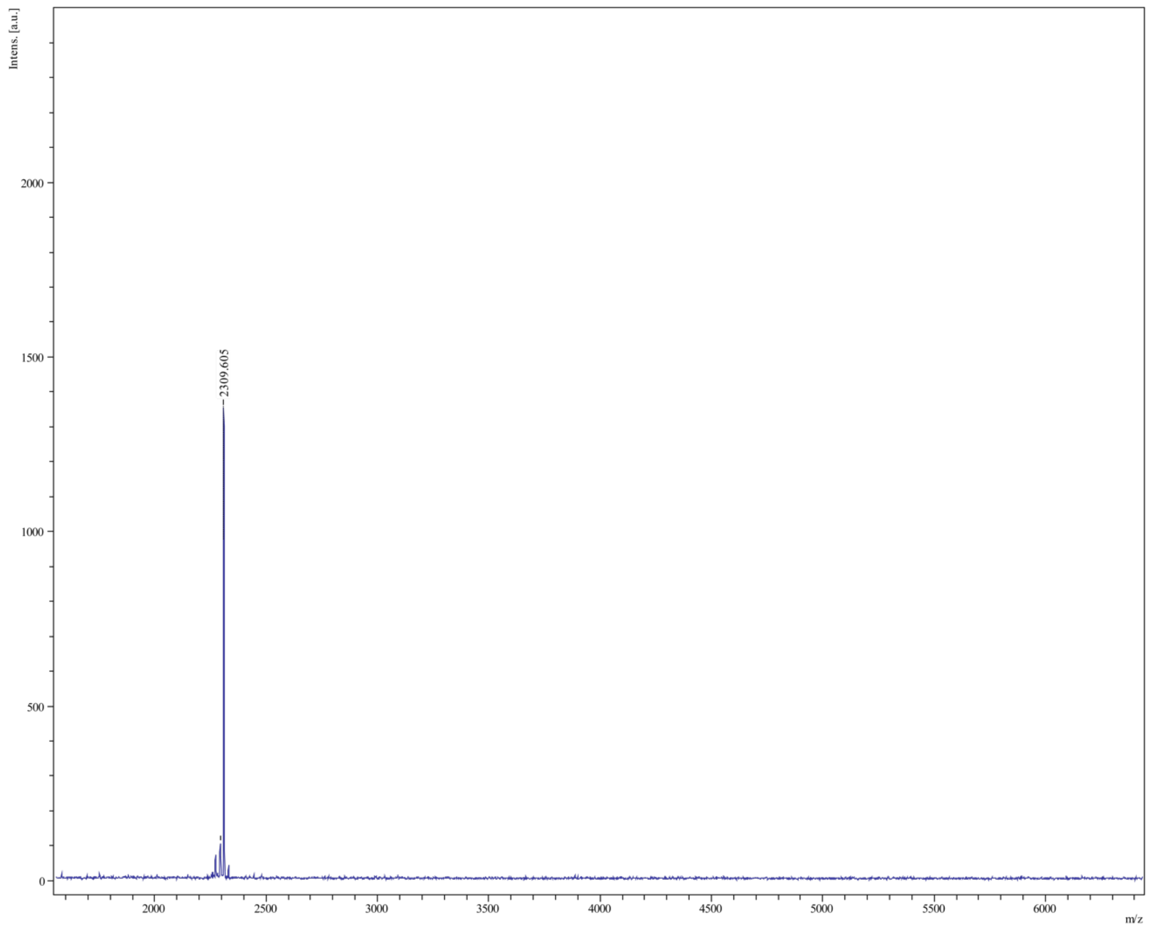
Intens. [a.u.]
2000
1500
1000
500
0
2309.605
2000
2500
3000
3500
4000
4500
5000
5500
6000
m/z

Peptide **2**

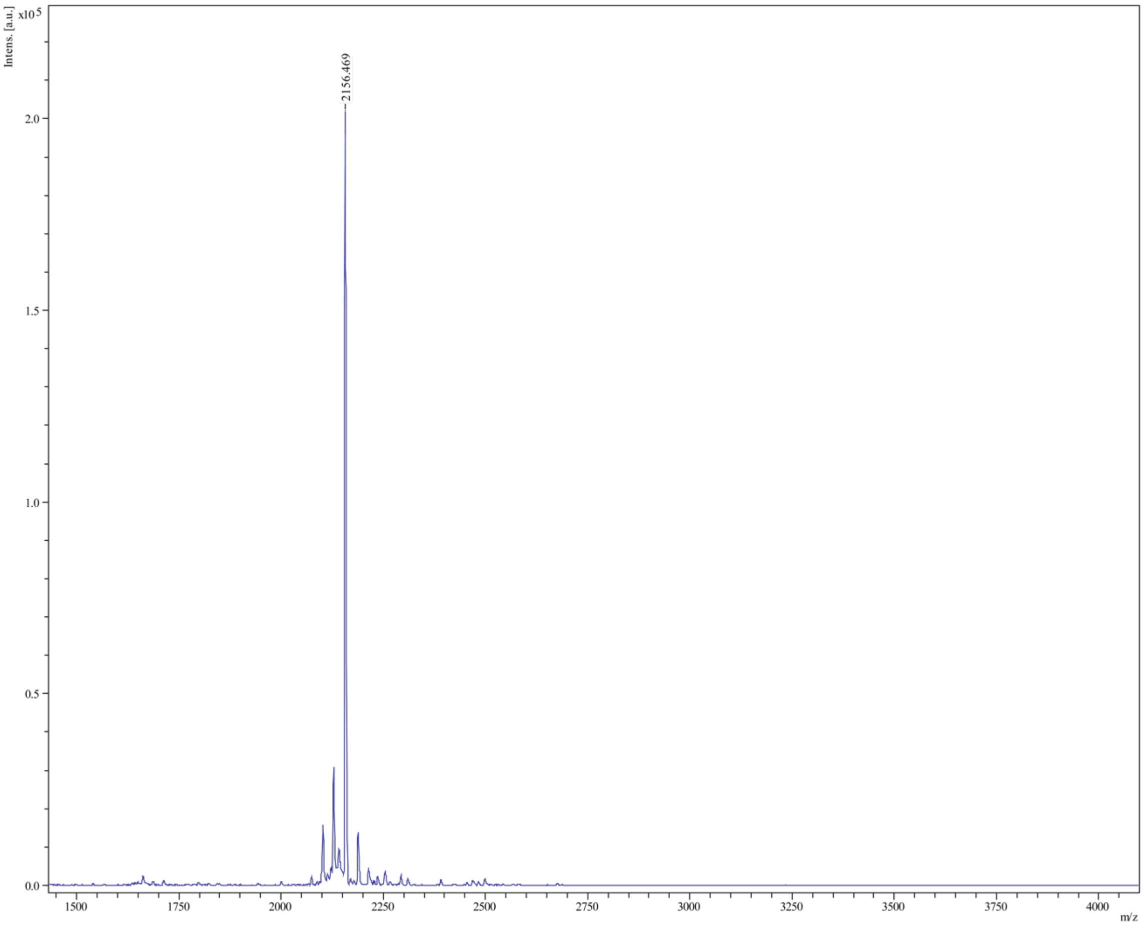

Intens. [a.u.]
x10^5
2156.469
2.0
1.5
1.0
0.5
0.0
1500
1750
2000
2250
2500
2750
3000
3250
3500
3750
4000
m/z

Peptide **2a**

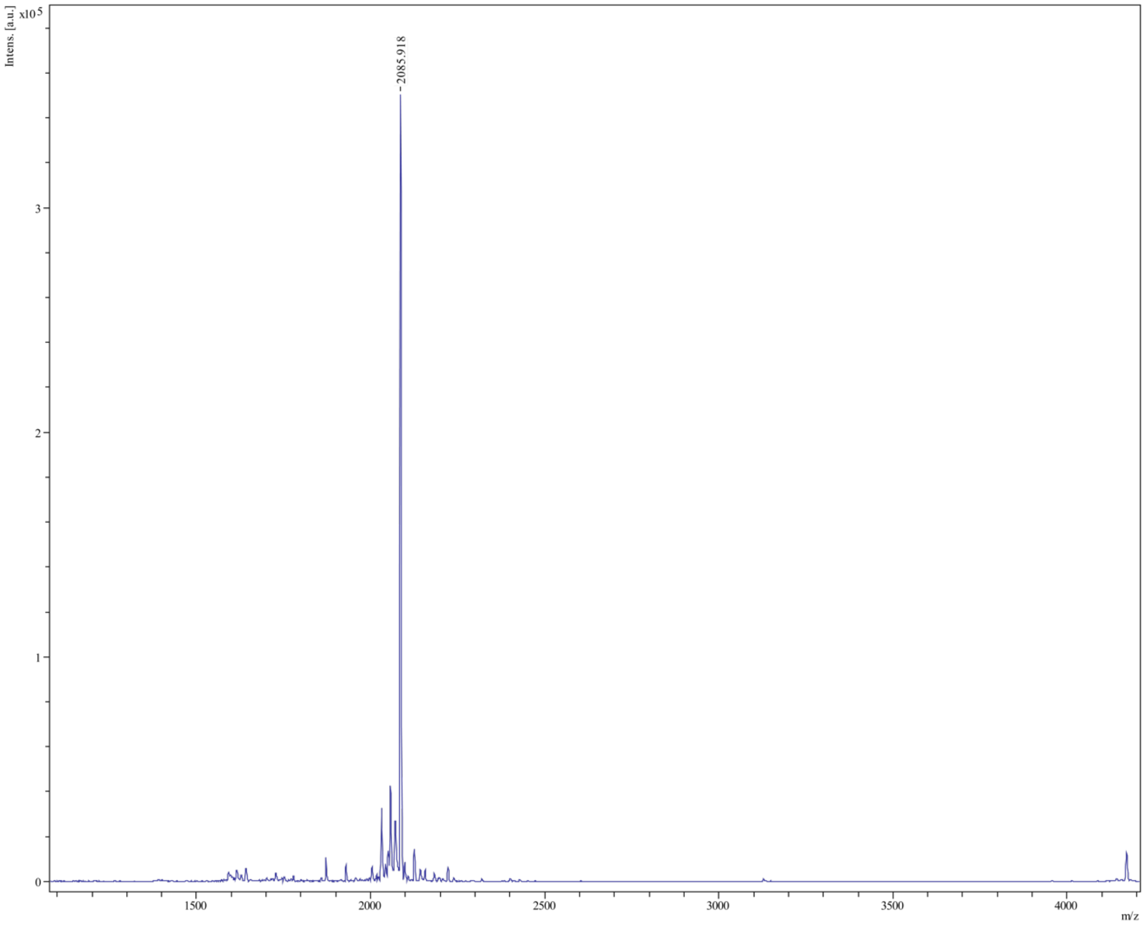

Peptide **2b**

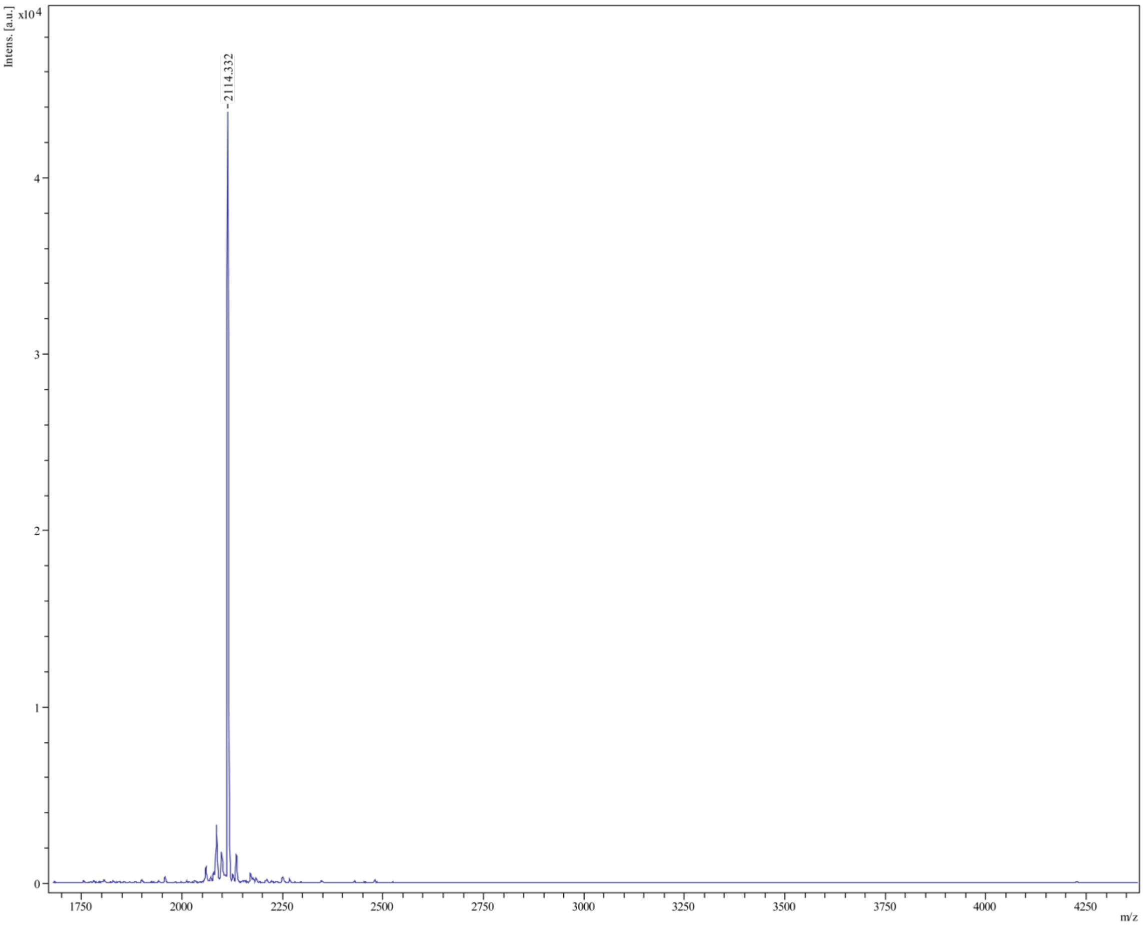

Intens. [a.u.]
x10^4
2114.332
4
3
2
1
0
1750
2000
2250
2500
2750
3000
3250
3500
3750
4000
4250
m/z

Peptide **2c**

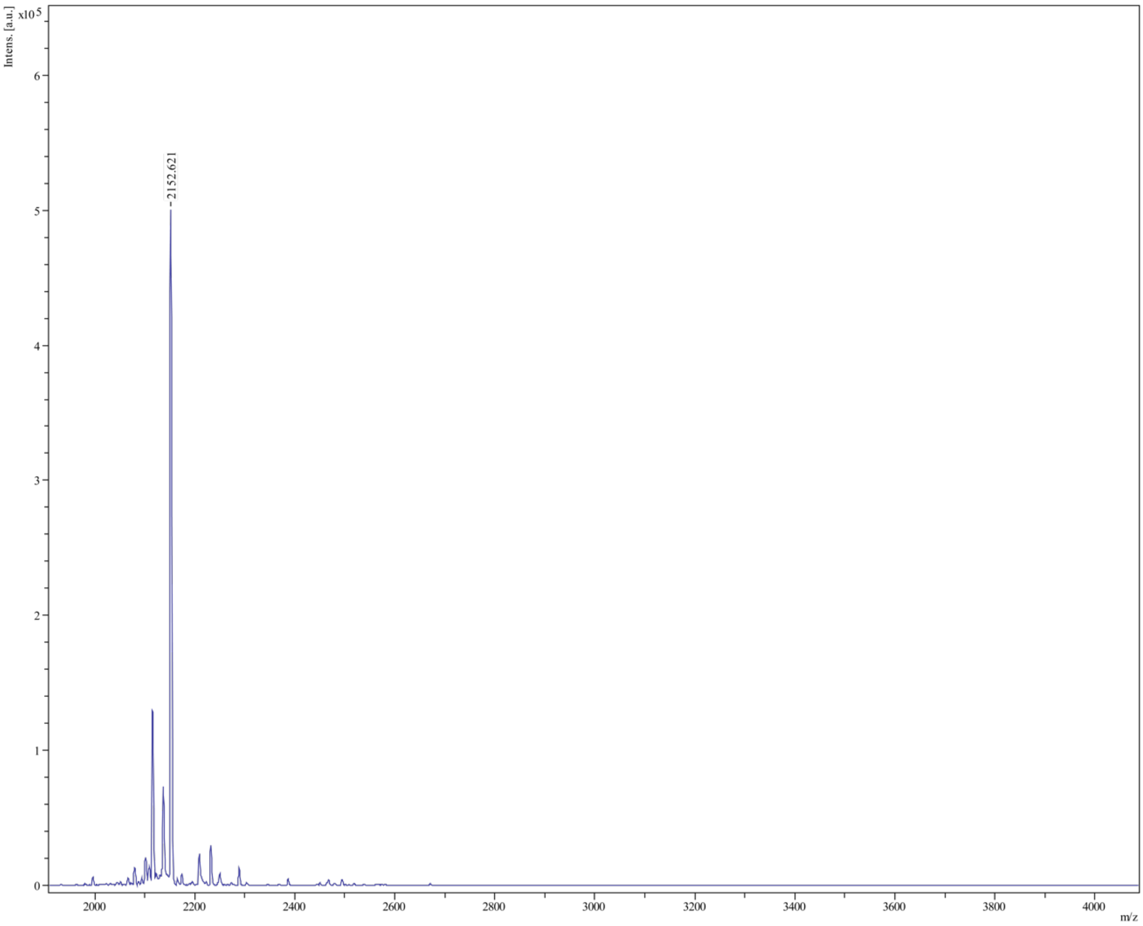

Intens. [a.u.]
x10^5
6
5
4
3
2
1
0
2152.621
2000
2200
2400
2600
2800
3000
3200
3400
3600
3800
4000
m/z

Peptide **2d**

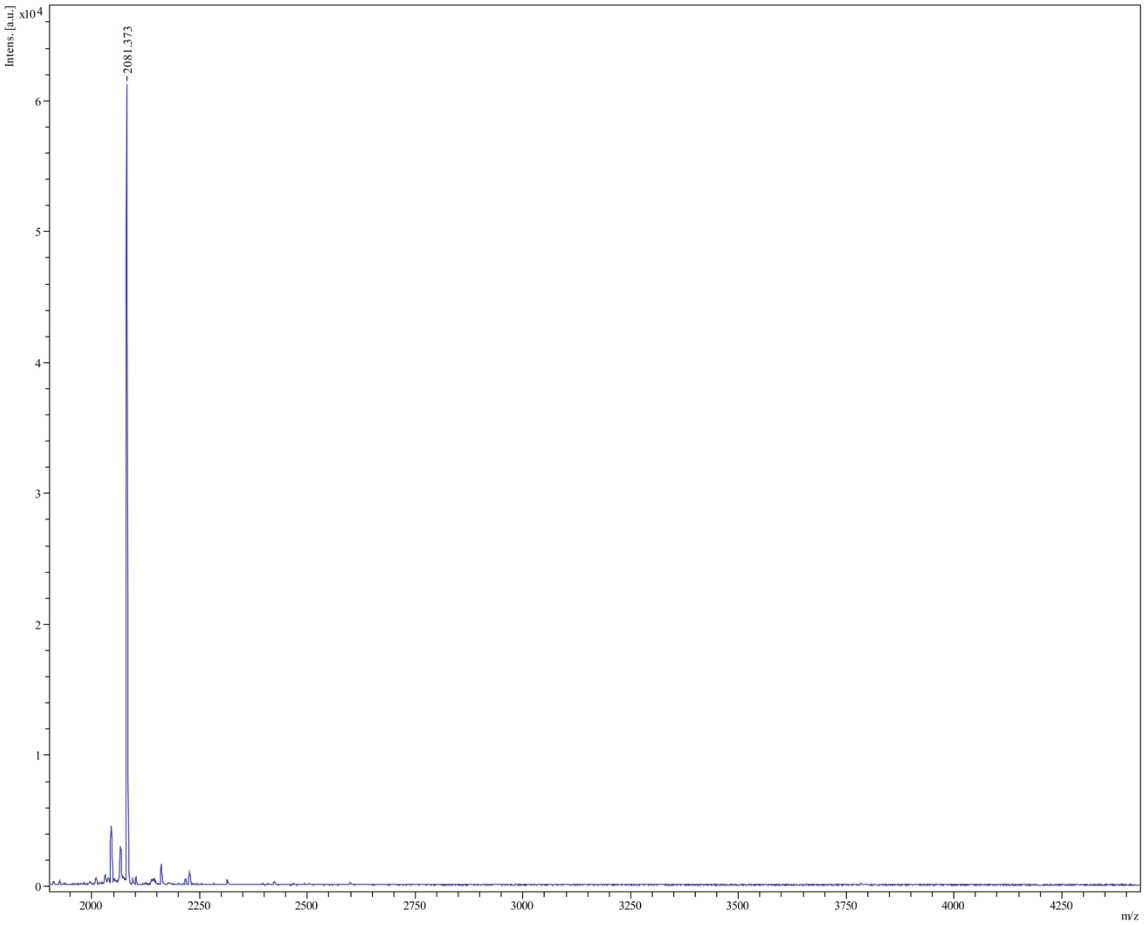
Intens. [a.u.]
x10^4
2081.373
0
1
2
3
4
5
6
2000
2250
2500
2750
3000
3250
3500
3750
4000
4250
m/z

Peptide **2e**

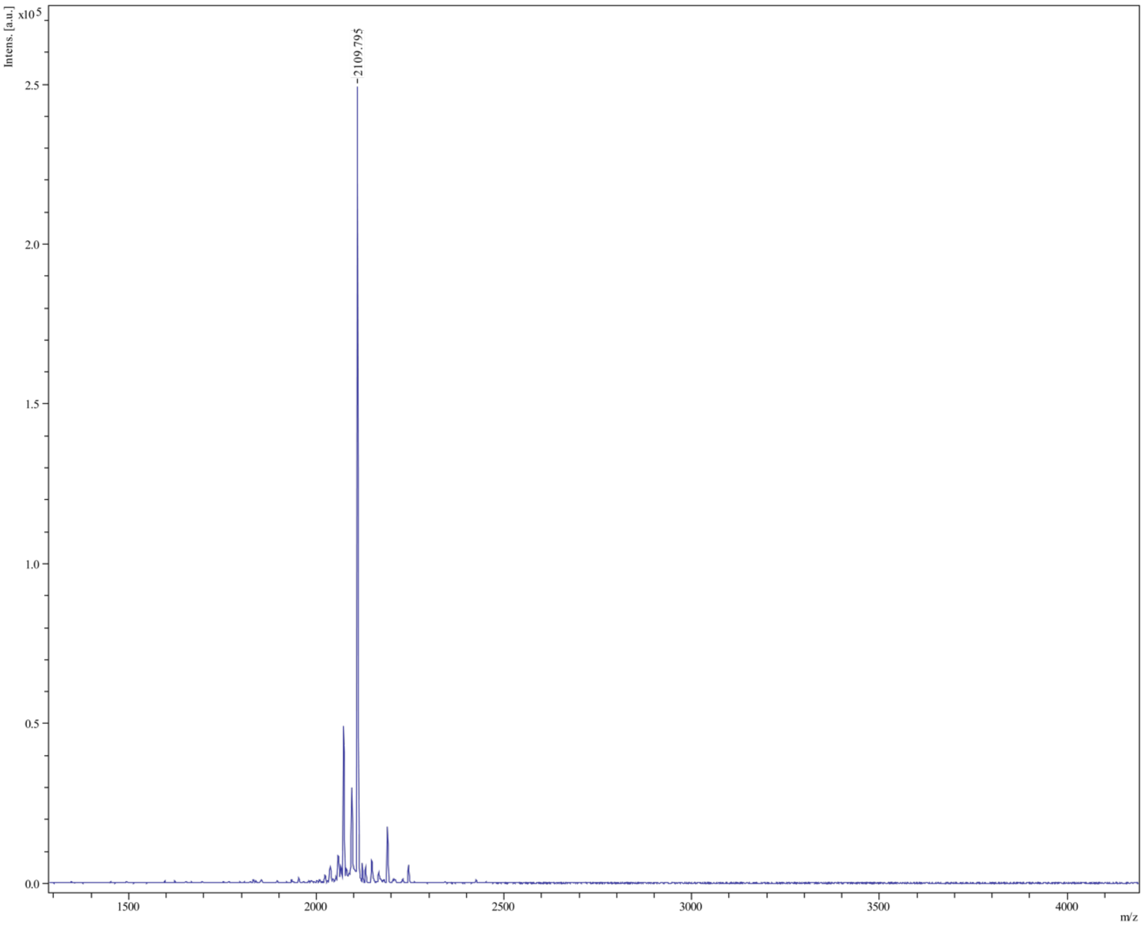

Intens. [a.u.]
x10^5
2.5
2.0
1.5
1.0
0.5
0.0
2109.795
1500
2000
2500
3000
3500
4000
m/z

Peptide **2f**

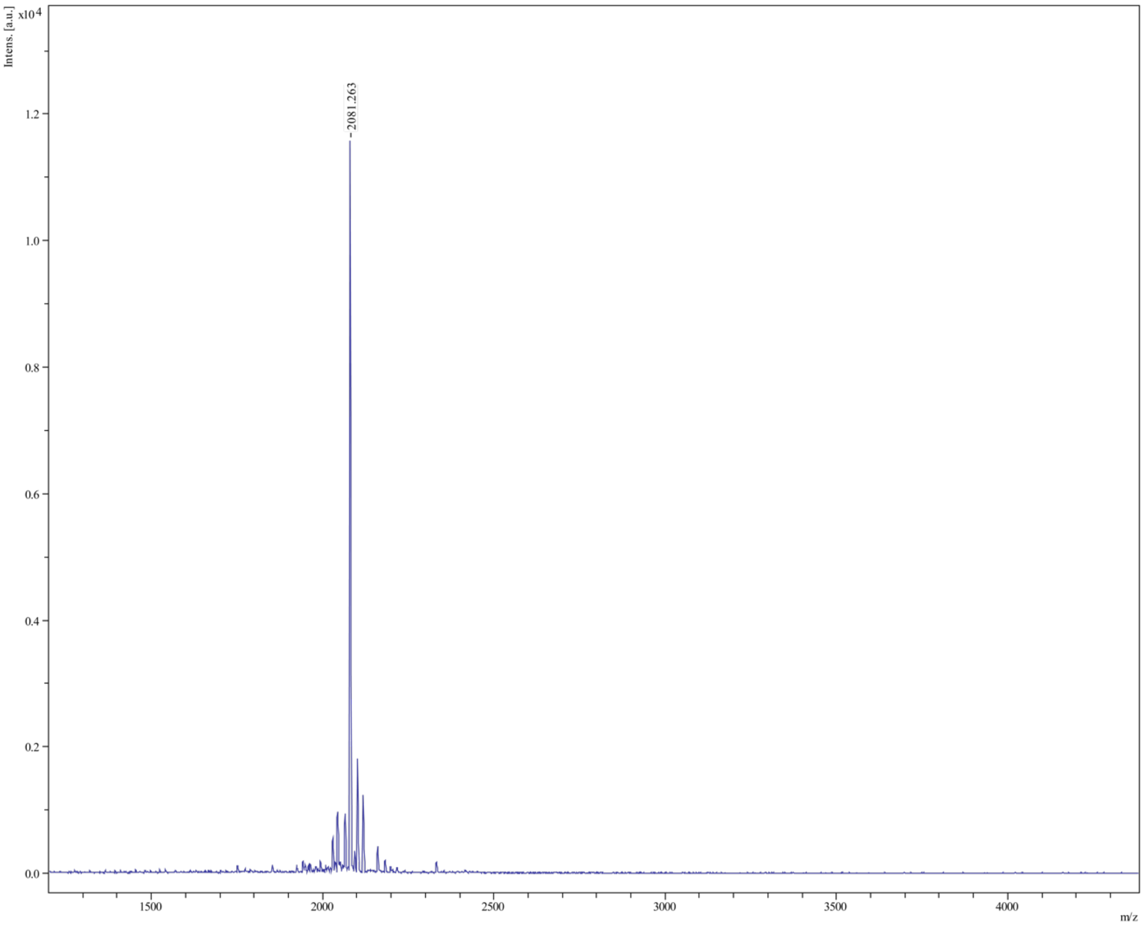
Intens. [a.u.]
x10^4
1.2
1.0
0.8
0.6
0.4
0.2
0.0
2081.263
1500
2000
2500
3000
3500
4000
m/z

Peptide **3**

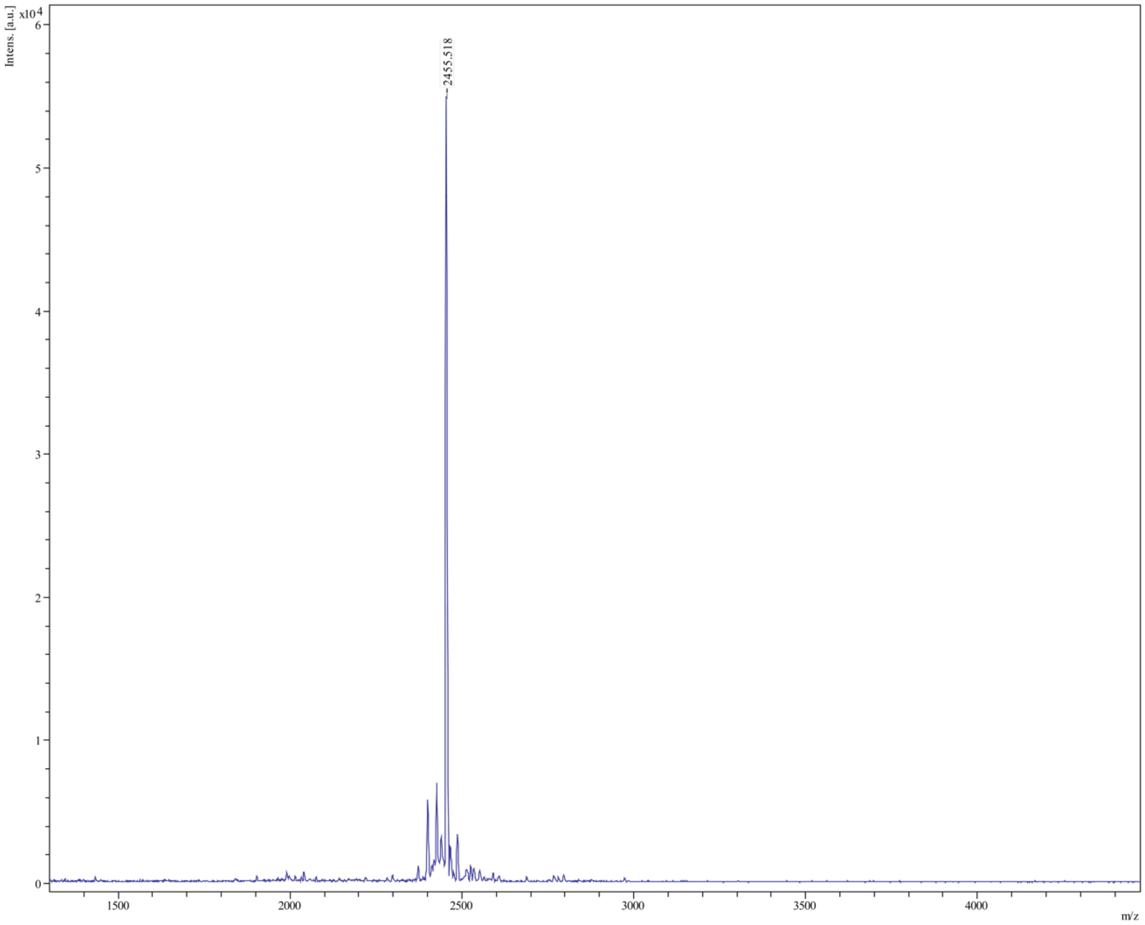

Intens. [a.u.]
x10^4
6
5
4
3
2
1
0
2455.518
1500
2000
2500
3000
3500
4000
m/z

Peptide **3a**

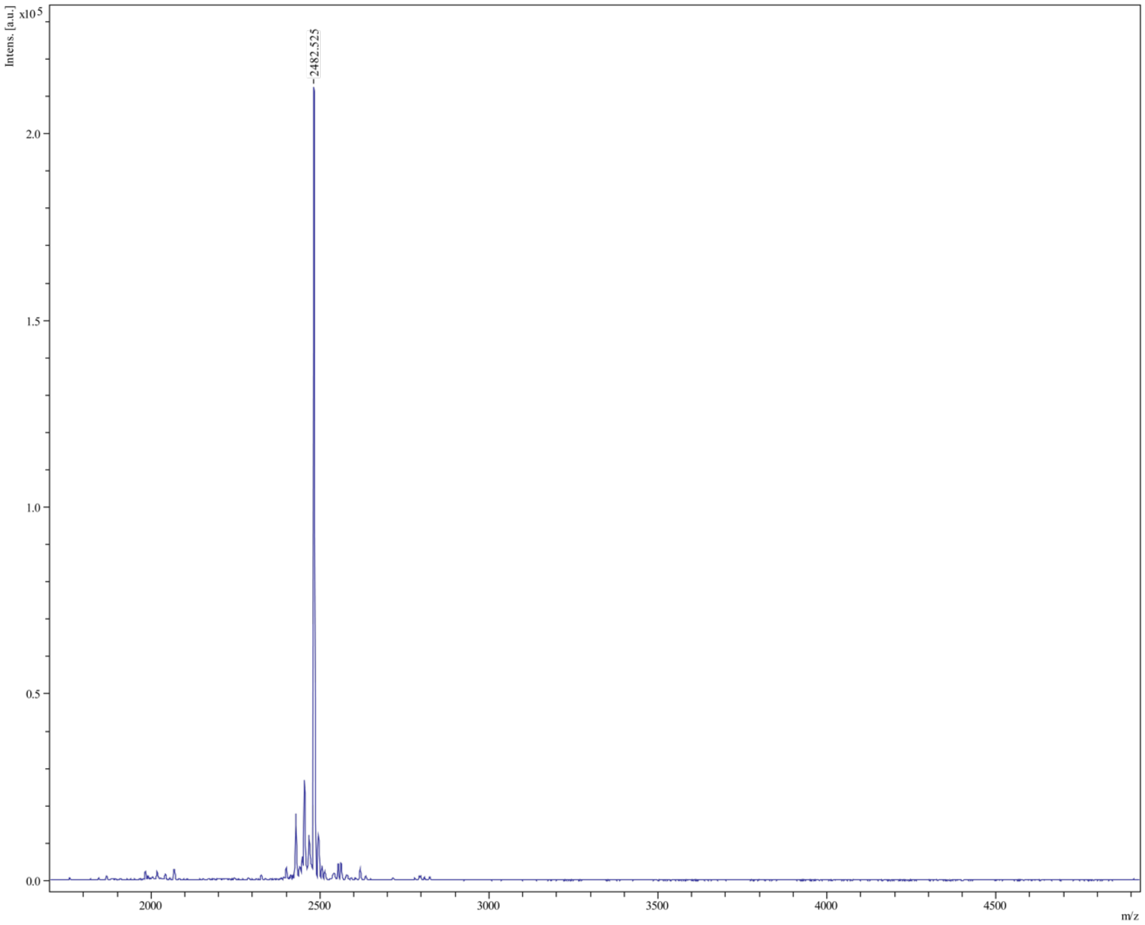

Intens. [a.u.]
x10^5
2482.525
2.0
1.5
1.0
0.5
0.0
2000
2500
3000
3500
4000
4500
m/z

Peptide **3b**

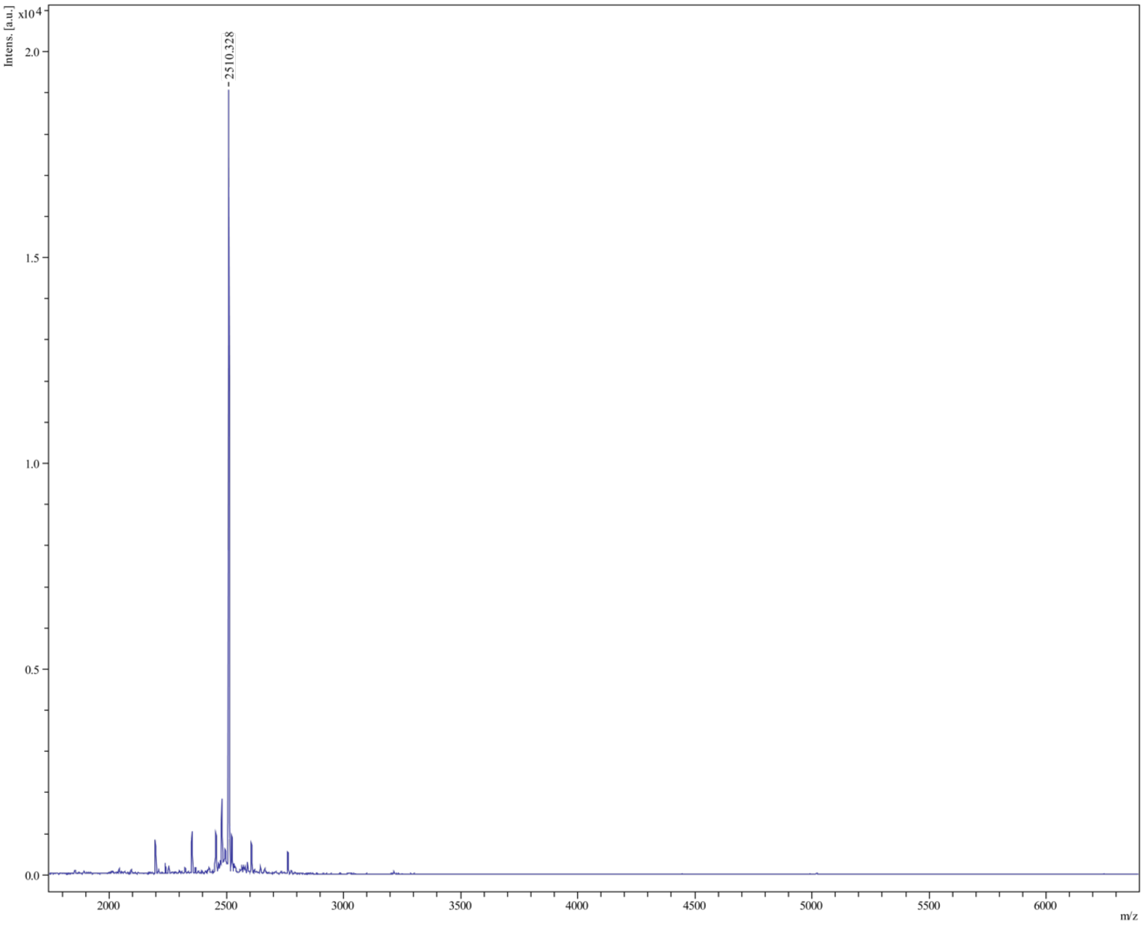
Intens. [a.u.]
x10^4
2.0
1.5
1.0
0.5
0.0
2510.328
2000
2500
3000
3500
4000
4500
5000
5500
6000
m/z

Peptide **3c**

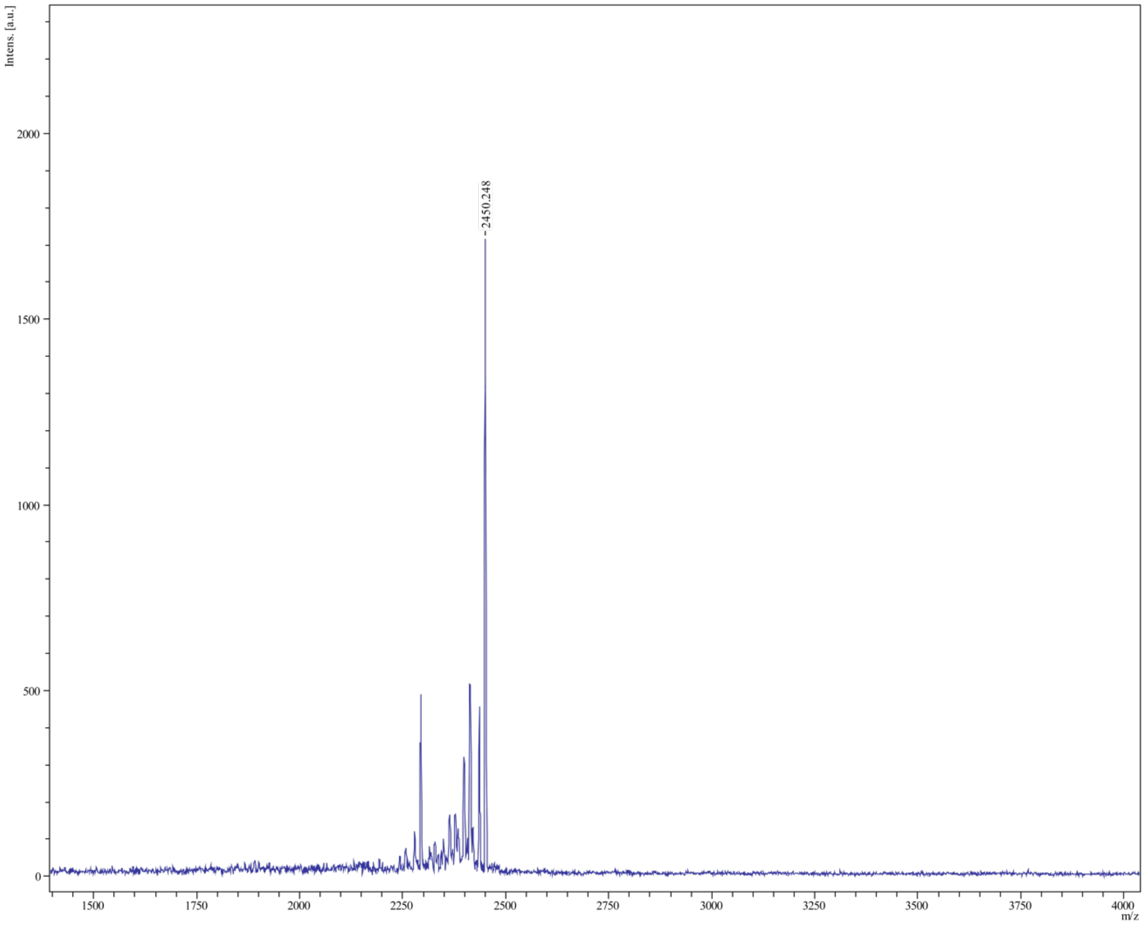

Intens. [a.u.]
2000
1500
1000
500
0
2450.248
1500
1750
2000
2250
2500
2750
3000
3250
3500
3750
4000
m/z

Peptide **3d**

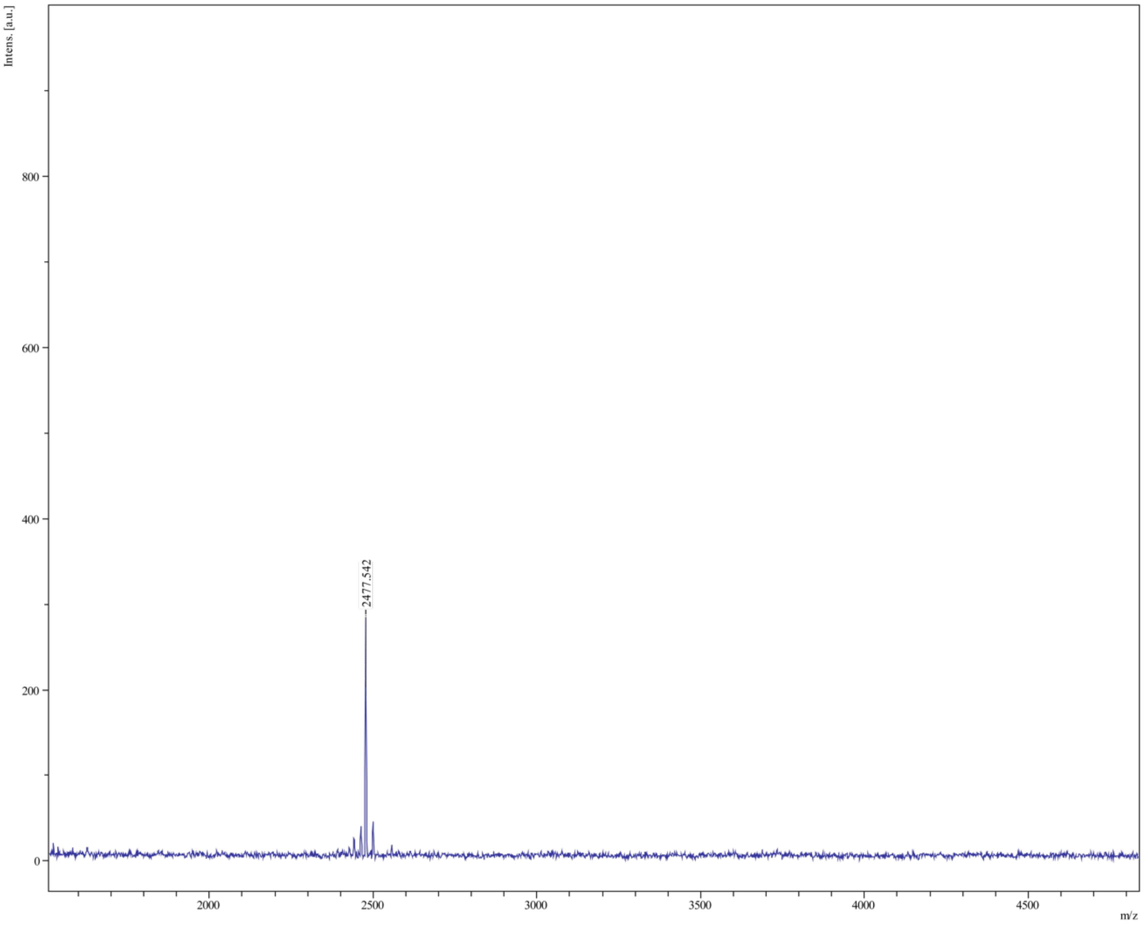
Intens. [a.u.]
800
600
400
200
0
2477.542
2000
2500
3000
3500
4000
4500
m/z

Peptide **3e**

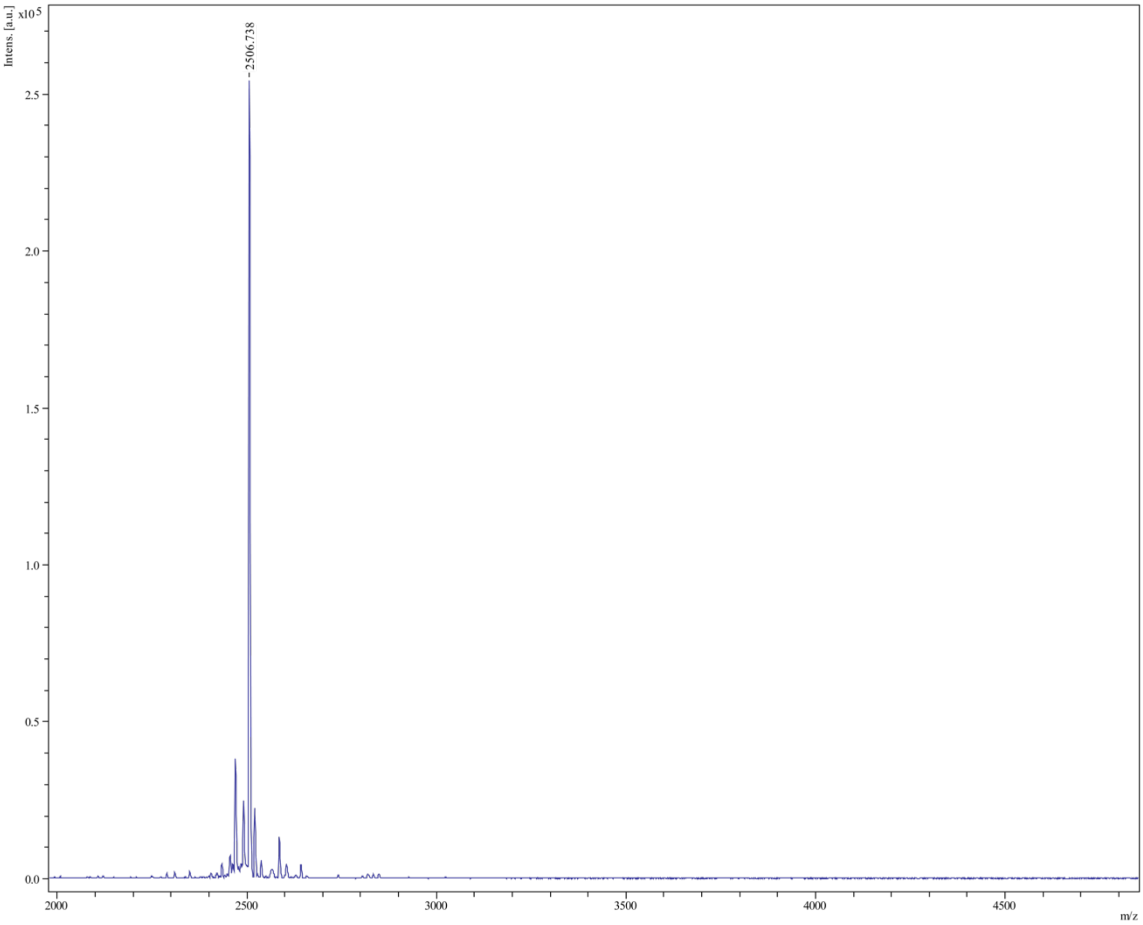
Intens. [a.u.]
x10^5
2506.738
2.5
2.0
1.5
1.0
0.5
0.0
2000
2500
3000
3500
4000
4500
m/z

Peptide **3f**

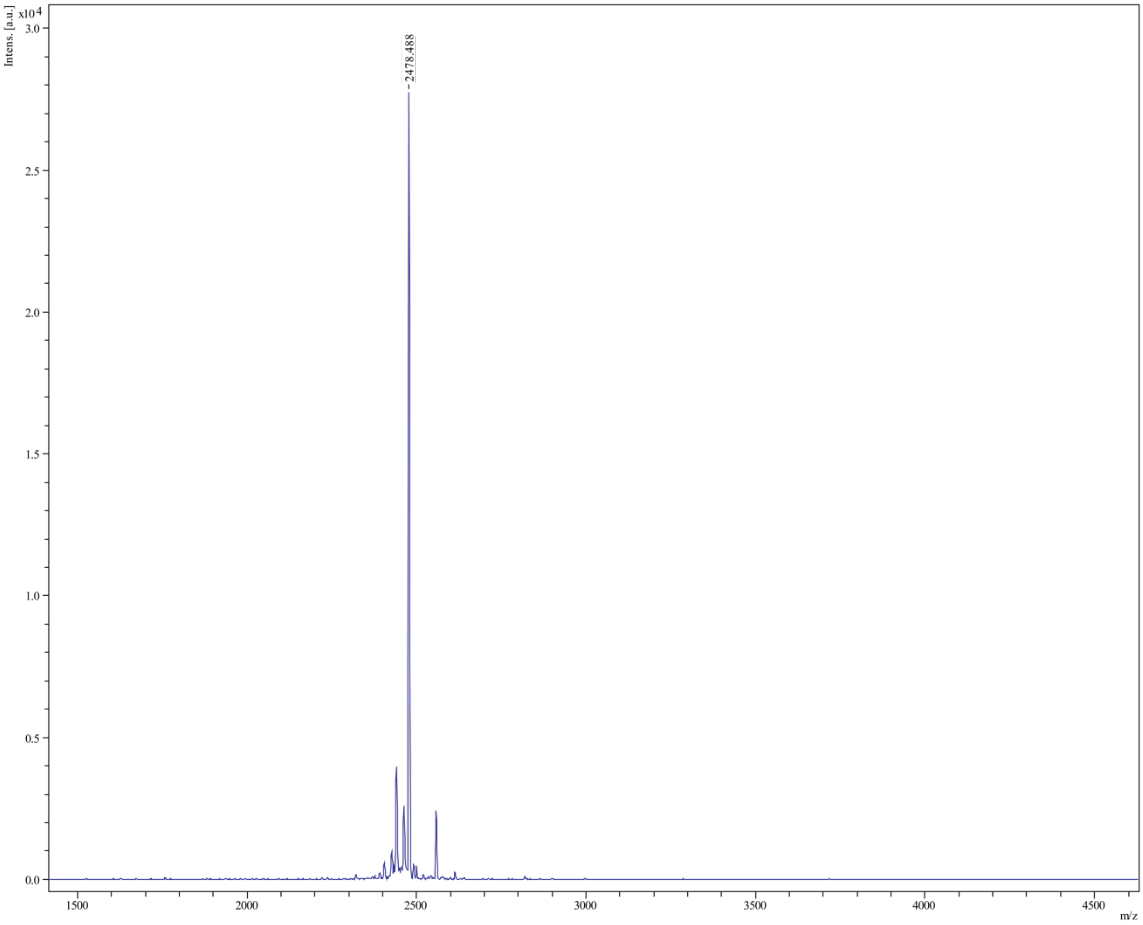
Intens. [a.u.]
x10^4
3.0
2.5
2.0
1.5
1.0
0.5
0.0
2478.488
1500
2000
2500
3000
3500
4000
4500
m/z

Peptide **4**

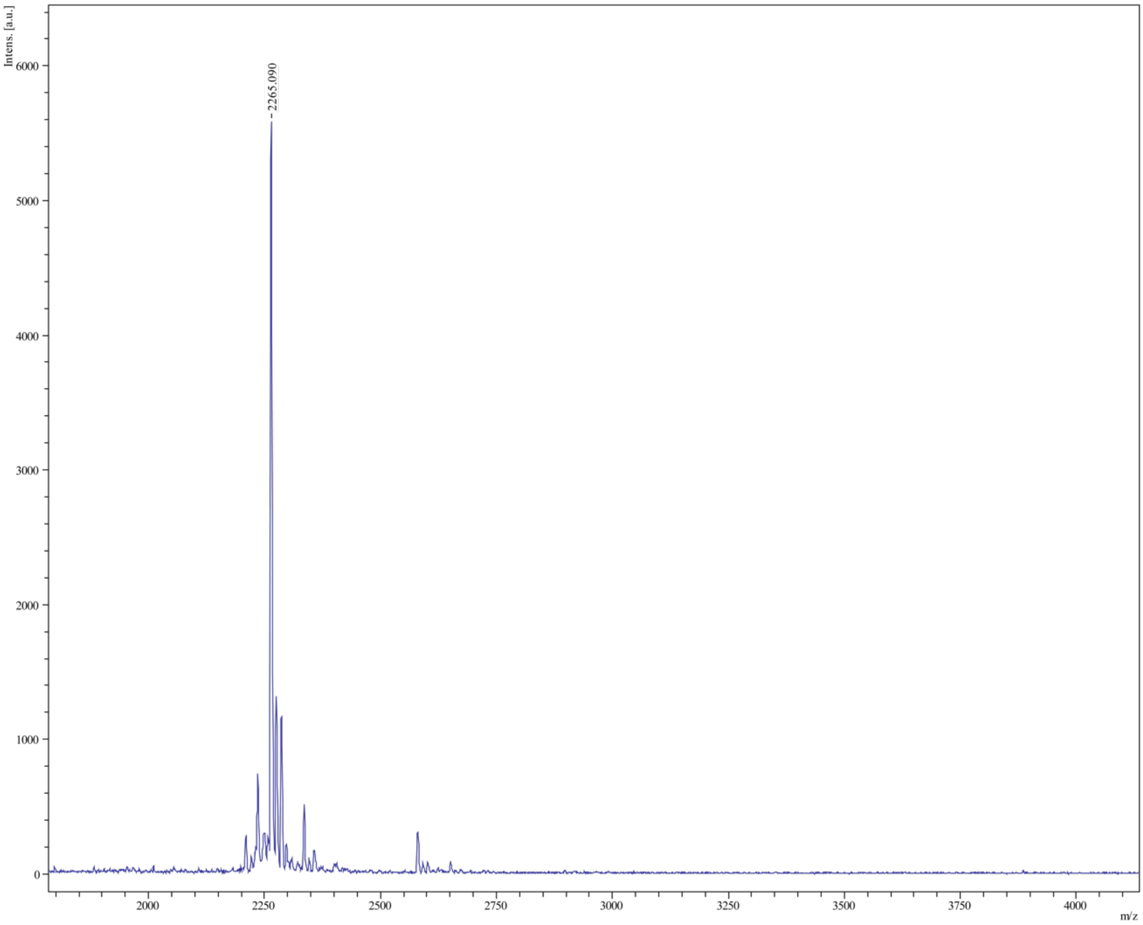

Intens. [a.u.]
6000
5000
4000
3000
2000
1000
0
2265.090
2000
2250
2500
2750
3000
3250
3500
3750
4000
m/z

Peptide **4a**

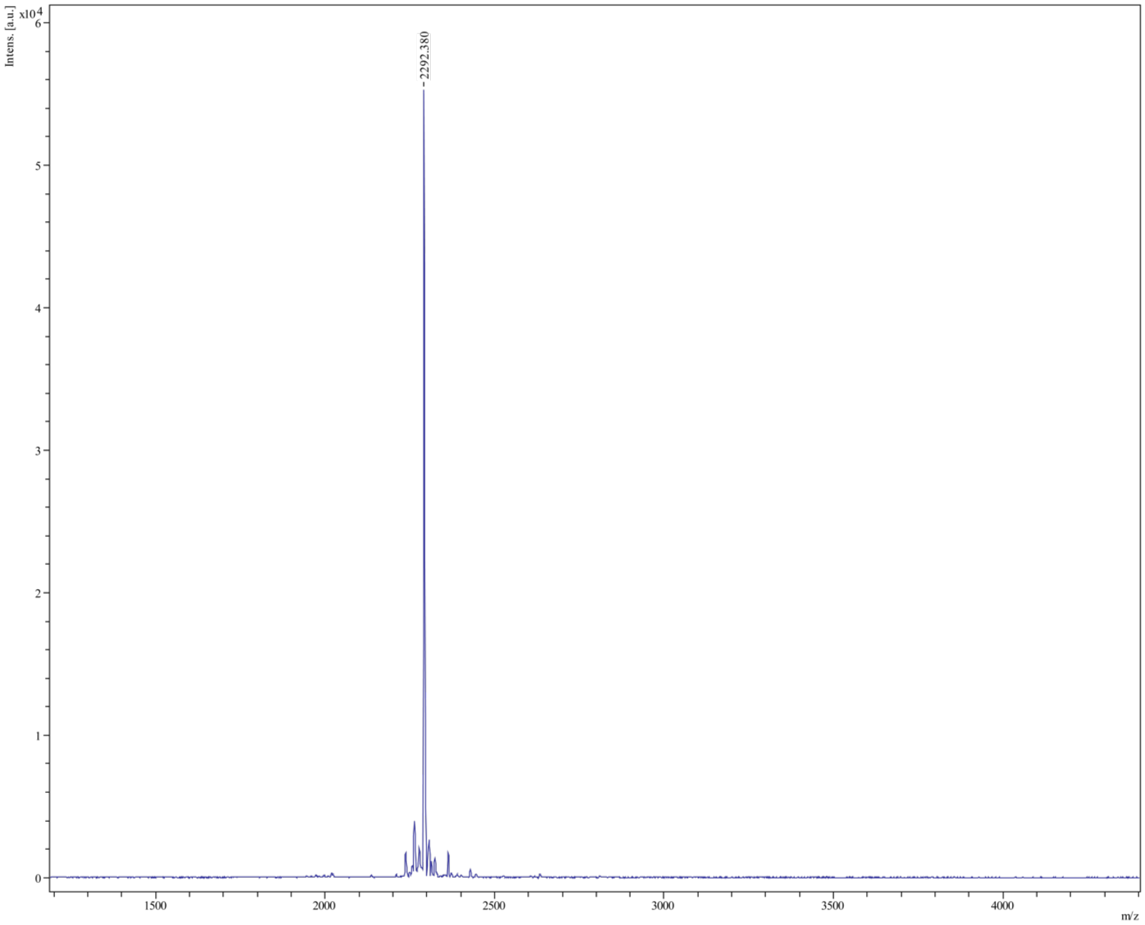

Peptide **4b**

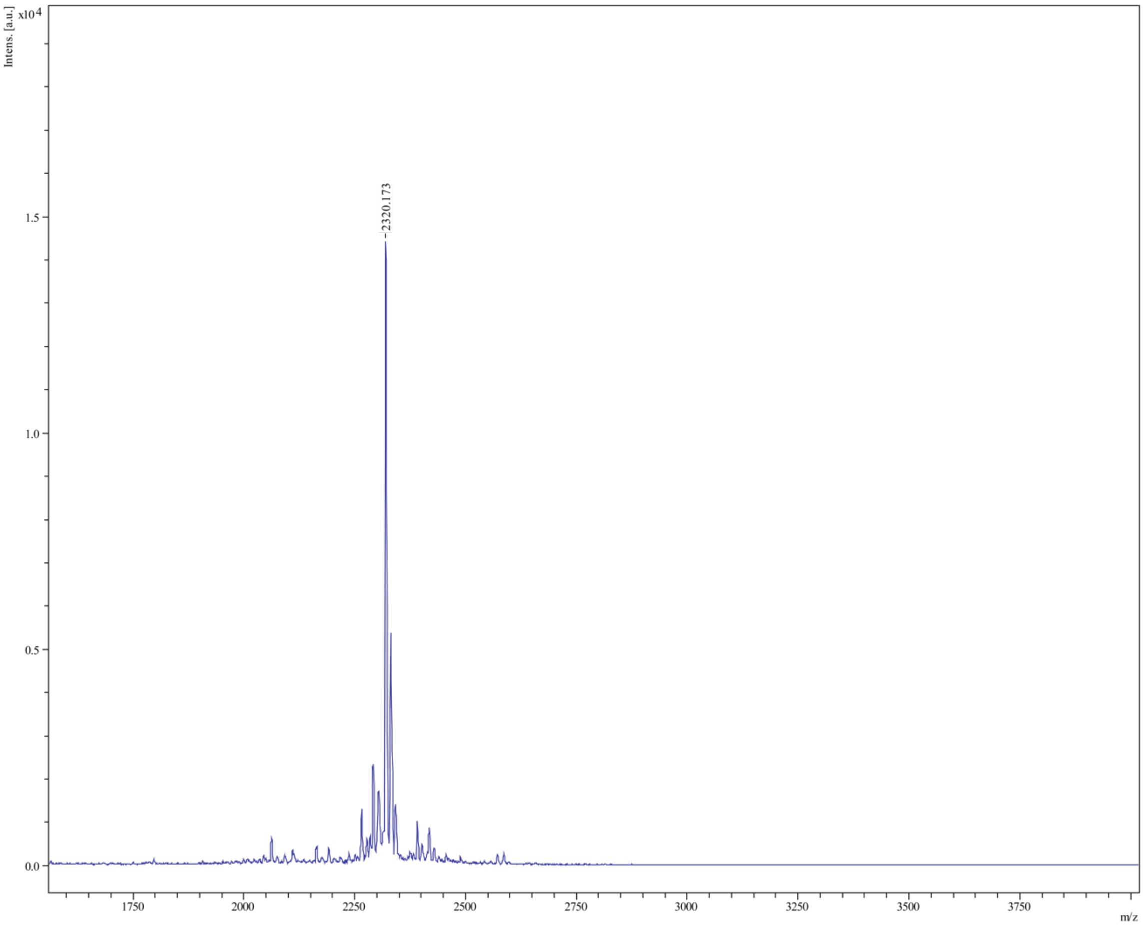

Intens. [a.u.]
x10^4
1.5
1.0
0.5
0.0
2320.173
1750
2000
2250
2500
2750
3000
3250
3500
3750
m/z

Peptide **4c**

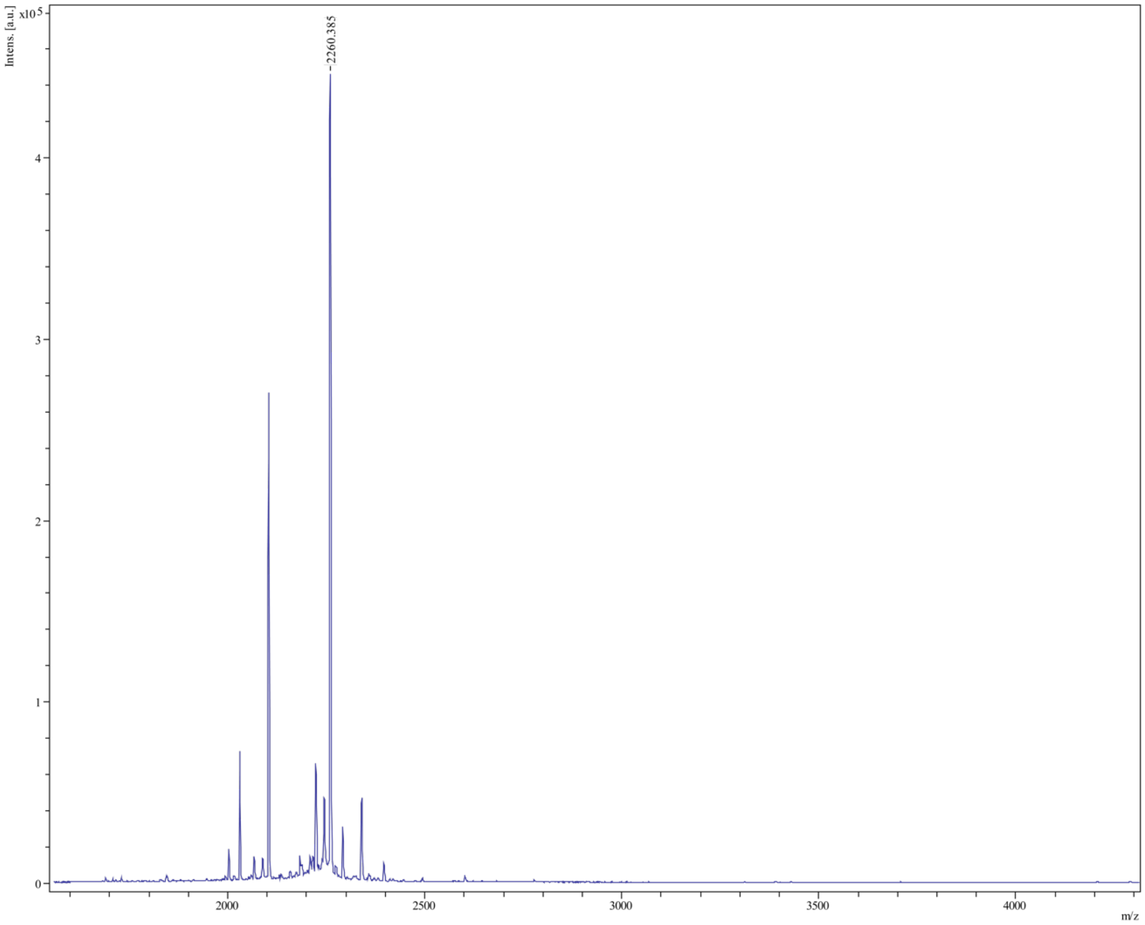

Peptide **4d**

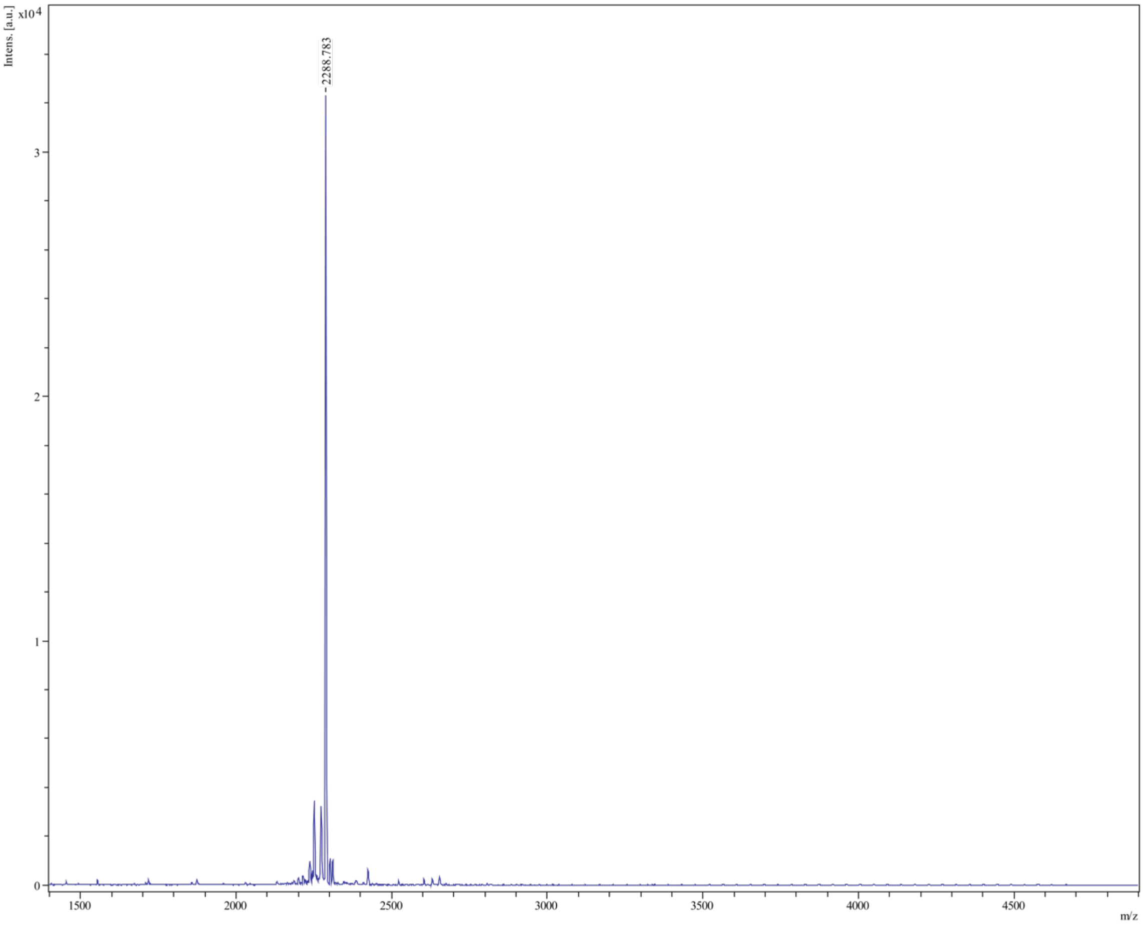

Intens. [a.u.]
x10^4
3
2
1
0
2288.783
1500
2000
2500
3000
3500
4000
4500
m/z

Peptide **4e**

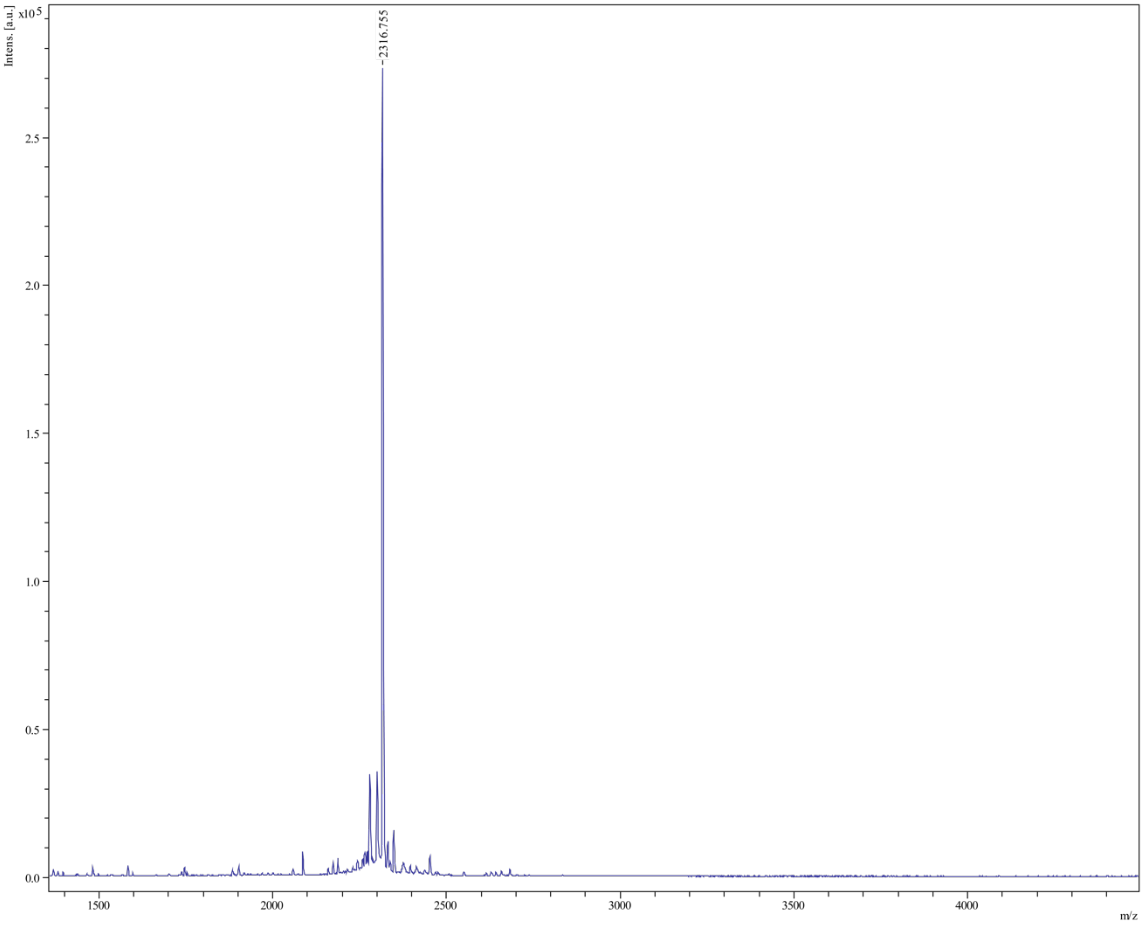

Intens. [a.u.]
x10^5
2316.755
2.5
2.0
1.5
1.0
0.5
0.0
1500
2000
2500
3000
3500
4000
m/z

Peptide **4f**

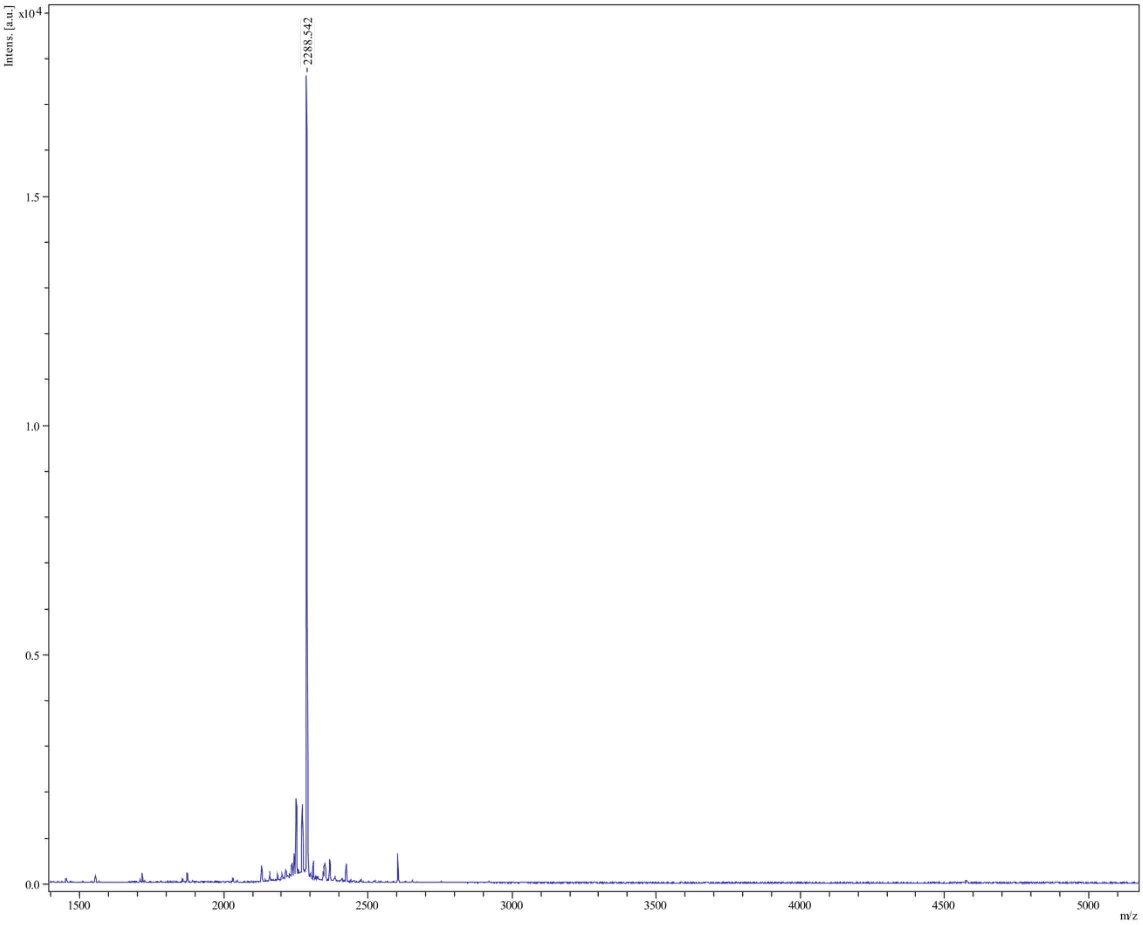

Intens. [a.u.]
x10^4
2288.542
1.5
1.0
0.5
0.0
1500
2000
2500
3000
3500
4000
4500
5000
m/z

<u>**^{1}H NMR spectra**</u>

Peptide **1**(9:1 H_2O:D_2O)

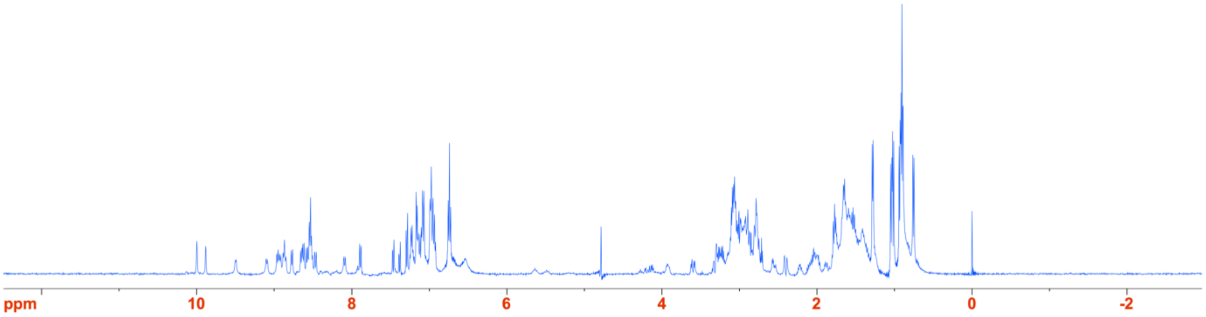

Peptide **1a**(9:1 H_2O:D_2O)

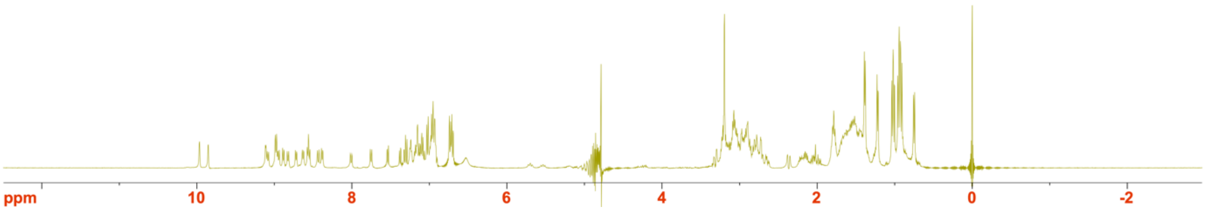

Peptide **1b**(9:1 H_2O:D_2O)

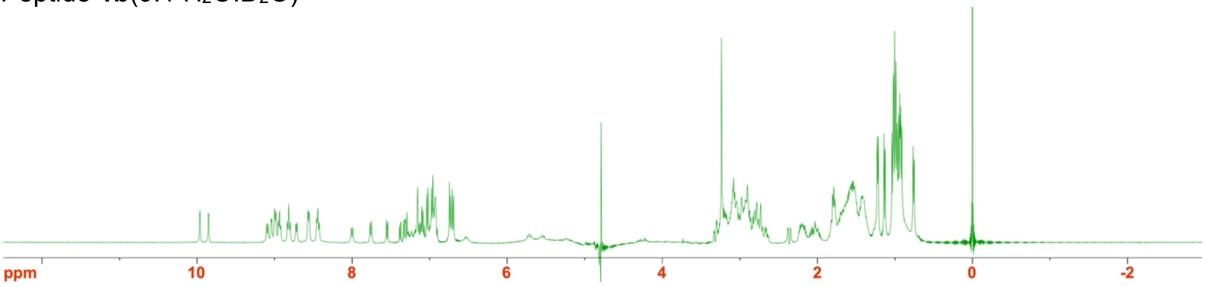

Peptide **1c**(9:1 H_2O:D_2O)

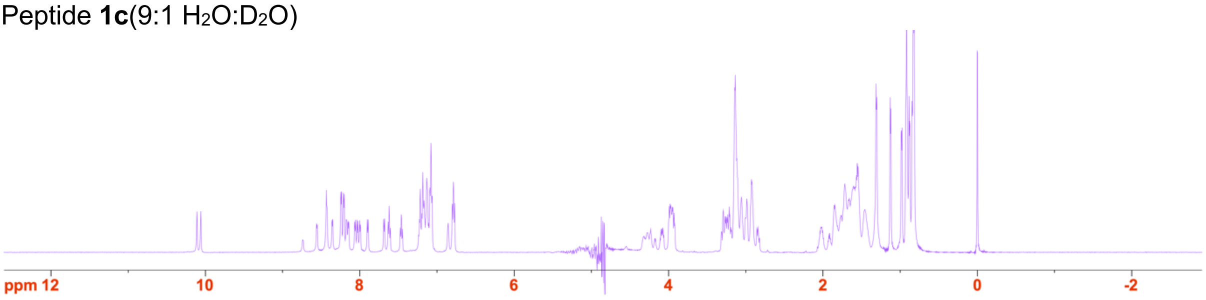

Peptide **1d**(9:1 H_2O:D_2O)

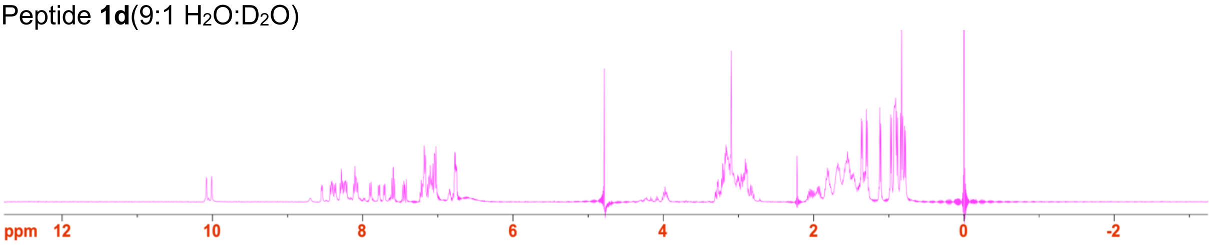

Peptide **1e**(9:1 H_2O:D_2O)

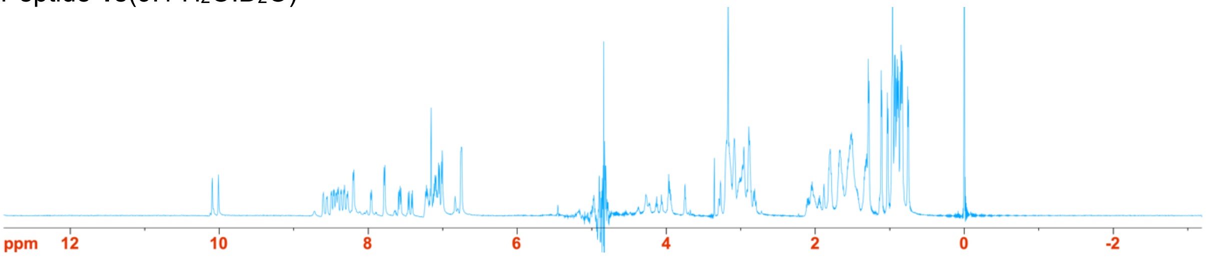

NOE list of **1**

NOE	Calculated distance
Arg1HA-Trp2HN	2.64
Trp2HA-Cys3HN	2.21
Cys3HA-HN	2.91
Val4HN-Cys3HA	2.38
Cys16HA-Cys3HA	2.25
Cys3HB1-HB2	1.76
Cys3HB1-Trp17HN	3.02
Cys3HB2-Val4HN	3.36
Val4HA-HB	2.58
Val4HA-HN	2.91
Val4HA-Tyr5HN	2.25
Tyr5HN-Val4HB	2.35
Val4HG1-Tyr5HN	4.03
Tyr5HA-HB1	2.56
Tyr5HA-HN	2.92
Tyr5HA-Ala6HN	2.23
Ala6HA-HB	2.51
Ala6HA-HN	2.93
Ala6HA-Arg7HN	2.25
Ala6HN-HB	3.15
Arg7HN-Ala6HB	3.23
Val8HA-Arg7HB1	3.40
Val8HA-HN	2.80
Arg9HN-Val8HA	2.40
Val8HA-HB	2.59
Arg9HA-HB1	3.02
Arg9HN-HA	2.26
Gly10HN-Arg9HA	2.43

Arg9HA-HB2	2.45
Gly10HA2-HA1	1.79
Gly10HN-HA1	2.75
Gly10HN-Val11HN	2.92
Val11HA-Gly10HN	3.05
Val11HA-HN	2.84
Val11HA-HB	2.48
Val11HN-Gly10HA2	2.38
Arg12HN-Val11HA	2.07
Tyr13HA-Arg14HN	2.22
Arg14HA-HB1	2.42
Arg14HA-HN	2.94
Arg14HA-Arg15HN	2.21
Arg15HA-HB1	2.53
Arg15HA-Cys16HN	2.10
Arg15HN-HB1	2.99
Arg15HN-Arg14HB1	3.01
Cys16HA-Cys3HB1	2.56
Cys16HA-Trp17HN	2.37
Trp17HB1-HB2	1.74
Trp17HN-Cys16HB1	3.47
Tyr5HA-HD1	2.64
Tyr5HA-Arg14HB1	2.56
Val11HB-HG2	2.45
Val11HA-HG2	3.02
Arg14HB2-Tyr5HE1	3.19
Arg14HG1-Tyr5HD1	2.40
Cys3HA-HB2	3.04
Val8HA-HG1	2.93
Arg9HA-HG1	2.56
Arg12HG1-Val11HA	3.37

NOE list of **1a**

**NOE**	**Calculated distance**
Trp2HN-Arg1HA	2.40
Arg1HA-HB1	2.92
Arg1HB1-Trp2HN	3.09

Arg1HG1-HB2	2.32
Trp2HA-Arg1HB1	3.25
Trp2HA-HB1	2.63
Trp2HA-HB2	2.45
Trp2HA-Cys3HN	2.76
Trp2HA-Trp17HB2	2.70
Trp2HB2-HB1	1.74
Cys3HN-HA	2.89
Cys16HA-Cys3HA	2.02
Cys3HA-Trp17HN	2.58
Cys3HB1-HA	2.57
Cys3HA-HB2	3.03
Cys3HB1-HB2	1.75
Val4HN-Cys3HB2	3.59
Val4HA-HB	2.49
Val4HA-Tyr5HN	2.24
Val4HB-HG1	2.46
Val4HN-Cys3HA	2.35
Tyr5HA-HB1	3.05
Tyr5HA-HB2	2.52
Tyr5HA-HN	2.92
Ala6HN-Tyr5HA	2.19
Ala6HA-HB	2.52
Ala6HA-HN	2.89
Ala6HA-Arg7HN	2.29
Arg7HN-Ala6HB	3.23
Arg7HA-HB1	3.03
Arg7HA-HB2	2.42
Val8HN-Arg7HA	2.19
Val8HN-HA	2.94
Ala9HN-Val8HA	2.16
Val8HA-HB	3.05
Val8HA-HG1	3.08
Val8HB-HG1	2.46
Val8HG1-Ala9HN	3.43

Ala9HA-HB	2.50
Ala9HA-HN	2.81
Ala9HA-Arg10NMe	2.54
Ala9HN-HB	2.81
Arg10HA-HG1	2.50
Arg10HA-Val11HN	3.09
Arg10HA-NMe	3.78
Val11HN-Arg10NMe	3.02
Val11HA-HG1	3.01
Val11HA-HN	2.96
Arg12HN-Val11HA	2.25
Val11HA-HB	2.50
Val11HB-Arg12HN	2.53
Val11HB-HG1	2.47
Val8HN-Val11HN	3.41
Arg12HA-HN	2.86
Arg12HA-Tyr13HN	2.24
Arg12HN-HB2	2.75
Tyr13HA-HB1	2.72
Tyr13HA-Arg14HN	2.79
Tyr13HB1-Arg14HN	3.35
Arg14HA-HG1	2.56
Arg14HA-HN	2.94
Arg14HA-Arg15HN	2.18
Arg15HA-HB1	2.76
Arg15HA-HB2	2.45
Arg15HA-Cys16HN	2.16
Cys16HA-Cys3HB1	2.87
Cys16HA-HB1	3.03
Cys16HN-HA	2.98
Trp17HN-Cys16HA	2.09
Cys16HB2-HA	2.49
Trp17HA-HN	2.87
Trp17HB1-HB2	1.74
Tyr5HA-HD1	2.37

Arg14HA-Tyr5HD1	2.69
Arg14HB2-Tyr13HD1	3.00
Arg14HG1-Tyr5HE1	2.83
Trp17HB1-HD1	3.47
Trp17HB1-HE3	3.07

NOE list of **1b**

NOE	Calculated distance
Arg1HA-HB1	2.52
Arg1HA-Trp2HN	2.34
Arg1HB1-HD1	2.47
Trp2HA-Cys3HN	2.23
Trp2HD1-HE1	2.85
Cys3HA-HB2	3.29
Cys3HA-Trp17HN	2.1
Cys3HB2-HB1	1.8
Val4HA-HB	2.45
Val4HB-Tyr5HN	2.63
Val4HG1-HB	2.23
Tyr5HA-HB2	3.04
Tyr5HA-HD1	2.42
Tyr5HA-Ala6HN	2.3
Tyr5HD1-HB2	2.86
Ala6HA-HB	2.21
Arg7HA-HN	3.08
Arg7HA-Val8HN	2.29
Arg7HN-Ala6HB	3.05
Val8HA-HB	2.55
Val8HA-Dva9HN	2.19
Dva9HA-Arg10NMe	1.92
Dva9HB-HG1	2.48
Dva9HB-HN	2.57
Dva9HG1-HN	2.76
Arg10HA-HG1	2.55
Val11HA-HG2	2.64
Val11HA-Arg12HN	1.9
Val11HB-HG2	2.61

Val11HB-HN	2.63
Val11HG1-HG2	2.82
Arg12HA-Tyr13HN	2.25
Arg12HG1-HN	2.6
Tyr13HA-Arg14HN	2.12
Arg14HA-Arg15HN	2.14
Arg14HD1-Tyr13HD1	2.21
Arg15HA-Cys16HN	1.9
Cys16HA-HB2	3.39
Trp17HB2-HD1	2.51
Trp17HE1-HD1	3.01

EXPERIMENTAL SECTION

Protocol for synthesis of peptides having free carboxy terminal (For 1, 1a-f variants):

Peptides were synthesized on TCP resin (1.3 mmolg^{-1}), using standard Fmoc-based chemistry.[2] The C-terminal L/D- Tryptophan residue (1.25 equivalence) was loaded on to the resin with 2.5 equivalence of DIPEA in anhydrous DCM (4 mL) at room temperature. After loading the amino acid, the remaining unreacted trityl chloride groups bound to the solid support were capped using methanol (200 μl/100 mg resin) for 15 min. Next, the resin was thoroughly washed with DCM (3 times), 1:1 DCM-methanol (3 times) and methanol (3 times) and finally dried under vacuum. The loading capacity was estimated from the dry weight of the resin, which ranged from 0.7-1 mmolg^{-1}. The elongation for the rest of the peptide was performed on 150 mg (0.09-0.12 mmol) scale with DIC/HOBt as the coupling agents (2.5 equivalence). All Fmoc deprotections were carried out with 20% piperidine (5 min x 1, 15 min x 1) in DMF.

Protocol for synthesis of peptides having amidated carboxy terminal (2, 2a-f, 3, 3a-f, 4, 4a-f variants):

Peptides were synthesized on Rink Amide AM resin (0.8 mmolg^{-1}) on 200 mg scale (0.16 mmol) using standard Fmoc-based strategy. The resin was swollen in DMF and deprotected with 20% piperidine in DMF (5 min x 1, 15 min x 1) followed by thorough washing with DMF (3 times). The C-terminal amino acid (2.5 equivalence) was loaded onto the resin by using standard coupling reagents (2.5 equivalence of HOBt, 2.5 equivalence of DIC) in DMF for 2.5 h at room temperature. The entire peptide was assembled using this same protocol.

Protocol for generating disulfide bridges:

Peptides were assembled either on Rink Amide AM or TCP resin (depending on the type of C-terminal) with the incorporation of Fmoc-Cys(Acm)-OH at the designated positions, for single disulfide-bridged peptides. Such peptides were cyclized by oxidation using 4 equivalence of iodine in DMF for 2 h. In case of two disulfide-bridge containing peptides, both Fmoc-Cys(Trt)-OH and Fmoc-Cys(Acm)-OH were incorporated in their respective positions in the peptide. First disulfide bridge was generated on-resin as described above with Fmoc-Cys(Acm)-OH, while the second disulfide bridge was generated in solution, after cleaving the peptide from resin, in 0.1 M sodium phosphate buffer (pH 8.0) containing 20% DMSO with overnight stirring.

Protocol for N-methylation:

A modified protocol for Mitsunobu reaction on the solid support was utilized for selective N-methylation of amino acid residue, as described by Chatterjee *et. al.*[3]

Coupling of the amino acid residue following the N-methylated amino acid:

Coupling of Fmoc-Xaa-OH to the free N^{α}-methylamine terminal of the peptides on the resin was carried out using 3 equivalence each of HOAt, HATU and Fmoc-Xaa-OH and 6 equivalence of DIPEA in DMF at room temperature.

Global-deprotection of peptides:

The peptides were cleaved using the cleavage cocktail, TFA:TIPS: H_2O (95:2.5:2.5) for 40 minutes at room temperature with constant stirring at 850 rpm. The cleaved peptide solution was precipitated in chilled diethyl ether, centrifuged twice and the white precipitate was dissolved in water with 10-25% acetonitrile till the solution becomes clear.

Reverse-Phase purification of peptides:

The peptides were purified using a gradient of 15-45% of solvent B, where solvent A is 0.1% TFA in H_2O and solvent B is 0.1% TFA in acetonitrile. TFA-formic acid exchange was performed for peptides to prevent interference of TFA salts in biological and in vivo assays. The exchange was performed by repurifying the pure peptides using the same gradient and solvent system, having 1% formic acid instead of 0.1% TFA.

Circular Dichroism Spectroscopy:

Far-UV CD spectra for all the peptides were recorded at 100 μM concentration in water over a wavelength range of 190-260 nm with a scan rate of 100 nm per minute and a data pitch of 0.5 nm. The raw spectra was smoothened using Savitzky-Golay method.

For experiments with micelles mimicking bacterial membrane environment, SDS was used at a concentration above CMC, i.e. 10 mM. We incubated our peptides for 30 minutes with SDS micelles at room temperature and recorded the CD spectra. Additionally, CD for **4f** was acquired in presence of 50 μM *E.coli* 0111:B4 Lipopolysaccharide prepared in PBS.

Liposome Preparation:

Lipids (POPC, POPE and POPG) and cholesterol were purchased from Avanti Polar Lipids in powder form and were dissolved in chloroform/methanol (2:1) solution to yield 20 mM concentration. The chloroform/methanol solutions of POPE and POPG were mixed in a ratio of 7:3, whereas POPC and cholesterol were mixed in a ratio of 10:1 mimicking both bacterial and mammalian membrane, respectively. The solutions were dried overnight in vacuum. The dried thin mixture films were rehydrated in PBS yielding a 20 mM lipid solution, followed by rapid freeze-thawing for 8 cycles to produce suspension of multilamellar vesicles (MLVs). Such MLVs suspension was then extruded 21 times using a Mini Extruder (Avanti Polar Lipid Inc.) through a 0.1 mm pore size polycarbonate membrane filter to obtain large unilamellar vesicles (LUVs) suspension. LUVs size was confirmed through DLS measurements.

Fluorescence quenching with liposomes:

Fluorescence quenching experiments were conducted by titrating the peptides with water-soluble collisional quencher of tryptophan, acrylamide in the absence or presence of liposomes. To reduce the absorbance by acrylamide, excitation wavelength of Trp was changed to 290 nm keeping the emission wavelength range constant. Aliquots from 4 M acrylamide stock were titrated into peptide solution (3 μM) both in absence and presence of liposomes, where Lipid/Peptide molar ratio was kept 300. The spectrum obtained was an average of three scans, which was further corrected for dilution and scattering derived from acrylamide titration of vesicle blank. Stern-Volmer equation was employed for data analysis-

$$F_0/F = 1 + K_{SV}\cdot[Q]$$

Where, Fo and F are the fluorescence intensities in absence and presence of the acrylamide (Q), respectively and K_{sv} is the Stern-Volmer quenching constant, which is a measure of the accessibility of tryptophan to quenchers.

NMR Experiments:

For the compounds nos.**1,1a,1b,1c,1d,1e,** NMR was acquired in H_2O:D_2O (9:1). In all the compounds, 0.1% TMSP was used as an internal standard (δ = 0 ppm). Standard Bruker pulse sequences zgesgp for ^{1}H, mlevesgpph/dipsi2rcesgpph (60 ms mixing time) for TOCSY, roesyesgpph (100 ms mixing time) for ROESY were used to acquire the NMR data.Two-dimensional data were obtained using 2048 data points in the direct dimension and 512 data points in the indirect dimension. NMR for the compounds were acquired at 25°C using a concentration of 0.5-1 mM.

All NMR data were processed using iNMR (www.inmr.net), and the 2D NMR data were analyzed with SPARKY (T. D. Goddard and D. G. Kneller, SPARKY 3, University of California, San Francisco). The chemical shift tables were generated from TOCSY and ^{1}H spectra. The sequential assignments and inter- and intra-residue NOEs were determined through ROESY. The NOEs were then integrated, and the integration values were converted to distances using the formula $V=Kd^{-6}$, where V is the integrated peak volume, K is a constant (determined using resolved diastereotopic CH_2 groups from Tyr/Trp or in some cases HA-HB distance of Ala), and d is the distance between the protons.

For compound nos. **1,1a,1b,1c,1d,1e,** the secondary chemical shift was determined by subtracting the random coil values reported by Wishart *et. al.* from the observed chemical shifts values.

Structure Calculation:

To calculate the structure of **1,1a,** and **1b,** we have used charmM force field via the interface of Discovery Studio for the entire process. The distance restraints were converted into a charm restraint file using a custom Perl script. The resulting file was then used to define NOE restraints inside the charmM syntax. To the distance, 10% were added or subtracted to define the upper and lower limits respectively. If there were any methyl protons involved in the restraints, an additional 0.4 Å per methyl group (pseudoatom correction) were added to the upper limit to compensate for the errors involved.

To begin the process, firstly a linear molecule was created in Discovery Studio and solvated employing an explicit water box with water boundary of 12 Å . It was then subjected to a simulated annealing run, where the distance restraint files were given as input. The obtained structure was refined by dihedral angle constraints derived from ^{1}H NMR spectra employing Bystrov equation followed by a long 200 ns

restrained molecular dynamics run in implicit solvent (for **1**,**1a** and **1b** dielectric used is 80). The average over the dynamics run was considered to be the final structure and 10 structures were sampled at equal time intervals to generate the ensemble. Violations across 200 conformations generated in long run were calculated using custom PERL script, where any distance restraint displaying violations >1 was corrected for, and the above steps were repeated again.

Computer Simulations:

All molecular dynamics simulations were performed using the GROMACS 2024.3[4, 5] software package and the CHARMM36m forcefield.[6] The initial peptide conformations for the simulations of the cysteine-containing variants (**1**, **1a**, **1b**) were taken from the experimental NMR data. For the threonine-substituted variants (**1c**, **1d**, **1e**), cysteine residues were replaced with threonine using CHARMM-GUI PDB reader.[7] Each system was solvated with modified TIP3P water[8] and neutralized with appropriate counterions (Na+ or Cl−) to achieve a physiological background concentration of 0.15 M NaCl. All peptide structures were visualized and analyzed using VMD[9] and PyMOL.[10]

The V-rescale thermostat[11] was used for all the simulations to maintain a constant temperature of 310 K, and periodic boundary conditions were employed in all directions. Initially, energy minimization was carried out for all the systems using the steepest descent algorithm until the magnitude of all forces were below 1000 kJ/(mol·nm). Long-range electrostatics were handled using the Particle Mesh Ewald (PME) method[12, 13] with a cutoff of 1.2 nm. The van der Waals interactions were treated using the Verlet cutoff scheme with a 1.2 nm cutoff.

Simulation Protocols:

Aqueous Solution Simulations

The single-chain simulations of peptides (**1**, **1a**, **1b**, **1c**, **1d**, and **1e**) in water were performed in a 5 nm cubic box. Following energy minimization, the systems were equilibrated in two phases: a 100 ps NVT (constant number of particles, volume, and temperature) run, followed by a 100 ps NPT (constant number of particles, pressure, and temperature) run using a Parrinello-Rahman Barostat with isotropic pressure coupling. For the cysteine-based peptides, a production run of 1 microsecond was performed for each system. For the threonine-substituted peptides (**1c**, **1d**, **1e**), three independent simulations each of 2.5 microseconds were performed for each peptide.

Membrane Interaction Simulations

The simulations for studying the interaction of the peptides with the membranes were performed by placing the peptide at the interface of model membranes constructed in a 10 nm cubic box. The systems were constructed using the CHARMM-GUI Membrane Builder.[14] The membrane model mimicking the bacterial membrane consisted of a bilayer of POPE:POPG in a 7:3 ratio, whereas the membrane model mimicking the mammalian membrane consisted of a bilayer of POPC:Cholesterol in a 9:1 ratio. Simulations were performed for cysteine-containing (**1**, **1a**, **1b**) and threonine-substituted (**1c**, **1d**, **1e**) peptides. In each simulation, four different conformations of the peptide were placed on the membrane surface with dimensions 10 nm X 10 nm, well separated from each other to generate sufficient statistics to compute the peptide-membrane interaction properties.

The equilibration protocol of the peptide-membrane systems involved multiple steps. The position restraints on the protein and lipids were decreased gradually in a series of four 100 ps NVT runs. This was followed by a 1 ns NPT equilibration using the Parrinello-Rahman Barostat with a semi-isotropic pressure coupling, which allows for independent scaling of the z-dimension (perpendicular to the membrane plane). The production runs for the cysteine-based peptide systems were 1 microsecond each, while the threonine-substituted systems were run for 500 ns each. The simulation time step is 2 fs, and all the hydrogen bonds were constrained using the LINCS algorithm.[15] The center-of-mass motion of the protein-membrane system was removed to prevent system drift.

Simulation Data Analysis:

Secondary Structure Content

The secondary structure content in the peptides was analyzed using the VMD-ss plugin, which employs the STRIDE[16] algorithm to identify the secondary structural elements such as α-helices, β-sheets, turns, and coils based on backbone dihedral angles and hydrogen bonding patterns. The percentage composition of each secondary structure was calculated and analyzed over time for different peptide systems to evaluate the impact of turn-engineering and environment on peptide conformations.

Solvent Accessible Surface Area (SASA)

The SASA was calculated to quantify the peptides' exposure to the solvent. A decrease in SASA typically indicates a transition to a more compact or buried state, such as folding into a β-hairpin or inserting into a membrane, where the peptide exposure to the solvent decreases.

Radial Distribution Function

The radial distribution function (g(r)) was computed to investigate the local density of specific lipid headgroups around key peptide residues. The g(r) is the probability of finding a particle (e.g., a lipid headgroup atom) at a distance r from a reference particle (e.g., a peptide residue atom), relative to a uniform distribution.

Z-component Distance between Membrane and AMP

The Z-component distance between the center of mass (COM) of the peptide and the center of mass of the lipid bilayer was tracked over time. This metric provides a direct measure of the peptide's position relative to the membrane plane. A low Z-component distance indicates that the peptide is buried into the membrane. In contrast, fluctuations in the distance can signify processes like interaction or detachment of the peptide from the membrane.

Energy Computation

The interaction energy between the peptide and membrane components was computed to quantify the binding affinity and identify the driving forces for the interaction. Using GROMACS, the production trajectories were re-analyzed by defining energy groups for the protein and different membrane components. This allowed for calculating residue-specific Lennard-Jones (van der Waals) and Coulomb (electrostatic) interaction energies between the peptide and the individual lipid components. These calculations were performed by rerunning the trajectories and specifying the interaction groups, followed by post-processing with gmx energy to obtain the energy contributions for each residue.

Retention Time Analysis:

To assess the relative hydrophobicity of peptides, peptides were ran on a binary gradient of 10% to 50% acetonitrile/water for 20 minutes in C-18 (250 mm x 4.6 mm I.D., 5 µm) column at a flow rate of 1 mL min^{-1}.

Proteolytic Digestion of Peptides:

The metabolic stability of peptides (**1,1d,1f** and **3,3d,3f**) were evaluated against two proteases-chymotrypsin and trypsin at a concentration of 100 µM. The metabolic stability of **4f** was evaluated by incubating 100 µM of peptide with a more promiscuous enzyme i.e. Proteinase K. Peptides were incubated with different enzyme to peptide ratio for different time intervals at 37 °C. After every time-point, a 20 µL reaction solution was aliquoted and heat-inactivated at 100 °C for 5 minutes. The solution was stored at -80 °C till the time of analysis by HPLC. In case of chymotrypsin, the reaction was

performed in 100 mM Tris-Cl, pH 7.8 at a ratio of 1:200 (peptide:enzyme). For trypsin digestion, the assay was carried out in 100 mM Tris-Cl, pH 8.5 using a ratio of 1:100. For proteinase K digestion, the assay was done in 1X TBS buffer at pH 7.5 at a ratio of 1:200. For every time point, 50 μL of reaction mixture was aliquoted and mixed with 150 μL chilled ACN, then centrifuged and the supernatant was lyophilized and injected into HPLC. The samples injected in HPLC were resolved using a 10-50% ACN/H_2O gradient. All the peaks were collected and subjected to characterization by mass-spectrometry.

Bacterial Strains and growth media:

Antibiotic-sensitive bacterial strains used in the study included gram-positive bacteria Methicillin-sensitive *Staphylococcus aureus* ATCC 25923 and gram-negative bacteria *Escherichia coli* ATCC 35218*, Escherichia coli* K12 MG1655*, Pseudomonas aeruginosa* ATCC 27853*, Acinetobacter baumannii* MTCC 9829*, Acinetobacter baumannii* ATCC 19606 (MCC 2076) and *Klebsiella pneumoniae* MTCC 7407*.* The clinical isolate used for mice infection studies was *NDM-harbouring Acinetobacter baumannii* RPTU 61. Bacterial strains were cultivated in Mueller-Hinton medium following CLSI guidelines.

Assays employing drug-resistant strains were performed by Anthem Biosciences, Bangalore, India. Both gram-positive and gram-negative resistant bacteria were included, and the details are enlisted in Figure 5b. All these strains were revived and cultivated in Soybean Casein Digest Agar medium.

Further susceptibility assays against more clinical isolates of *Acinetobacter baumannii* and *Staphylococcus aureus* were done at IIT, Roorkee with details in Supporting Information Figure S21. All strains were revived using standard Luria-Bertani agar and then, all *A. baumannii* isolates were cultivated in Leeds agar and all *S. aureus* isolates were cultivated using Mannitol salt agar.

Antimicrobial assays:

The antimicrobial potency of all the peptides were evaluated using a microbroth dilution assay reported by Hancock *et. al.,* with some minor modifications.[17] Briefly, the peptides/antibiotics were serially diluted 2-fold in 0.5% of BSA solution or in MH media in a 96-well polystyrene microplate (50 mL), with a starting concentration of either 50 μM or 12.5 μM. Overnight bacterial suspension was adjusted accordingly to 0.5 McFarland Standard, which was further diluted to $1x10^6$ cfu/mL. From it, 50 μL was aliquoted into peptide/antibiotic containing well such that the final cell density is $5x10^5$ cfu/mL and incubated for 20-24 hours at 37°C. Untreated bacterial culture and fresh medium were included as growth control and

sterile control, respectively. After 24 h, bacterial growth was monitored by measuring the turbidity at 600 nm, using a ThermoFisher microplate reader. The lowest peptide concentration, where there is no visible turbidity was termed as the Minimal Inhibitory Concentration (MIC). For Minimum Bactericidal Concentration (MBC) determination, wells corresponding to 1x, 2x and 4x of MIC were plated on MH agar plate to determine viable bacteria after incubation for 16 hours at 37 °C. The concentration at which there were <10 colonies, was considered as MBC for each peptide. All MIC and MBC values reported were median of 4-5 independent experiments. For comparison, MICs of polymyxin B and vancomycin were also determined. For MIC determination of log-phase culture, an overnight bacterial culture was grown to an exponential phase (OD_{600} -0.6), which was used to perform the assay.

MICs for drug-resistant strains were performed by Anthem Biosciences., employing their in-house standardised protocol. All their assays were performed using cation adjusted MH media (CAMHB) and the data were recorded after 24 h. Similarly, MICs against clinical isolates performed by IIT, Roorkee employed standard protocol in which experiment was done following CLSI guidelines. CAMHB was used and data recorded after 18 h. the lowest concentration which displayed no visible turbidity was considered the MIC.

Hemolysis Assay:

The hemolysis assay was performed against freshly isolated human RBCs (hRBCs). The blood samples were collected in an EDTA-coated vial and centrifuged at 2000g for 5 minutes to isolate the RBCs. The hRBCs were washed thrice with Phosphate Buffered Saline (PBS) and dissolved to a final stock of 20% (v/v) hRBCs. Meanwhile, the peptides were diluted 2-fold in PBS in a 96-well plate with a starting concentration of 200 μM, to which hRBCs were added such that the final concentration became 4% (v/v). The plates were incubated for 1.5 h at 37 °C with stirring. hRBCs in buffer and 0.1% Triton-X-100 were employed as negative and positive control, respectively. The plate was centrifuged at 4000 rpm for 20 minutes and 50 μL aliquots of supernatant was transferred to a fresh 96-well plate. The extent of release of haemoglobin from the human erythrocytes were monitored at 405 nm. The percentage of hemolysis was calculated as :

$$\text{Percentage of hemolysis (\%)} = [(OD_{sample}-OD_{buffer})/(OD_{triton}-OD_{buffer})]*100$$

The experiment was performed in triplicates and repeated twice using same blood. The data is represented as an average of triplicate experiment with standard error. The peptide concentration that demonstrated ≤ 10% hemolysis, was considered as Minimum Hemolytic Concentration (MHC).

The study was reviewed and approved by the Institutional Human Ethics Committee (IHEC) of the Indian Institute of Science, Bangalore. (Approval number: IHEC: 4-14032018).

PBMC cytotoxicity assay:

The cytotoxicity of human peripheral blood mononuclear cells(PBMCs) was evaluated using fluorescence-based resazurin assay as described.[18] Briefly, blood samples (10 mL) were collected from healthy individuals and fresh PBMCs were isolated using HiSep™ LSM077 (Himedia). $2x10^5$ cells were seeded in RPMI medium supplemented with 10% fetal bovine serum and L-glutamine (300 mg/L) in culture-grade 96-well plates for 24 h. Cells were treated with 10-, 20- and 40-times MIC of Polymyxin B and **4f** against *A. baumannii*. Cells treated with 1% Triton-X-100 was taken as complete lysis control and cells treated with buffer were taken as 100% growth control. The percentage of viability was calculated as:

$$\text{Percentage of Viability (\%)} = [(OD_{sample}-OD_{buffer})/(OD_{triton}-OD_{buffer})]*100$$

Isothermal titration calorimetry (ITC) analysis:

ITC was performed to determine the binding affinity and thermodynamic parameters of **4** and **4f** with *E. coli* 0111: B4 Lipopolysaccharide (LPS) micelles using a TA-affinity ITC (TA instruments, Waters Inc. New Castle, DE). All peptides and LPS were dissolved in 10 mM phosphate buffer at pH 7.4 and degassed. A sample cell (volume 182 µL) containing 0.05 mM LPS was titrated against 0.5 mM peptides at 298 K. A total of 30 injections were carried out at an interval of 2 minutes with 2 µL of peptide aliquots per injection. The raw data were plotted using NanoAnalyze 3.7.5 software supplied with the instrument. A 'multiple site binding' model was used to analyse the Stoichiometry (n), binding constant (K_a and K_d), the change in heat of enthalpy (ΔH) and entropy (ΔS) of reaction. The Gibbs free energy of binding (ΔG) was evaluated using the equation $\Delta G = \Delta H - T\Delta S$.

Real-time monitoring of Membrane Permeability:

The membrane integrity was assessed by real-time monitoring the uptake of Propidium Iodide (PI) by bacterial cells using flow-cytometry. Overnight cultures of *Acinetobacter baumannii* MTCC 9829 and *S. aureus* ATCC 25923 were diluted to OD_{600} - 0.7. The cell suspension was treated with different concentrations of peptide for 10 minutes at 37 °C. After treatment, the cells were centrifuged at 6000 rpm for 10 minutes. The cells were washed once in PBS and resuspended in 100 µL and treated with

2 μg/mL PI for 2 minutes in ice. The experiment was performed in duplicates and for control, untreated bacterial suspension in PBS was employed

Bacterial Kill Kinetics Assay:

A time-kill assay was performed to assess whether the membrane damage ultimately leads to bacterial death. The assay was designed based on a protocol reported by Mwangi *et. al.,* with some slight modifications.[19] Overnight cultures of *S. aureus* ATCC 25923 and *A. baumannii* MTCC 9829 were diluted to $1x10^6$ cfu/mL in MH broth. 0.5x, 1x, 2x and 5x of MIC of 4f were added to the bacterial suspension in exponential phase and incubated at 37 °C under shaking conditions. 100 mL was aliquoted out at various time points- 0, 0.25, 0.5, 1, 2, 5, 7 and 24 h. Such aliquots were serially diluted in MH media, plated in MH agar plates and incubated for 18-20 h at 37 °C. After incubation, viable colonies were determined.

SEM Imaging:

S. aureus ATCC 25923 and *A. baumannii* ATCC 19606 (MCC 2076) samples were analyzed for peptide mediated cell damage. For SEM sample preparation, overnight grown bacterial cultures were diluted as 1:10 to fresh medium. After about 3 h of incubation, cultures were diluted to 0.5 OD and treated with 5 times MIC of **4f** specific for each organism for 30 mins. Then, the cells were washed three times with PBS and spotted on poly-L-lysine coated glass coverslips. Cells were fixed with 4% paraformaldehyde, and further with 2.5% glutaraldehyde in sodium cacodylate buffer (0.1 M, pH 7.2) for 10 min at 25 °C. To dehydrate the cells, coverslips were treated with increasing concentrations of ethanol (25, 50, 75, and 96%) and kept overnight in vacuum. Samples were coated and observed using SE2 detector operation at Secondary Electron mode by ULTRA 55 UHR-SEM (Zeiss).

Laboratory-evolution of bacterial resistance experiment:

The evolutionary protocol employed was reported by Csaba Pal and his group, but with some modifications.[20] The protocol ensured that the populations with highest resistance were propagated further. *Acinetobacter baumannii* MTCC 9829, *Staphylococcus aureus* ATCC 25923 and *Pseudomonas aeruginosa* ATCC 27853 were used for laboratory evolution of resistance. To begin with, **4f** and two standard antibiotics, polymyxin B and vancomycin were 2-fold serially diluted in MH media (100 μL). To a drug-containing well, 10% of 0.1 OD of overnight bacterial suspension was added and rest of the volume was adjusted by media to make the final volume to 200 μL per well. This was considered as Passage 0. Six parallel populations per drug for each species were used to generate resistance. The

plate was incubated in shaking conditions (~180 rpm) for 20-24 h at 37°C. Next day, passage MICs were recorded and for Passage 1, 0.1% inoculum from the highest drug concentration showing growth (OD_{600} >0.1) was transferred to a fresh plate containing serially diluted drug with a final volume of 200 μL. This routine was repeated for 20 transfers. During each transfer, glycerol stocks of the previous passage was prepared and stored at -80°C. After 20th passage, stability studies were ensured for six parallel populations for each species by culturing them in drug-free media and then, evaluating their MIC following aforementioned protocol.

Checkerboard Assay:

A checkerboard assay was performed to evaluate the efficacy of **4f** alone or its synergistic, additive or antagonistic effect when used in combination with Polymyxin B or Vancomycin against laboratory-evolved resistant strains, Polymyxin B-resistant *Acinetobacter baumannii* MTCC 9829, Polymyxin B-resistant *Pseudomonas aeruginosa* ATCC 27853 and Vancomycin-intermediate *Staphylococcus aureus* ATCC 25923.

Briefly, **4f** and Polymyxin B (in case of *A. baumannii* and *P. aeruginosa*)/ Vancomycin (in case of *S. aureus*) were diluted in horizontal and vertical direction, respectively in separate 96-well plates by two-fold dilution method. Next, 50 μL of 4f and Polymyxin B/Vancomycin different dilutions were correspondingly added to a new plate, keeping the direction of addition similar to direction of dilution of the drug. This yielded a checkerboard pattern having two drug concentrations in different proportions. The column and the row corresponding to 4f alone or Polymyxin B/Vancomycin alone, an additional 50 μL media was added to make the final volume to 100 μL. The checkerboard assay was performed in Mueller-Hinton Broth with a final volume in each well to be 200 μL. So, 100 μL of $5x10^5$ cfu/mL inoculum, prepared from an overnight bacterial suspension were added to each well and incubated for 20-24 h at 37°C. Bacterial growth was measured and MICs of the two drugs in alone and in combination were determined. From the results, FICs were calculated as follows:

$$\sum FIC = FIC_{DrugA} + FIC_{DrugB}$$

FIC_{drugA} = MIC of Drug A in combination/MIC of Drug A alone

FIC_{drugB} = MIC of Drug B in combination/MIC of Drug B alone

The results were interpreted in the following terms: $\Sigma FIC \leq 0.5$ is considered synergistic, $0.5 < \Sigma FIC < 4$ is considered indifferent/additive and $\Sigma FIC \geq 4$ is considered antagonistic. The experiment was independently performed in duplicates and the values reported is a median of two experiments.

Measurement of membrane-surface charge of laboratory evolved resistant bacterial strains:

To evaluate whether there is any change in the net surface charge of bacterial strains (*A. baumannii, S. aureus* and *P. aeruginosa*) after 20 passages of resistance evolution using **4f**, we performed fluorescein isothiocyanate-labelled Poly-L-Lysine (FITC-PLL, Sigma) binding assay. FITC-PLL is a polycationic molecule used to investigate the interaction between cationic peptides and negatively charged bacterial membrane. Briefly, bacterial strains belonging to 20th passage were grown overnight, centrifuged, washed and diluted to 0.1 OD_{600} in 1mL of PBS. A freshly prepared FITC-PLL solution was added to the bacterial suspension at a final concentration of 6.5 μg/mL. The suspension was incubated at RT for 10 minutes and was centrifuged at 6000 rpm for 15mins. The amount of FITC-PLL remaining in the supernatant was measured fluorometrically (excitation at 500 nm and emission at 530 nm) with or without bacterial exposure. The amount of FITC-PLL bound was determined by the difference between these values. A lower value indicates less net negative charge on the surface of the bacterial membrane.

Mice and Ethics statement:

BALB/c mice were bred and maintained in the Central Animal Facility of IISc Bangalore. For all the experiments, 6-8 weeks old female mice were used for each condition. All experiments were conducted in accordance with the Control and Supervision rules 1998 of the Ministry of Environments and Forests Act (Government of India) and the Institutional Animal Ethics Committee of IISc (Reg. number: 48/1999/CPCSEA, Date: 1/3/1999). Experimental protocols conducted were approved by the 'Committee for Purpose and Control and Supervision of Experiments on Animals' (CPCSEA) with the permit number: CAF/Ethics/928/2022. The details of the national guidelines which are followed can also be found on the website: http://envfor.nic.in/division/committee-purpose-control-and-supervision-experiments-animals-cpcsea.

Acute *in-vivo* toxicity assessment:

Female BALB/c mice of 6-8 weeks were taken and weighed. Grouping randomization on the basis of body weight was done using the software RandoMice v1.1.5. Before administering the peptide, weight, fur coat (photograph), mobility and activeness (3 min. video recording) were monitored. Then **4f** was administered i.p. in 2 split doses (10, 20 and 40 mg/kg/day) after 2nd h and 12th h , respectively. The weight, fur coat (photograph), mobility and activeness (3 min. video recording) of all mice were monitored and recorded. Also, motility was checked at every 12 h till 72 h as a function of toxicity.

***In vivo* efficacy in mice model:**

Female BALB/c mice (n=5 per group) were made neutropenic by intraperitoneal administration of 150 mg/kg cyclophosphamide and 100 mg/kg at 3 days and 1 day before infection respectively. Infection was induced peritoneally by I.P. administration of $1x10^9$ cfu/ml carbapenem resistant Acinetobacter baumannii (NDM-harbouring *A. baumannii* RPTU 61 clinical isolate). Mice were treated with vehicle (PBS control) and 20 mg/kg of **4f** by I.P. injection post 2 hours of infection. After 8 h of treatment, peritoneal fluid, spleen and lungs were harvested from all mice. The organs were homogenized in PBS and plating to determine cfu count was done on the same day on Leeds agar that is selective for *A. baumannii*.

Mice survival experiment:

Neutropenic female BALB/c mice (n=8) were infected as mentioned above. They were treated with 10 mg/kg and 20 mg/kg of **4f** through I.P. administration. All mice were examined after 8 h, 16 h and 24 h of treatment for any mortality.

Rapid Equilibrium Dialysis (RED) assay:

Plasma was extracted as mentioned in the previous section. Plasma was spiked with 100 µM peptide sample, volume was adjusted to 500 µL. The spiked plasma was added to the sample chamber, indicated by the red coloured ring. In the other chamber, 750 µL (as mentioned in the Thermo Fischer Scientific protocol) dialysis buffer (pH 7.4) containing 100 mM sodium phosphate, 150 mM sodium chloride was added. This entire unit was covered with a sealing tape and incubated at 37˚C for 4 h under mild shaking condition (300 rpm). After 4h, the unit was taken out, the seal was removed, and volume loss was checked (if any). Equal volumes (250 µL) of sample and buffer was aliquoted from both the chambers. To make the conditions and reaction constituents uniform, a certain volume of dialysis buffer was added to the aliquoted spiked plasma and the same volume of fresh plasma was added to the aliquoted sample from the buffer chamber. Chilled ACN was added to both to precipitate proteins and release peptides. Samples were centrifuged for 10 mins at 13,000 *g*, the supernatant was separated and used for HPLC quantification. The test compound concentration was measured in both buffer and plasma chamber through relative peak area analysis. Percentage of plasma bound peptide was calculated using the following formula:

% Free = (Peptide conc. in buffer chamber/Peptide conc. in plasma chamber) x 100

% Bound = 100 - % Free

REFERENCES

1. P. Ghosh, N. Raj, H. Verma, M. Patel, S. Chakraborti, B. Khatri, C. M. Doreswamy, S. R. Anandakumar, S. Seekallu, M. B. Dinesh, G. Jadhav, P. N. Yadav and J. Chatterjee, *Nat Commun*, 2023, **14**, 6050.

2. P. Lahiri, H. Verma, A. Ravikumar and J. Chatterjee, *Chem. Sci.*, 2018, **9**, 4600-4609.

3. J. Chatterjee, B. Laufer and H. Kessler, *Nat. Protoc.*, 2012, **7**, 432-444.

4. D. Van der Spoel, E. Lindahl, B. Hess, G. Groenhof, A. E. Mark and H. J. C. Berendsen, *J. Comput. Chem.*, 2005, **26**, 1701-1718.

5. H. J. C. Berendsen, D. Vanderspoel and R. Vandrunen, *Comput. Phys. Commun.*, 1995, **91**, 43-56.

6. J. Huang, S. Rauscher, G. Nawrocki, T. Ran, M. Feig, B. L. de Groot, H. Grubmüller and A. D. MacKerell, *Nat. Methods*, 2017, **14**, 71-73.

7. S. Jo, T. Kim, V. G. Iyer and W. Im, *J. Comput. Chem.*, 2008, **29**, 1859-1865.

8. D. J. Price and C. I. I. I. Brooks, *J. Chem. Phys.*, 2004, **121**, 10096-10103.

9. W. Humphrey, A. Dalke and K. Schulten, *J. Mol. Graph. Model*, 1996, **14**, 33-38.

10. W. L. DeLano, *Abstr. Pap. Am. Chem. Soc.*, 2004, **228**, U313-U314.

11. G. Bussi, D. Donadio and M. Parrinello, *J. Chem. Phys.*, 2007, **126**, 014101.

12. U. Essmann, L. Perera, M. L. Berkowitz, T. Darden, H. Lee and L. G. Pedersen, *J. Chem. Phys.*, 1995, **103**, 8577-8593.

13. T. Darden, D. York and L. Pedersen, *J. Chem. Phys.*, 1993, **98**, 10089-10092.

14. S. Jo, J. B. Lim, J. B. Klauda and W. Im, *Biophys. J.*, 2009, **97**, 50-58.

15. B. Hess, H. Bekker, H. J. C. Berendsen and J. G. E. M. Fraaije, *J. Comput. Chem.*, 1997, **18**, 1463-1472.

16. D. Frishman and P. Argos, *Proteins*, 1995, **23**, 566-579.

17. I. Wiegand, K. Hilpert and R. E. Hancock, *Nat. Protoc.*, 2008, **3**, 163-175.

18. M. Saini, A. Gaurav, A. Hussain and R. Pathania, *ACS Infect. Dis.*, 2024, **10**, 1711-1724.

19. J. Mwangi, Y. Yin, G. Wang, M. Yang, Y. Li, Z. Zhang and R. Lai, *Proc. Natl. Acad. Sci USA*, 2019, **116**, 26516-26522.

20. V. Lázár, A. Martins, R. Spohn, L. Daruka, G. Grézal, G. Fekete, M. Számel, P. K. Jangir, B. Kintses, B. Csörgo, A. Nyerges, A. Györkei, A. Kincses, A. Dér, F. R. Walter, M. A. Deli, E. Urbán, Z. Hegedus, G. Olajos, O. Méhi, B. Bálint, I. Nagy, T. A. Martinek, B. Papp and C. Pál, *Nature Microbiology*, 2018, **3**, 718-731.
